# Different inhaled corticosteroid doses in triple therapy for chronic obstructive pulmonary disease: systematic review and Bayesian network meta-analysis

**DOI:** 10.1038/s41598-022-18353-y

**Published:** 2022-09-20

**Authors:** Hyun Woo Lee, Hee Moon Park, Eun Jin Jang, Chang-Hoon Lee

**Affiliations:** 1 Division of Pulmonary and Critical Care Medicine, Department of Internal Medicine, Seoul Metropolitan Government-Seoul National University Boramae Medical Center, Dongjak-gu, Seoul, Republic of Korea; 2 Division of Pulmonary and Critical Care Medicine, Department of Internal Medicine, Seoul National University Hospital, 101 Daehak-Ro Jongno-Gu, Seoul, 03080 Republic of Korea; 3 Department of Information Statistics, Andong National University, Andong, Republic of Korea

**Keywords:** Respiratory tract diseases, Outcomes research

## Abstract

A systematic review and Bayesian network meta-analysis is necessary to evaluate the efficacy and safety of triple therapy with different doses of inhaled corticosteroids (ICS) in stable chronic obstructive pulmonary disease (COPD). We selected 26 parallel randomized controlled trials (41,366 patients) comparing triple therapy with ICS/long-acting beta-agonist (LABA), LABA/long-acting muscarinic antagonist (LAMA), and LAMA in patients with stable COPD for ≥ 12 weeks from PubMed, EMBASE, the Cochrane Library, and clinical trial registries (search from inception to June 30, 2022). Triple therapy with high dose (HD)-ICS exhibited a lower risk of total exacerbation in pre-specified subgroups treated for ≥ 48 weeks than that with low dose (LD)-ICS (odds ratio [OR] = 0.66, 95% credible interval [CrI] = 0.52–0.94, low certainty of evidence) or medium dose (MD)-ICS (OR = 0.66, 95% CrI = 0.51–0.94, low certainty of evidence). Triple therapy with HD-ICS exhibited a lower risk of moderate-to-severe exacerbation in pre-specified subgroups with forced expiratory volume in 1 s < 65% (OR = 0.6, 95% CrI = 0.37–0.98, low certainty of evidence) or previous exacerbation history (OR = 0.6, 95% CrI = 0.36–0.999, very low certainty of evidence) than triple therapy with MD-ICS. Triple therapy with HD-ICS may reduce acute exacerbation in patients with COPD treated with other drug classes including triple therapy with LD- or MD-ICS or dual therapies.

## Introduction

Chronic obstructive pulmonary disease (COPD) is as an important chronic inflammatory airway disease, but its treatment is still challenging. As COPD is characterized by persistent airflow limitation, bronchodilators including long-acting beta-agonists (LABAs) and long-acting muscarinic antagonists (LAMAs) have been the main treatment modalities. While inhaled corticosteroid (ICS) therapy, a key therapy modality for asthmatics, was reported to be less effective in COPD treatment^1–4^, it has been reported that ICS-containing combination therapy is effective in COPD. Randomized controlled trials (RCTs) have shown that combination therapy with ICS/LABA reduces the risk of acute exacerbation^5,6^. Currently, it is evident that triple therapy with ICS/LABA/LAMA has the best efficacy in terms of reducing acute exacerbation and mortality as well as improving symptoms and lung function among drug classes, especially among those with previous exacerbation history and elevated blood eosinophil counts^7–9^.

However, the ICS dose for triple therapy in patients with COPD has not been determined. ICS is associated with an increased risk of pneumonia^10,11^. The increased risk of pneumonia due to ICS has been reported primarily in studies using triple therapy with high dose (HD)-ICS^12^; low dose (LD)-ICS triple therapy was not associated with pneumonia risk^13^. Thus, there are concerns about using higher dose ICS-containing regimens^13^. Furthermore, a ceiling efficacy has been reported for ICSs. In asthmatics, the maximum level of efficacy was usually reached with LD-ICS, while HD-ICS did not show additional benefit^14,15^. This suggests that a high dose-ICS is not beneficial considering risk and benefit. However, a recent network meta-analysis (NMA) with triple therapy in uncontrolled asthma reported that HD-ICS showed superiority in reducing moderate-to-severe exacerbation and improving forced expiratory volume in one second (FEV_1_) compared to triple therapy with medium dose (MD)-ICS^16^. For patients with COPD, only one RCT has reported that there were no significant differences in the risk of exacerbation and mortality between triple therapy with MD-ICS and that with LD-ICS^17^. The ICS dose with the best efficacy and safety and the patient stratification parameters for guiding ICS dose determination in triple therapy are unclear.

Therefore, we conducted a systematic review (SR) and NMA to compare triple therapies with LD-, MD-, and HD-ICS with reference to efficacy (including exacerbation and mortality) and safety; we also sought to identify specific subgroups which may derive benefit from specific ICS dose levels.

## Results

### Study selection and network structure

**Figure 1:**
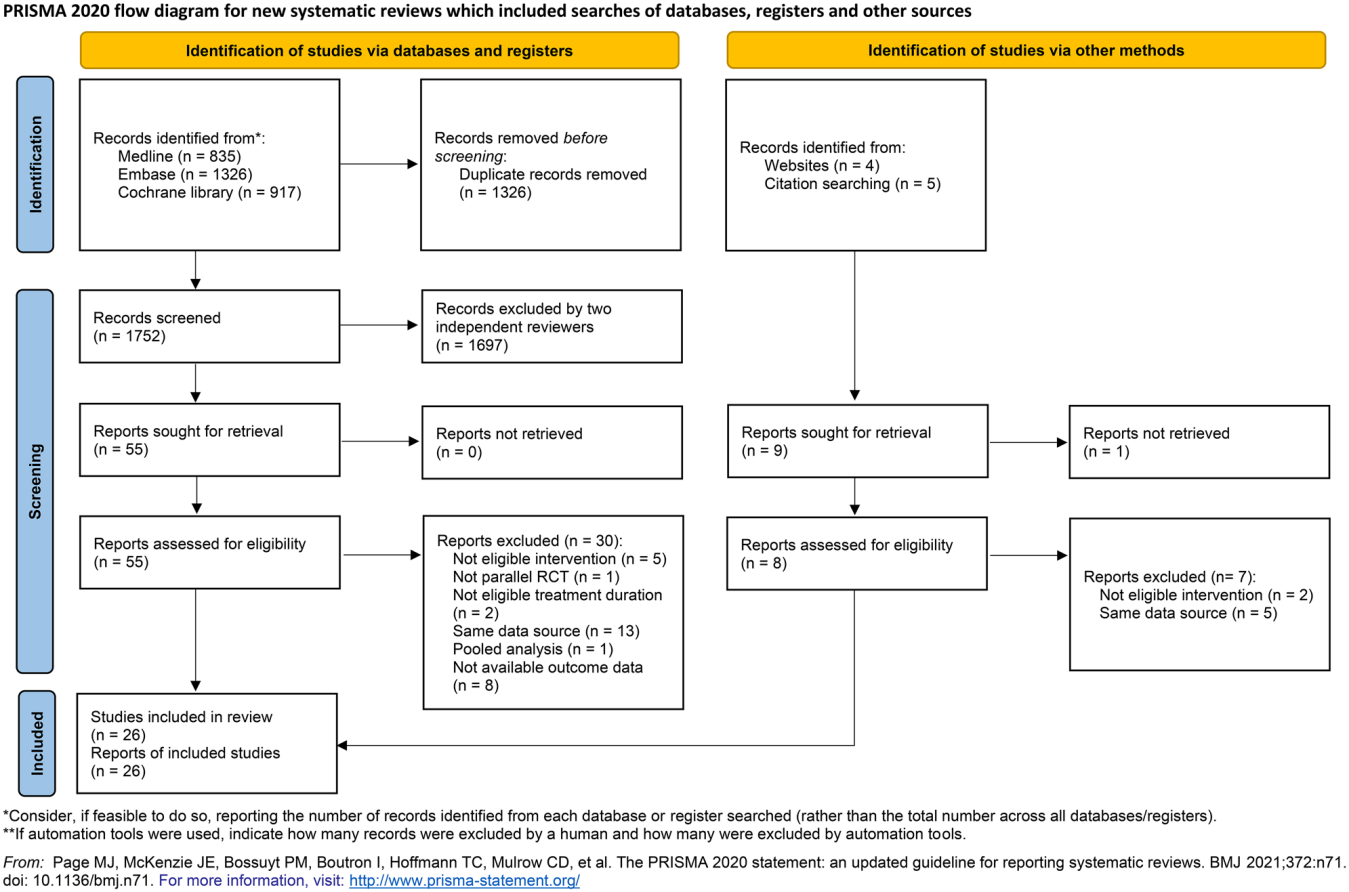
PRISMA flow chart for study inclusion in the systematic review and network meta-analysis.

**Figure 2:**
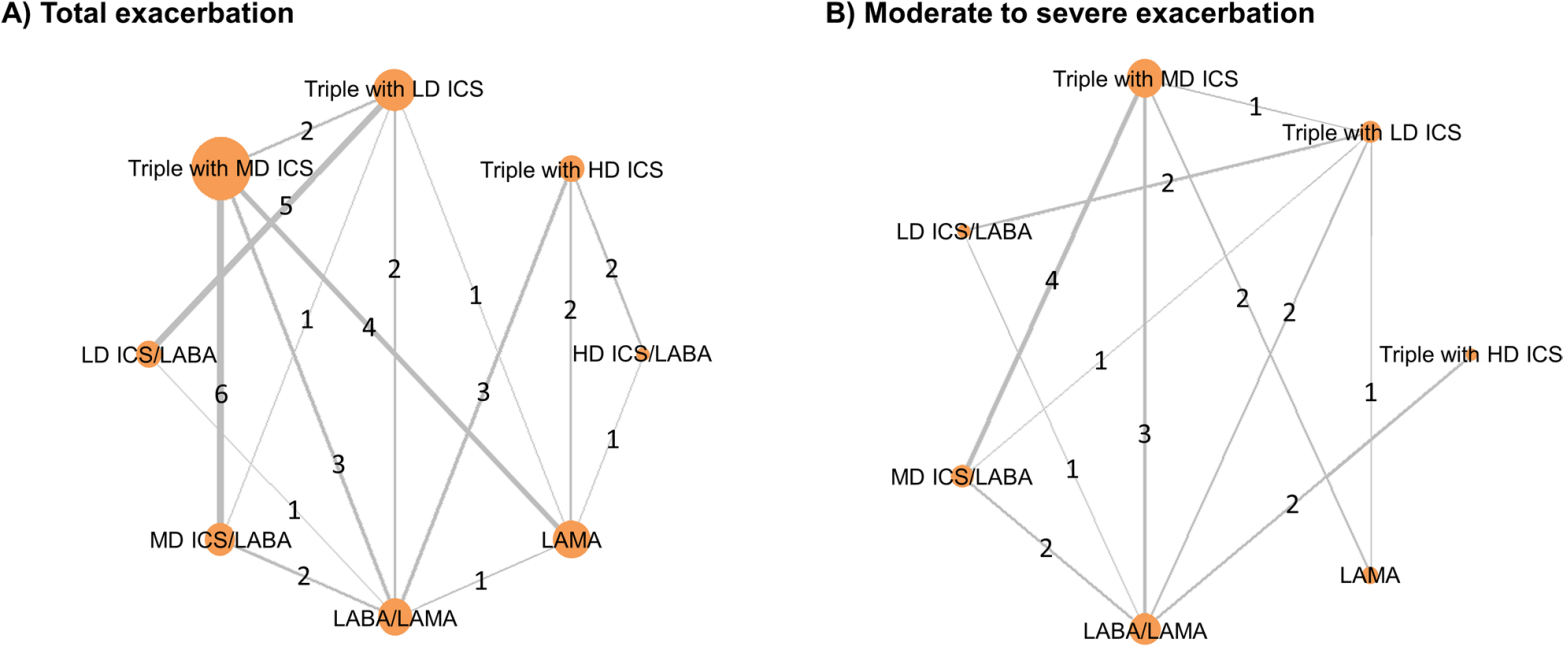
Network geometry of the 26 eligible randomized controlled trials.

We identified a total of 2871 records from the pre-specified databases (Fig. 1). After the removal of 1237 duplicate records, 1634 records were screened to find 55 relevant articles or abstracts for retrieval. Among the nine records identified from other sources, one report was not accessible and seven reports did not meet the eligibility criteria. After the full-text review, 26 studies met the eligibility criteria of the present SR. Supplementary information 1 includes a summary of the excluded references and the major reasons for the exclusion. A network geometry of the included RCTs is graphically described for total and moderate-to-severe exacerbation in Fig. 2. Direct head-to-head comparison between triple therapies with different ICS doses was found in four studies, all of which compared MD-ICS and LD-ICS.

### Study characteristics

Detailed information on the 25 published and one unpublished eligible RCTs conducted between 2007 and 2022 and the 41,366 participants included in those RCTs are summarized in Table 1. After the full-text review, 26 studies from 23 references^17–39^ met the eligibility criteria. A direct head-to-head comparison between triple therapies with different ICS doses was found in four studies, all of which compared MD-ICS and LD-ICS^17,23,24^. The mean age was 64.7 years, the proportion of men was 68.4%, and the proportion of current smokers was approximately 40.7%. In 14 RCTs with information on ethnicity, the predominant ethnicities were white/Caucasian and Asian. Patients with moderate-to-very severe COPD were included and their mean post-bronchodilator FEV_1_% was 43.8%. The distribution of the Global Initiative for Chronic Obstructive Lung Disease (GOLD) grades was as follows: grades 3–4, 40,917 (37.9%) patients; grades 2–4, 39,403 (36.5%) patients; and grades 2–3, 27,536 (25.5%) patients. HD-ICS was used in five RCTs^18–22^, MD-ICS in 15 RCTs^17,23–34^, and LD-ICS in 10 RCTs^17,23,24,35–39^.

**Table 1:** Baseline characteristics of the 26 included studies.

Published year	Author (study name)	Study ID	Number of patients	Age	Male, %	Current smoker, %	Ethnicity	Post-BDR FEV1, %	GOLD stage	Previous exacerbation, %	Symptom score	Intervention (ICS/LABA/LAMA)	ICS dose	Inhalation device*	Study duration
2007	Aaron et al.	ISRCTN29870041	449	67.7	56.4	27.9	White: 98.2%	41.8	2–4	100	–	FP/SAL/TIO	High	DPI	52 weeks
2007	Cazzola et al.	–	90	65.8	88.9	25.7	–	38.1	3–4	–	–	FP/SAL/TIO	High	DPI	12 weeks
2009	Welte et al.	NCT00496470	660	62.5	75.2	43.9	–	37.9	3–4	100	–	BUD/FOR/TIO	Medium	DPI	12 weeks
2012	Hanania et al.	NCT00784550	342	61.2	46.5	58	White:96%, African American: 4%	56.7	2–3	29	mMRC ≥ 2	FP/SAL/TIO	Medium	DPI	24 weeks
2012	Jung et al.	NCT01610037	455	67.4	98	–	–	–	2–4	–	–	FP/SAL/TIO	Medium	DPI	24 weeks
2014	Magnussen et al.	NCT00975195	2049	63.8	82.5	–	–	–	3–4	100	–	FP/SAL/TIO	High	DPI + MDI	52 weeks
2015	Frith et al.	NCT01513460	772	68	64.4	35.8	Caucasian: 95.7%	57.2	2–3	34.9	–	1) FP/SAL/GLY 2) FP/SAL/TIO	High	DPI	12 weeks
2015	Siler et al. (Study 1)	NCT01957163	619	64.5	66	42.3	–	45.4	2–4	23.9	mMRC ≥ 2	FF/VIL/UME	Low	DPI	12 weeks
2015	Siler et al. (Study 2)	NCT02119286	619	62.8	62.8	57.5	–	47.3	2–4	21.4	mMRC ≥ 2	FF/VIL/UME	Low	DPI	12 weeks
2016	Lee et al.	NCT01397890	577	66.8	95.7	–	Asian: 100%	36.4	3–4	100	–	BUD/FOR/TIO	Medium	DPI	12 weeks
2016	Siler et al. (Study 1)	NCT01772134	614	63.2	65.5	55	–	47.1	2–4	–	mMRC ≥ 2	FP/SAL/UME	Medium	DPI	12 weeks
2016	Siler et al. (Study 2)	NCT01772147	606	65.4	62.5	37.8	–	45.3	2–4	–	mMRC ≥ 2	FP/SAL/UME	Medium	DPI	12 weeks
2016	Singh et al	NCT01917331	1367	63.6	75.5	47	White: > 99%	36.6	3–4	100	CAT ≥ 10	BDP/FOR/GLY	Medium	MDI^a^	52 weeks
2017	Lipson et al.	NCT02345161	1810	64	74	44	White:85%	45.3	2–4	65.5	CAT ≥ 10	FF/VIL/UME	Low	DPI	24 weeks
2017	Vestbo et al.	NCT01911364	2690	63.1	76	48.3	White:99%	36.6	3–4	100	CAT ≥ 10	1) BDP/FOR/GLY 2) BDP/FOR/TIO	Medium	MDI^a^	52 weeks
2018	Chapman et al.	NCT02603393	1053	65.3	70.6		–	56.6	2–3	34.1	–	FP/SAL/TIO	High	DPI	26 weeks
2018	Ferguson et al.	NCT02497001	1896	65.2	71.2	39.6	White: 50.1%, Asian: 45.3%	50.3	2–3	–	mMRC ≥ 2	BUD/FOR/GLY	Medium	MDI	24 weeks
2018	Lipson et al.	NCT02164513	10,355	65.3	66.3	34.7	White:78%, Asian: 16%	45.5	3–4	100	CAT ≥ 10	FF/VIL/UME	Low	DPI	52 weeks
2018	Papi et al.	NCT02579850	1532	64.5	72	44.5	–	36.4	3–4	100	CAT ≥ 10	BDP/FOR/GLY	Medium	MDI^a^	52 weeks
2018	Zhao et al.	ChiCTR1800017584	180	55.8	70	–	–	–	2–3	–	–	BUD/FOR/TIO	Low	DPI	24 weeks
2020	Ferguson et al. (Study 1)	NCT03478683	728	65.2	52.7	48.6	White:90.3%, Black: 9.2%	42.4	2–4	52.6	CAT ≥ 10	1) FF/VIL/UME 2) BUD/FOR/TIO	1) Low 2) Medium	DPI + MDI	12 weeks
2020	Ferguson et al. (Study 2)	NCT03478696	732	65.3	51	49.2	White:89.7%, Black 9.0%, Asian 1.0%	42.1	2–4	52.9	CAT ≥ 10	1) FF/VIL/UME 2) BUD/FOR/TIO	1) Low 2) Medium	DPI + MDI	12 weeks
2020	Rabe et al.	NCT02465567	8509	64.7	59.7	41.1	Hispanic or Latino: 19.0%, No Hispanic or Latino: 78.8%	43.4	2–4	100	CAT ≥ 10	BUD/FOR/GLY	1) Low 2) Medium	MDI	52 weeks
2021	Bansal et al.	NCT03474081	799	66.2	67.9	47.6	White:97.3%, African: 2.8%	50	2–4	63	CAT ≥ 10	FF/VIL/UME	Low	DPI	12 weeks
2021	Zheng et al.	NCT03197818	706	66	95.3	24.7	Asian:100%	34.5	3–4	100		BDP/FOR/GLY	Medium	DPI + MDI^a^	24 weeks
–	(TRISTAR study)	NCT02467452	1157	64	75.5	–	–	–	3–4	100	mMRC ≥ 2	1) FF/VIL/TIO 2) BDP/FOR/GLY	1) Low 2) Medium	MDI^a^	26 weeks

ID: identifier; BDR: bronchodilator; CAT: COPD assessment test; mMCRC: modified Medical Research Council; FEV_1_: forced expiratory volume in one second; GOLD : Global Initiative for Chronic Obstructive Lung Disease; ICS: inhaled corticosteroid; LABA: long-acting beta-agonist; LAMA: long-acting muscarinic antagonist; DPI: dry powder inhaler; MDI: metered dose inhaler; BDP: Beclomethasone; BUD: Budesonide; FF: Fluticasone furoate; FOR: Formoterol; FP: Fluticasone propionate; GLY: Glycopyrrolate; IND: Indacaterol; SAL: Salmeterol; TIO: Tiotropium dry powder inhaler; UME: Umeclidinium; VIL: Vilanterol.

We defined the dose levels as follows: low-dose-ICS, 100–250 mg, medium-dose-ICS, 251–500 mg, and high-dose-ICS, > 500 mg of fluticasone propionate or equivalent.

^*^Inhaler devices were summarized based on how the triple therapy was provided.

^a^Extrafine particle inhaled therapy.

### Risk of bias (ROB) within and across studies

As per the assessment of ROB within studies, the quality of the included RCTs was considered to be generally acceptable for the NMA (Supplementary information 2). Detailed information on ROB assessment is summarized in Supplementary information 3. In the assessment of ROB across studies, we could not find either significant publication bias or selective reporting bias (Supplementary information 4).

### Certainty of evidence

The certainty of evidence is described in Supplementary information 5.

### Acute exacerbations and mortality

The risk of total exacerbation was compared among inhaled therapies in 23 RCTs with 39,682 participants (Table 2). Among the drug classes, the highest surface under the cumulative ranking curve (SUCRA) was observed for triple therapy with HD-ICS. Triple therapy with HD-ICS exhibited a significantly lower risk of total exacerbation compared with LABA/LAMA, MD-ICS/LABA, LD-ICS/LABA, and LAMA. Triple therapy with MD-ICS exhibited a significantly lower risk of total exacerbation than MD-ICS/LABA. Triple therapy with LD-ICS exhibited a significantly lower risk of total exacerbation than MD-ICS/LABA and LD-ICS/LABA. In the pre-specified sensitivity analyses, triple therapy with HD-ICS showed significant superiority in reducing total exacerbation compared to triple therapy with LD- or MD-ICS in the subgroups with a study duration of ≥ 48 weeks (HD-ICS *vs.* LD-ICS, OR = 0.66 [95% credible interval (CrI) = 0.52–0.94], low certainty of evidence; HD-ICS *vs.* MD-ICS, OR = 0.66 [95% CrI = 0.51–0.94], low certainty of evidence) (Supplementary information 6).

**Table 2:** Acute exacerbation and mortality associated with different inhaled therapies.

	Triple therapy with HD-ICS	Triple therapy with MD-ICS	Triple therapy with LD-ICS	LABA/LAMA	HD-ICS/LABA	MD-ICS/LABA	LD-ICS/LABA	LAMA
**Total exacerbation (23 studies, 39,682 patients)**								
Rank	1	3	2	5	4	8	6	7
SUCRA, %	93.49	69.75	76.77	40.15	50.37	13.37	28.89	27.22
**NMA estimate OR (95% CrI)**								
Triple therapy with HD-ICS	1							
Triple therapy with MD-ICS	0.81 (0.61–1.14)	1						
Triple therapy with LD-ICS	0.84 (0.62–1.22)	1.04 (0.84–1.31)	1					
LABA/LAMA	0.7 (0.55–0.93)*	0.86 (0.7–1.05)	0.83 (0.65–1.03)	1				
HD-ICS/LABA	0.75 (0.43–1.29)	0.92 (0.49–1.69)	0.89 (0.45–1.63)	1.07 (0.58–1.92)	1			
MD-ICS/LABA	0.61 (0.43–0.85)*	0.75 (0.6–0.89)*	0.72 (0.53–0.92)*	0.87 (0.67–1.08)	0.81 (0.43–1.54)	1		
LD-ICS/LABA	0.66 (0.45–0.98)*	0.81 (0.58–1.09)	0.78 (0.59–0.98)*	0.94 (0.69–1.24)	0.88 (0.46–1.73)	1.09 (0.78–1.52)	1	
LAMA	0.65 (0.48–0.93)*	0.81 (0.64–1.01)	0.77 (0.58–1.02)	0.93 (0.71–1.22)	0.88 (0.47–1.68)	1.08 (0.81–1.48)	0.99 (0.7–1.44)	1
**Moderate-to-severe exacerbation (12 studies, 33,545 patients)**								
Rank	1	3	2	4		7	5	6
SUCRA, %	97.20	70.23	72.56	36.55		14.26	35.18	24.05
**NMA estimate OR (95% CrI)**								
Triple therapy with HD-ICS	1							
Triple therapy with MD-ICS	0.73 (0.52–1.09)	1						
Triple therapy with LD-ICS	0.73 (0.53–1.13)	1.01 (0.79–1.33)	1					
LABA/LAMA	0.63 (0.48–0.88)*	0.87 (0.7–1.07)	0.86 (0.67–1.07)	1				
HD-ICS/LABA	–	–	–	–	–			
MD-ICS/LABA	0.57 (0.39–0.85)*	0.79 (0.63–0.95)*	0.78 (0.57–0.99)*	0.9 (0.7–1.12)	–	1		
LD-ICS/LABA	0.63 (0.42–0.98)*	0.87 (0.61–1.19)	0.86 (0.63–1.08)	0.99 (0.72–1.32)	–	1.1 (0.78–1.57)	1	
LAMA	0.59 (0.38–0.96)*	0.81 (0.6–1.08)	0.8 (0.56–1.12)	0.93 (0.66–1.31)	–	1.03 (0.73–1.48)	0.94 (0.62–1.44)	1
**All–cause mortality (24 studies, 41,004 patients)**								
Rank	6	1	3	8	7	2	5	4
SUCRA, %	36.89	86.95	65.2	27.44	27.57	74.12	37.68	44.15
**NMA estimate OR (95% CrI)**								
Triple therapy with HD-ICS	1							
Triple therapy with MD-ICS	1.53 (0.86–2.88)	1						
Triple therapy with LD-ICS	1.26 (0.71–2.39)	0.82 (0.53–1.26)	1					
LABA/LAMA	0.96 (0.6–1.6)	0.62 (0.42–0.92)*	0.76 (0.53–1.09)	1				
HD-ICS/LABA	0.41 (0.01–11.61)	0.27 (0.01–7.69)	0.32 (0.01–9.64)	0.42 (0.01–13.03)	1			
MD-ICS/LABA	1.38 (0.73–2.77)	0.9 (0.59–1.39)	1.1 (0.68–1.79)	1.45 (0.93–2.26)	3.41 (0.11–123.14)	1		
LD-ICS/LABA	1.02 (0.5–1.91)	0.67 (0.35–1.08)	0.82 (0.46–1.18)	1.08 (0.6–1.59)	2.46 (0.08–87.96)	0.74 (0.37–1.26)	1	
LAMA	1.07 (0.52–2.32)	0.7 (0.42–1.2)	0.85 (0.46–1.62)	1.13 (0.62–2.09)	2.69 (0.09–88.26)	0.78 (0.41–1.53)	1.05 (0.54–2.38)	1

CrI: credible interval; HD: high-dose; ICS: inhaled corticosteroid; LABA: long-acting beta-agonist; LAMA: long-acting muscarinic antagonist; LD: low-dose; MD: medium-dose; NMA: network meta-analysis; OR: odds ratio; SUCRA: surface under the cumulative ranking curve.

Median OR with 95% CrI was calculated as a row to column ratio. If the OR is significantly lower than 1, the drug in the left row is considered to be more beneficial than the other drug in the upper column.

*Indicates that the posterior probability is either less than 0.025 or more than 0.975, which is considered statistically significant.

The risk of moderate-to-severe exacerbation was compared among the inhaled therapies in 12 RCTs with 33,545 participants (Table 2). Triple therapy with HD-ICS exhibited the highest SUCRA among the drug classes, and conferred a significantly lower risk of moderate-to-severe exacerbation than LABA/LAMA, MD-ICS/LABA, LD-ICS/LABA, and LAMA. Triple therapy with MD-ICS exhibited a significantly lower risk of moderate-to-severe exacerbation than MD-ICS/LABA. Triple therapy with LD-ICS exhibited a significantly lower risk of moderate-to-severe exacerbation than MD-ICS/LABA. In the pre-specified sensitivity analyses, triple therapy with HD-ICS was also superior to triple therapy with MD-ICS in subgroups with FEV_1_ < 65% (OR = 0.6 [95% CrI = 0.37–0.98], low certainty of evidence) or at least 1 exacerbation event in the past year (OR = 0.6 [95% CrI = 0.36–0.999], very low certainty of evidence) (Supplementary information 7).

The risk of all-cause mortality was compared among different inhaled therapies in 24 RCTs with 41,004 participants (Table 2). Triple therapy with MD-ICS exhibited the highest SUCRA. Triple therapy with MD-ICS was associated with a significantly lower risk of all-cause mortality compared to LABA/LAMA (OR = 0.62 [95% CrI = 0.42–0.92], high certainty of evidence). There was no significant finding in the pre-specified sensitivity analyses for all-cause mortality (Supplementary information 8).

### Lung function and symptoms

The mean change in trough FEV_1_ was compared among the inhaled therapies in 17 RCTs with 24,823 participants (Table 3). Triple therapy with LD-ICS exhibited the highest SUCRA among the drug classes. Triple therapy with MD-ICS was associated with a significantly improved FEV_1_ compared to HD-ICS/LABA, MD-ICS/LABA, LD-ICS/LABA, and LAMA. Triple therapy with LD-ICS led to a significantly improved FEV_1_ compared to that with LABA/LAMA, HD-ICS/LABA, MD-ICS/LABA, LD-ICS/LABA, and LAMA. In the pre-specified sensitivity analyses, triple therapy with HD-ICS was superior to LD-ICS/LABA in terms of improving trough FEV_1_ in the subgroups with study duration < 24 or < 48 weeks (Supplementary information 9).

**Table 3:** Change in trough FEV_1_ or SGRQ score with different inhaled therapies.

	Triple therapy with HD-ICS	Triple therapy with MD-ICS	Triple therapy with LD-ICS	LABA/LAMA	HD-ICS/LABA	MD-ICS/LABA	LD-ICS/LABA	LAMA
**Change of trough FEV**_**1**_**, ml (17 studies, 24,823 patients)**								
Rank	3	2	1	4	7	5	8	6
SUCRA, %	65.34	82.01	98.55	60.37	27.62	31.86	5.92	28.32
**NMA estimate mean difference (95% CrI)**								
Triple therapy with HD-ICS	1							
Triple therapy with MD-ICS	− 25.6 (− 93.9 to 43.1)	1						
Triple therapy with LD-ICS	− 52.9 (− 123.2 to 17.4)	− 27.5 (− 54.6 to 0.2)	1					
LABA/LAMA	6.4 (− 71.1 to 83)	31.8 (− 4.8 to 70.2)	59.5 (22.8 to 96.2)*	1				
HD-ICS/LABA	46.2 (− 15.3 to 106.3)	71.5 (1.9–140.6)*	99.3 (27.8–168.6)*	39.9 (− 37.7 to 115.3)	1			
MD-ICS/LABA	41.1 (− 33.8 to 116.6)	66.6 (34.4–98.8)*	94.1 (53.5–134.6)*	34.8 (− 8.8 to 77.5)	− 4.8 (− 80 to 70.7)	1		
LD-ICS/LABA	73.3 (− 0.8 to 149.6)	98.9 (63.5–138.3)*	126.6 (100.6–155.5)*	66.9 (27.4–109.6)*	27.3 (− 46.3 to 105.2)	32.5 (− 12.9 to 81)	1	
LAMA	44.7 (− 15.2 to 105.1)	70.3 (37.8–102.8)*	97.8 (60–134.8)*	38.5 (− 8.9 to 84.8)	− 1.5 (− 62.2 to 60.5)	3.7 (− 41.7 to 49.7)	− 28.7 (− 76.3 to 14.6)	1
**Change of SGRQ score (19 studies, 30,404 patients)**								
Rank	3	2	1	6	7	5	4	8
SUCRA, %	77.76	86.81	86.85	36.17	13.37	41.51	49.45	8.08
**NMA estimate mean difference (95% CrI)**								
Triple therapy with HD-ICS	1							
Triple therapy with MD-ICS	0.3 (− 1.6 to 2.1)	1						
Triple therapy with LD-ICS	0.3 (− 1.6 to 2)	0 (− 0.9 to 0.9)	1					
LABA/LAMA	− 1.3 (− 2.9 to 0.1)	− 1.6 (− 2.7 to − 0.5)*	− 1.6 (− 2.6 to − 0.5)*	1				
HD-ICS/LABA	− 2.7 (− 4.9 to − 0.6)*	− 3 (− 5.8 to − 0.2)*	− 3 (− 5.8 to − 0.2)*	− 1.4 (− 4 to 1.2)	1			
MD-ICS/LABA	− 1.2 (− 3.2 to 0.6)	− 1.5 (− 2.5 to − 0.6)*	− 1.5 (− 2.7 to − 0.4)*	0.1 (− 1.1 to 1.3)	1.5 (− 1.4 to 4.3)	1		
LD-ICS/LABA	− 1 (− 2.8 to 1.2)	− 1.3 (− 2.4 to 0.2)	− 1.3 (− 2 to − 0.03)*	0.4 (− 0.8 to 1.9)	1.7 (− 1 to 4.9)	0.2 (− 1 to 2)	1	
LAMA	− 2.9 (− 5.3 to − 0.6)*	− 3.1 (− 4.8 to − 1.6)*	− 3.1 (− 4.8 to − 1.6)*	− 1.5 (− 3.4 to 0.2)	− 0.1 (− 3.4 to 3)	− 1.7 (− 3.4 to 0.1)	− 1.9 (− 4 to − 0.2)*	1

CrI: credible interval; FEV_1_: forced expiratory volume in one second; HD: high-dose; ICS: inhaled corticosteroid; LABA: long-acting beta-agonist; LAMA: long-acting muscarinic antagonist; LD: low-dose; MD: medium-dose; NMA: network meta-analysis; SGRQ: St. George's respiratory questionnaire; SUCRA: surface under the cumulative ranking curve.

Mean difference with 95% CrI was calculated by subtracting row (left) from column (upper). If the mean difference is significantly lower than 0, the drug in the left row is considered to be more beneficial than the other drug in the upper column.

*Indicates that the posterior probability is either less than 0.025 or more than 0.975, which is considered statistically significant.

The mean change in the St. George's Respiratory Questionnaire (SGRQ) score was compared among the inhaled therapies in 19 RCTs with 30,404 participants (Table 3). Triple therapy with LD-ICS exhibited the highest SUCRA among the drug classes. Triple therapy with HD-ICS showed a significantly decreased SGRQ compared to HD-ICS/LABA and LAMA. Triple therapy with MD-ICS exhibited a significantly decreased SGRQ compared to LABA/LAMA, HD-ICS/LABA, MD-ICS/LABA, and LAMA. Triple therapy with LD-ICS showed a significantly decreased SGRQ compared to LABA/LAMA, HD-ICS/LABA, MD-ICS/LABA, LD-ICS/LABA, and LAMA. In the pre-specified sensitivity analyses, triple therapy with MD-ICS was superior to LD-ICS/LABA in terms of improving SGRQ scores in the subgroups with FEV_1_ < 65%, at least one exacerbation history in the past year, or more symptoms (COPD Assessment Test (CAT) ≥ 10 or modified Medical Research Council (mMRC) ≥ 2) (Supplementary information  10).

### Adverse events

The risk of serious adverse events was compared among different inhaled therapies in 25 RCTs with 41,623 participants (Table 4). Triple therapy with MD-ICS was associated with a lower risk of serious adverse events compared to LABA/LAMA or MD-ICS/LABA. There was no significant finding in the pre-specified sensitivity analyses for serious adverse events (Supplementary information 11).

**Table 4:** Adverse events associated with different inhaled therapies.

	Triple therapy with HD-ICS	Triple therapy with MD-ICS	Triple therapy with LD-ICS	LABA/LAMA	HD-ICS/LABA	MD-ICS/LABA	LD-ICS/LABA	LAMA
**Serious adverse event (25 studies, 41,623 patients)**								
Rank	5	1	4	7	2	6	3	8
SUCRA, %	42.21	86.62	44.04	34.88	70.05	39.2	51.86	31.14
**NMA estimate OR (95% CrI)**								
Triple therapy with HD-ICS	1							
Triple therapy with MD-ICS	1.17 (0.89–1.65)	1						
Triple therapy with LD-ICS	1.01 (0.75–1.37)	0.86 (0.7–1.02)	1					
LABA/LAMA	0.98 (0.77–1.27)	0.84 (0.68–0.98)*	0.97 (0.82–1.17)	1				
HD-ICS/LABA	1.24 (0.64–2.45)	1.06 (0.51–2.22)	1.23 (0.6–2.6)	1.26 (0.62–2.63)	1			
MD-ICS/LABA	1 (0.73–1.37)	0.85 (0.69–0.99)*	0.98 (0.79–1.21)	1.01 (0.83–1.23)	0.8 (0.37–1.65)	1		
LD-ICS/LABA	1.03 (0.71–1.4)	0.89 (0.63–1.09)	1.03 (0.79–1.22)	1.05 (0.79–1.28)	0.83 (0.38–1.72)	1.04 (0.76–1.32)	1	
LAMA	0.96 (0.67–1.41)	0.81 (0.64–1.04)	0.95 (0.71–1.29)	0.97 (0.73–1.31)	0.77 (0.36–1.63)	0.96 (0.72–1.32)	0.93 (0.68–1.38)	1
**Serious cardiac adverse event (23 studies, 40,552 patients)**								
Rank	5	2	3	7	4	1	8	6
SUCRA, %	48.12	71.12	63.29	22.59	60.46	73.2	17.39	43.82
**NMA estimate OR (95% CrI)**								
Triple therapy with HD-ICS	1							
Triple therapy with MD-ICS	1.20 (0.64–2.23)	1						
Triple therapy with LD-ICS	1.13 (0.59–2.20)	0.94 (0.64–1.38)	1					
LABA/LAMA	0.83 (0.48–1.41)	0.69 (0.49–0.96)*	0.73 (0.49–1.07)	1				
HD-ICS/LABA	1.21 (0.35–5.18)	1.01 (0.25–4.61)	1.08 (0.25–5.17)	1.45 (0.38–6.42)	1			
MD-ICS/LABA	1.24 (0.60–2.42)	1.04 (0.67–1.52)	1.1 (0.67–1.73)	1.5 (0.94–2.3)	1.01 (0.21–4.2)	1		
LD-ICS/LABA	0.75 (0.35–1.61)	0.62 (0.36–1.1)	0.66 (0.42–1.03)	0.9 (0.54–1.55)	0.61 (0.13–2.63)	0.6 (0.33–1.14)	1	
LAMA	0.97 (0.43–2.14)	0.81 (0.44–1.44)	0.86 (0.43–1.7)	1.17 (0.59–2.21)	0.78 (0.17–3.53)	0.79 (0.38–1.62)	1.29 (0.58–2.85)	1
**Pneumonia (25 studies, 41,713 patients)**								
Rank	3	5	6	1	7	8	4	2
SUCRA, %	65.94	37.14	32.24	85.01	28.74	23.31	54.05	73.57
**NMA estimate OR (95% CrI)**								
Triple therapy with HD-ICS	1							
Triple therapy with MD-ICS	0.78 (0.45–1.50)	1						
Triple therapy with LD-ICS	0.77 (0.45–1.45)	0.98 (0.69–1.35)	1					
LABA/LAMA	1.14 (0.73–1.91)	1.47 (1.004–2.01)*	1.50 (1.06–2.04)*	1				
HD-ICS/LABA	0.48 (0.04–5.15)	0.61 (0.04–6.8)	0.63 (0.05–7.08)	0.42 (0.03–4.75)	1			
MD-ICS/LABA	0.70 (0.41–1.42)	0.91 (0.66–1.26)	0.93 (0.65–1.41)	0.62 (0.44–0.96)*	1.48 (0.13–20.92)	1		
LD-ICS/LABA	0.88 (0.50–1.85)	1.12 (0.72–1.82)	1.13 (0.83–1.76)	0.76 (0.53–1.25)	1.84 (0.17–25.88)	1.24 (0.76–2.05)	1	
LAMA	1.07 (0.52–2.35)	1.35 (0.81–2.27)	1.39 (0.78–2.53)	0.93 (0.52–1.74)	2.21 (0.19–31.8)	1.49 (0.82–2.7)	1.22 (0.61–2.32)	1

CrI: credible interval; HD: high-dose; ICS: inhaled corticosteroid; LABA: long-acting beta-agonist; LAMA: long-acting muscarinic antagonist; LD: low-dose; MD: medium-dose; NMA: network meta-analysis; OR: odds ratio; SUCRA: surface under the cumulative ranking curve.

Median OR with 95% CrI was calculated as a row to column ratio. If the OR is significantly lower than 1, the drug in the left row is considered to be more beneficial than the other drug in the upper column.

*Indicates that the posterior probability is either less than 0.025 or more than 0.975, which is considered statistically significant.

The risk of serious cardiac adverse events was compared among different inhaled therapies in 23 RCTs with 40,552 participants (Table 4). Triple therapy with MD-ICS was associated with a lower risk of serious cardiac adverse events compared to LABA/LAMA. We incidentally found that MD-ICS/LABA was associated with a lower risk of serious cardiac adverse events compared to LABA/LAMA. There was no significant finding in the sensitivity analyses for serious cardiac adverse events (Supplementary information 12).

The risk of pneumonia was compared among different inhaled therapies in 25 RCTs with 41,713 participants (Table 4). The risk of pneumonia was higher with triple therapy with MD-ICS (OR = 1.47 [95% CrI = 1.004–2.01], moderate certainty of evidence) or LD-ICS (OR = 1.50 [95% CrI = 1.06–2.04], high certainty of evidence) than with LABA/LAMA. There was no significant finding in the pre-specified sensitivity analyses for pneumonia (Supplementary information 13).

### Consistency assumption

The consistency assumption was satisfied between the estimated effect size in the paired meta-analysis via direct comparisons and the estimated effect size by Bayesian NMA via indirect comparisons. Detailed information is summarized in Supplementary information 14.

## Discussion

Our study investigated the differences in the efficacy and safety of triple therapy with varying ICS doses used for > 12 weeks in patients with COPD. Although there was no significant difference in the analysis including all eligible studies, triple therapy with HD-ICS showed superiority in reducing total or moderate-to-severe exacerbation compared to triple therapy with LD- or MD-ICS in sensitivity analyses, with a low certainty of evidence. Triple therapy with MD-ICS was associated with the lowest risk of all-cause mortality and serious adverse events, and triple therapy using LD-ICS showed the highest efficacy in improving SGRQ scores and FEV_1_ based on SUCRA, although there were no significant differences among the triple therapies in this regard. Pneumonia risk was comparable among the triple therapies with different ICS doses.

The optimal ICS dose for patients with COPD has been debated upon by various researchers. Our study provides low certainty of evidence supporting the contention that triple therapy with HD-ICS is a better option for reducing exacerbation than triple therapies with MD- or LD-ICS. In patients with difficult-to-treat or severe asthma, high-dose ICS is often required to control symptoms or reduce exacerbation^40^. In chronic airway diseases, neutrophilic airway inflammation is considered to be an important mechanism underlying a reduced response to ICS^41^. COPD is characterized by chronic airway inflammation caused by an increase in the number and activation of neutrophils^42^. Neutrophilic airway inflammation can reduce the response to ICS through the dysregulation of the glucocorticoid receptor and impairment of mitogen-activated protein kinase phosphatase 1 function^43,44^. Considering that neutrophilic airway inflammation is related to the severity of COPD^45,46^, a higher dose of ICS is likely to be more effective because a majority of patients with COPD who need triple therapy would have elevated airway inflammation. In addition, considering that increased airway inflammation underlies acute exacerbation of chronic obstructive pulmonary disease (AE-COPD)^47^, decreased activation of inflammatory cells and pro-inflammatory cytokines would be one of the plausible mechanisms for the reduced exacerbation observed with HD-ICS (triple therapy) in the COPD subgroups^48^. Synergistic anti-inflammatory effects of ICS, LABA, and LAMA may further reduce acute exacerbation in triple therapy^49^. It has been reported that a ceiling efficacy (maximal efficacy) can be achieved with LD-ICS, and that HD-ICS may not show additional benefit. However, the synergism of triple combination therapy may attenuate the ceiling effect observed with HD-ICS^14,15^. LABA and LAMA have a synergistic effect on bronchodilation and act by inhibiting acetylcholine release from airway epithelium; they also exert anti-inflammatory action through the inactivation of inflammatory signaling pathways^50^. Through the complementary/additive effects of LABA and ICS, the anti-inflammatory and anti-remodeling activity of ICS can be enhanced, even when there is no further benefit from increasing the ICS dose^51,52^. Synergistic interactions between ICS and LAMA lead to increased cAMP, which further leads to bronchorelaxation and decreases airway inflammation^49^. Recently, triple therapy also exhibited a synergistic effect with reference to small airway relaxation compared to ICS/LABA^53^. In summary, additional anti-inflammatory activity due to synergistic effects of the components of triple therapy with HD-ICS may explain the reduced exacerbation observed in this study. However, dose-dependence of the ICS responses in triple therapy need further clarification.

Although there was no significant difference in the associated mortality risk among the triple therapies with different ICS doses, only triple therapy with MD-ICS showed superiority in reducing all-cause mortality compared to LABA/LAMA. In addition, only triple therapy with MD-ICS was able to show a higher reduction in serious adverse events/serious cardiac adverse events compared to LABA/LAMA. Cardiovascular events are among the major causes of death in COPD^54^. Previous observational studies showed that ICS may exert a cardioprotective effect^55^. Our results suggest that physicians should consider triple therapy with MD-ICS in patients with cardiovascular comorbidity or history of serious adverse events, especially among those treated with LABA/LAMA.

Various studies have reported on the improvement of lung function or health-related quality of life by ICS. Although the use of ICS led to lung function improvement in the SUMMIT^56^ and TORCH^57^ trials and SGRQ improvement in the IMPACT trial^37^, there is no consensus on whether the improvement was clinically meaningful. A recent paired meta-analysis revealed that triple therapy improved both FEV_1_ and SGRQ compared to LABA/LAMA with statistical and clinical significance^58^. In our study, there was no significant difference in the improvement of lung function and SGRQ according to the ICS dose among the triple therapies. However, triple therapy with LD-ICS exhibited the highest SUCRA and significantly improved FEV_1_ and SGRQ compared to LABA/LAMA, ICS/LABA, or LAMA. Therefore, adding LD-ICS to LABA/LAMA may have the benefit of improving FEV_1_ or SGRQ in patients with COPD who need triple therapy.

Many studies have evaluated the dose-dependence of ICS-related adverse events in COPD. A higher dose of ICS conferred a significantly higher risk of hospitalization for pneumonia^59^. In addition, a higher dose of ICS was related to tuberculosis^60^, diabetes^61^, bone fracture^62^, and cataract^63^. However, the risk of adverse events according to ICS dose among triple therapies has not been well evaluated. In our study, escalation to triple therapy with HD-ICS did not increase the risk of pneumonia. Interestingly, we found that triple therapy with MD-ICS significantly increased the risk of pneumonia but reduced the incidence of serious cardiac adverse events compared to LABA/LAMA. Overall, there were significantly fewer serious adverse events with triple therapy with MD-ICS than with LABA/LAMA. This result can be explained by the findings from a previous investigation in which most ICS-related pneumonias were reported to be of low severity^64^.

The strengths of the present study are as follows. First, to our knowledge, this is the first SR and NMA to compare triple therapies according to ICS doses in patients with stable COPD. Second, we performed a novel and extensive SR and NMA by reviewing 2,880 articles including unpublished data and the latest clinical trials. Third, we used Bayesian methods for performing pertinent comparisons of rare events such as mortality or serious cardiac adverse events^65^. Bayesian NMA is a useful method to estimate the comparative efficacy of different treatments when head-to-head RCTs are insufficient. Bayesian NMA provides the probability of the best treatment or SUCRA among different treatments for each outcome. This approach can be more useful for clinicians in decision-making situations than a p-value^66^.

There were several limitations to our study. First, clinical heterogeneity regarding symptom severity, previous exacerbation history, and baseline lung function was found among the included RCTs, although statistical or methodological heterogeneity was not significant. Second, our study pooled data primarily from the study populations that benefited from triple therapy. Therefore, our results should be applied to patients with COPD who are expected to have additional benefit from triple therapy. For example, adding LD-ICS to LABA/LAMA for FEV_1_ or SGRQ improvement in patients with COPD without exacerbation history is a misinterpretation of the results. Third, we arbitrarily classified ICS doses into low, medium, and high based on the reference for asthma patients. As there has been no official consensus for ICS dose classification in COPD, several studies have classified ICS doses in patients with COPD based on the guidelines as per the Global Initiative for Asthma (GINA) report^67^. Further study is necessary to evaluate the efficacy and safety of different doses of ICS in patients with COPD. Fourth, since 2016, there have been few studies evaluating triple therapy with HD-ICS, and the number of related studies is relatively small. For a more definitive conclusion, further research on triple therapy with HD-ICS is needed. Fifth, sensitivity analyses according to blood eosinophil counts were not performed because the range of the mean blood eosinophil counts was narrow (150–250/μL), and sufficient networks could not be generated among the different ICS doses.

## Conclusion

There were no significant differences in efficacy and safety among triple therapies with different ICS doses. Among patients with COPD, triple therapy with HD-ICS may reduce exacerbation in specific subgroups (treatment duration ≥ 48 weeks, FEV1 < 65%, and previous exacerbation history) with a low certainty of evidence.

## Methods

### Protocol and pre-registration

We drafted the study protocol as per the Preferred Reporting Items for Systematic Review and Meta-Analysis (PRISMA) extension statement for the reporting of SRs incorporating NMAs on healthcare interventions^68^, and also referred to the updated PRISMA 2020 statement^69^. We followed the BayesWatch guidelines for reporting our results obtained using Bayesian statistics^70^. The study protocol was previously registered on the international prospective register of systematic reviews (CRD42021259602, PROSPERO).

### Eligibility criteria

Eligible studies met the following inclusion criteria: (1) parallel-design RCTs on COPD with information on acute exacerbation as a prespecified outcome; (2) including patients with stable COPD aged > 40 years; (3) inhaled treatment with triple therapy or ICS/LABA/LAMA; and (4) treatment duration of at least 12 weeks.

### Study outcome

The primary outcome was total and moderate-to-severe exacerbation events in patients who used triple therapy with different ICS doses. We defined LD-, MD-, and HD-ICS according to the guidelines as per the GINA report^71^. The secondary outcome was all-cause mortality, change in morning trough FEV_1_ and SGRQ, and safety profiles including serious adverse events, serious cardiac adverse events, and pneumonia.

Sensitivity and subgroup analyses were conducted to identify specific subpopulations in patients with COPD with different efficacy or safety profiles for reducing moderate-to-severe or total exacerbations according to baseline lung function, previous exacerbation history, severity of dyspnea (CAT ≥ 10 or mMRC score ≥ 2), mean blood eosinophil count, and study duration.

### Information sources

We searched for relevant articles or abstracts that were registered in PubMed, EMBASE, and the Cochrane Library. To obtain more information on unpublished data, we searched the European Union (EU) Clinical Trial Register, the United States (US) National Library of Medicine, and the websites of several pharmaceutical companies including AstraZeneca, Boehringer Ingelheim, GlaxoSmithKline, and Novartis. We contacted the corresponding author or the person in charge for each study to request undisclosed information. We manually searched previously published SRs for relevant references.

### Search strategy

Our search strategy was formulated based on the Peer Review of Electronic Search Strategies checklist (search date: June 30, 2022)^72^. Controlled vocabularies and free texts were used to create the queries. The following keywords were used: “COPD” AND inhaled drugs (“ICS” AND “LABA” AND “LAMA”) AND randomized controlled design. Detailed information on the search strategy is described in Supplementary information 15 and the pre-registered study protocol.

### Study selection

Two independent authors (H.W.L. and H.M.P.) performed the process for selecting eligible RCTs in accordance with the currently updated PRISMA flow diagram^69^. The two authors individually checked duplicated literature with the same data source, screened the titles and abstracts to find potentially eligible studies, and fully reviewed the manuscript to finally select studies in concordance with the eligibility criteria. We resolved any disagreements during the study selection process by referring to the original article and discussing it with the third author (C.H.L.).

### Data collection

Before initiating the data extraction process, we obtained a consensus (among the authors)on the methodology for evaluating the quality of the eligible studies and that for the synthesis of the outcome variables. After a pilot format for data extraction was structured, pilot-tested, and refined, the data extraction process was independently conducted by two authors (H.W.L. and H.M.P.). Any disagreements regarding the extracted data were resolved by referring to the original manuscript and discussion with a third author (C.H.L.).

The following data items were extracted: (1) study-level baseline information (first author, published year, trial identifier, study duration, inclusion and exclusion criteria, the number of subjects included in intention-to-treat analysis, and pre-specified study objectives); (2) patient-level baseline information (age, sex, smoking history, and ethnicity); (3) clinical information (mean post-bronchodilator FEV_1_, COPD severity [GOLD stage], history of moderate or severe exacerbation of COPD, and severity of symptoms [mMRC or CAT score]); and (4) outcome information (the number of patients with acute exacerbations of COPD or mortality events, change in FEV_1_ or SGRQ, the number of patients with serious adverse events, serious cardiac adverse events, or pneumonia until the last follow-up). Digitizing the raw data from the Kaplan–Meier curve was allowed^73^. We defined the severity of acute COPD exacerbation based on Exacerbations of Chronic Pulmonary Disease Tool or the use of healthcare resources^74,75^. The definition of moderate exacerbation was as follows: worsening respiratory symptoms that required systemic corticosteroids or antibiotics. The definition of severe exacerbation was as follows: worsening respiratory symptoms that required hospitalization or visit to the emergency room. Serious adverse events were defined based on the Office for Human Research Protections guidelines, as follows: any condition resulting in death, life-threatening status, hospitalization or prolonged hospitalization, significant disability or incapacity, and congenital defects based on physician’s judgment^76^. As the majority of the included studies did not report major adverse cardiovascular events with sufficient clarity, serious cardiac adverse events were evaluated. Serious cardiovascular events were defined as ≥ 3 grade cardiovascular events based on the Common Terminology Criteria for Adverse Events^77^.

### Network geometry

The geometry of the treatment network was explored to identify the following: the number of triple therapies classified as low, medium, or high ICS dose therapies; the inhaled drugs that were directly compared; the number of patients assigned to receive each inhaled drug; the inhaled drugs or comparisons that were either preferred or avoided. To depict the geometry of the network, we expressed each individual inhaled drug or combination therapy as a node and each comparison between two different interventions as an edge between nodes. The number of direct comparisons between two different interventions was described as the thickness of the edge and expressed at the middle of the edges.

### ROB assessment within and across individual studies

The ROB at the individual study level was independently assessed by two reviewers (H.W.L. and H.M.P.) using the Cochrane ROB^78^ and ROB 2 tool^79^. Publication bias was used as a metric for the assessment of ROB across studies; we used funnel plots and Egger’s tests to detect publication bias. We tested for selective reporting by referring to the pre-registered study protocol. Any disagreements related to the assessment of ROB was resolved by discussion with the other authors.

### Summary measures and analysis method

A random effects model was optimized with a heterogeneous variance structure for the present NMA, because the variances of the efficacy and safety outcomes were assumed to be different among triple therapies with different ICS doses^80,81^. We adopted non-informative prior distributions assuming the existence of a normal or uniform distribution^82^. Triple therapies with different ICS doses were ranked based on the probability of the best treatment using the SUCRA methodology^83^. The median posterior ORs with 95% CrIs for categorical outcomes and the median posterior mean difference with 95% CrIs for continuous outcomes were derived from the posterior distributions. Statistical significance was determined if the 95% CrIs did not include 1.0 for an OR and zero for a mean difference. For direct comparisons among inhaled therapies, pairwise meta-analyses were conducted with a random effects model.

We used the “gemtc” and “BUGSnet” packages in R software, version 4.0.5 [R Core Team (2018), Vienna, Austria] to simulate the posterior probability distribution of each parameter using the Markov Chain Monte Carlo (MCMC) method. We checked the convergence of the results from the MCMC simulations using trace plots, autocorrelation plots, and Gelman-Rubin statistics.

### Testing of assumptions

The homogeneity and transitivity assumptions were assessed by reviewing the inclusion criteria of the included RCTs and baseline characteristics of the included study subjects. The consistency assumption was assessed by using the node-splitting method^84^. The heterogeneity across studies was assessed using the posterior median value of the standard deviation (SD) between studies^85,86^. We appraised the transitivity assumption by checking whether all the treatment options in the network were randomized by reviewing the inclusion criteria in the included studies^87^.

### Certainty of evidence

We rated the quality of evidence based on five domains (ROB, inconsistency, indirectness, imprecision, and publication bias) using the GRADE (Grading of Recommendations, Assessment, Development, and Evaluations) guidelines.

## Supplementary Information

Supplementary Information 1.

Supplementary Information 2.

## Data Availability

The datasets generated and/or analyzed during the current study are not publicly available due the fact that the journals the published the included studies have the copyrights for the information used in our meta-analysis, but are available from the corresponding author on reasonable request.

## References

[1] Pauwels RA (1999). Long-term treatment with inhaled budesonide in persons with mild chronic obstructive pulmonary disease who continue smoking. European Respiratory Society Study on Chronic Obstructive Pulmonary Disease. N. Engl. J. Med..

[2] Burge PS (2000). Randomised, double blind, placebo controlled study of fluticasone propionate in patients with moderate to severe chronic obstructive pulmonary disease: The ISOLDE trial. BMJ (Clin. Res. Ed.).

[3] Wise R, Connett J, Weinmann G, Scanlon P, Skeans M (2000). Effect of inhaled triamcinolone on the decline in pulmonary function in chronic obstructive pulmonary disease. N. Engl. J. Med..

[4] Ito K (2005). Decreased histone deacetylase activity in chronic obstructive pulmonary disease. N. Engl. J. Med..

[5] Calverley P (2003). Combined salmeterol and fluticasone in the treatment of chronic obstructive pulmonary disease: A randomised controlled trial. Lancet.

[6] Calverley PM (2007). Salmeterol and fluticasone propionate and survival in chronic obstructive pulmonary disease. N. Engl. J. Med..

[7] Lipson DA (2020). Reduction in all-cause mortality with fluticasone furoate/umeclidinium/vilanterol in patients with chronic obstructive pulmonary disease. Am. J. Respir. Crit. Care Med..

[8] Martinez FJ (2021). Reduced all-cause mortality in the ETHOS trial of budesonide/glycopyrrolate/formoterol for chronic obstructive pulmonary disease. A randomized, double-blind, multicenter, parallel-group study. Am. J. Respir. Crit. Care Med..

[9] Lee HW, Park J, Jo J, Jang EJ, Lee CH (2019). Comparisons of exacerbations and mortality among regular inhaled therapies for patients with stable chronic obstructive pulmonary disease: Systematic review and Bayesian network meta-analysis. PLoS Med..

[10] Crim C (2009). Pneumonia risk in COPD patients receiving inhaled corticosteroids alone or in combination: TORCH study results. Eur. Respir. J..

[11] Lee CH (2013). Risk of hospital admission or emergency room visit for pneumonia in patients using respiratory inhalers: A case-crossover study. Respirology.

[12] Drummond MB, Dasenbrook EC, Pitz MW, Murphy DJ, Fan E (2008). Inhaled corticosteroids in patients with stable chronic obstructive pulmonary disease: A systematic review and meta-analysis. JAMA.

[13] Izquierdo JL, Cosio BG (2018). The dose of inhaled corticosteroids in patients with COPD: When less is better. Int. J. Chron. Obstruct. Pulmon. Dis..

[14] Masoli M, Holt S, Weatherall M, Beasley R (2004). Dose-response relationship of inhaled budesonide in adult asthma: A meta-analysis. Eur. Respir. J..

[15] Masoli M, Weatherall M, Holt S, Beasley R (2004). Clinical dose-response relationship of fluticasone propionate in adults with asthma. Thorax.

[16] Rogliani P, Ritondo BL, Calzetta L (2021). Triple therapy in uncontrolled asthma: A network meta-analysis of phase III studies. Eur. Respir. J..

[17] Rabe KF (2020). Triple inhaled therapy at two glucocorticoid doses in moderate-to-very-severe COPD. N. Engl. J. Med..

[18] Aaron SD (2007). Tiotropium in combination with placebo, salmeterol, or fluticasone-salmeterol for treatment of chronic obstructive pulmonary disease: A randomized trial. Ann. Intern. Med..

[19] Cazzola M (2007). A pilot study to assess the effects of combining fluticasone propionate/salmeterol and tiotropium on the airflow obstruction of patients with severe-to-very severe COPD. Pulm. Pharmacol. Ther..

[20] Magnussen H (2014). Withdrawal of inhaled glucocorticoids and exacerbations of COPD. N. Engl. J. Med..

[21] Frith PA (2015). Glycopyrronium once-daily significantly improves lung function and health status when combined with salmeterol/fluticasone in patients with COPD: The GLISTEN study, a randomised controlled trial. Thorax.

[22] Chapman KR (2018). Long-term triple therapy de-escalation to indacaterol/glycopyrronium in patients with chronic obstructive pulmonary disease (SUNSET): A randomized, double-blind, triple-dummy clinical trial. Am. J. Respir. Crit. Care Med..

[23] Register, E. C. T. A multinational, multicentre, randomised, open-label, active-controlled, 26-week, 2-arm, parallel group study to evaluate the non-inferiority of fixed combination of beclometasone dipropionate plus formoterol fumarate plus glycopyrronium bromide administered via pMDI (CHF 5993) versus fixed combination of fluticasone furoate plus vilanterol administered via DPI (Relvar^®^) plus tiotropium bromide (Spiriva^®^) for the treatment of patients with chronic obstructive pulmonary disease. https://www.clinicaltrialsregister.eu/ctr-search/trial/2014-001487-35/results (Accessed 30 June 2021).

[24] Ferguson GT (2020). Once-daily single-inhaler versus twice-daily multiple-inhaler triple therapy in patients with COPD: Lung function and health status results from two replicate randomized controlled trials. Respir. Res..

[25] Welte T (2009). Efficacy and tolerability of budesonide/formoterol added to tiotropium in patients with chronic obstructive pulmonary disease. Am. J. Respir. Crit. Care Med..

[26] Hanania NA (2012). Benefits of adding fluticasone propionate/salmeterol to tiotropium in moderate to severe COPD. Respir. Med..

[27] Jung KS (2012). Comparison of tiotropium plus fluticasone propionate/salmeterol with tiotropium in COPD: A randomized controlled study. Respir. Med..

[28] Lee SD (2016). Efficacy and tolerability of budesonide/formoterol added to tiotropium compared with tiotropium alone in patients with severe or very severe COPD: A randomized, multicentre study in East Asia. Respirology.

[29] Siler TM, Kerwin E, Singletary K, Brooks J, Church A (2016). Efficacy and safety of umeclidinium added to fluticasone propionate/salmeterol in patients with COPD: Results of two randomized, double-blind studies. COPD.

[30] Singh D (2016). Single inhaler triple therapy versus inhaled corticosteroid plus long-acting β2-agonist therapy for chronic obstructive pulmonary disease (TRILOGY): A double-blind, parallel group, randomised controlled trial. Lancet.

[31] Vestbo J (2017). Single inhaler extrafine triple therapy versus long-acting muscarinic antagonist therapy for chronic obstructive pulmonary disease (TRINITY): A double-blind, parallel group, randomised controlled trial. Lancet.

[32] Ferguson GT (2018). Triple therapy with budesonide/glycopyrrolate/formoterol fumarate with co-suspension delivery technology versus dual therapies in chronic obstructive pulmonary disease (KRONOS): A double-blind, parallel-group, multicentre, phase 3 randomised controlled trial. Lancet Respir. Med..

[33] Papi A (2018). Extrafine inhaled triple therapy versus dual bronchodilator therapy in chronic obstructive pulmonary disease (TRIBUTE): A double-blind, parallel group, randomised controlled trial. Lancet.

[34] Zheng J (2021). Efficacy and safety of single-inhaler extrafine triple therapy versus inhaled corticosteroid plus long-acting beta2 agonist in eastern Asian patients with COPD: The TRIVERSYTI randomised controlled trial. Respir. Res..

[35] Siler TM (2015). Efficacy and safety of umeclidinium added to fluticasone furoate/vilanterol in chronic obstructive pulmonary disease: Results of two randomized studies. Respir. Med..

[36] Lipson DA (2017). FULFIL trial: Once-daily triple therapy for patients with chronic obstructive pulmonary disease. Am. J. Respir. Crit. Care Med..

[37] Lipson DA (2018). Once-daily single-inhaler triple versus dual therapy in patients with COPD. N. Engl. J. Med..

[38] Zhao D, Ling C, Guo Q, Jin J, Xu H (2018). Efficacy and safety of tiotropium bromide combined with budesonide/formoterol in the treatment of moderate to severe chronic obstructive pulmonary disease. Exp. Ther. Med..

[39] Bansal S (2021). Single-inhaler fluticasone furoate/umeclidinium/vilanterol (FF/UMEC/VI) triple therapy versus tiotropium monotherapy in patients with COPD. NPJ Prim. Care Respir. Med..

[40] Chung KF (2014). International ERS/ATS guidelines on definition, evaluation and treatment of severe asthma. Eur. Respir. J..

[41] Israel E, Reddel HK (2017). Severe and difficult-to-treat asthma in adults. N. Engl. J. Med..

[42] Barnes PJ (2019). Inflammatory endotypes in COPD. Allergy.

[43] Dejager L (2015). Neutralizing TNFα restores glucocorticoid sensitivity in a mouse model of neutrophilic airway inflammation. Mucosal Immunol..

[44] Milara J (2022). The pan-JAK inhibitor LAS194046 reduces neutrophil activation from severe asthma and COPD patients in vitro. Sci. Rep..

[45] O'Donnell RA (2004). Relationship between peripheral airway dysfunction, airway obstruction, and neutrophilic inflammation in COPD. Thorax.

[46] Hogg JC (2004). The nature of small-airway obstruction in chronic obstructive pulmonary disease. N. Engl. J. Med..

[47] Hurst JR, Perera WR, Wilkinson TM, Donaldson GC, Wedzicha JA (2006). Systemic and upper and lower airway inflammation at exacerbation of chronic obstructive pulmonary disease. Am. J. Respir. Crit. Care Med..

[48] Ozol D (2005). The effect of inhaled corticosteroids on bronchoalveolar lavage cells and IL-8 levels in stable COPD patients. Respir. Med..

[49] Cazzola M (2016). Interaction between corticosteroids and muscarinic antagonists in human airways. Pulm. Pharmacol. Ther..

[50] Yamada M, Ichinose M (2018). The cholinergic pathways in inflammation: A potential pharmacotherapeutic target for COPD. Front. Pharmacol..

[51] Miller-Larsson A, Selroos O (2006). Advances in asthma and COPD treatment: Combination therapy with inhaled corticosteroids and long-acting beta 2-agonists. Curr. Pharm. Des..

[52] Newton R, Giembycz MA (2016). Understanding how long-acting β(2)-adrenoceptor agonists enhance the clinical efficacy of inhaled corticosteroids in asthma—An update. Br. J. Pharmacol..

[53] Rogliani P (2020). Beclomethasone dipropionate, formoterol fumarate and glycopyrronium bromide: Synergy of triple combination therapy on human airway smooth muscle ex vivo. Br. J. Pharmacol..

[54] Sin DD, Anthonisen NR, Soriano JB, Agusti AG (2006). Mortality in COPD: Role of comorbidities. Eur. Respir. J..

[55] Loke YK, Kwok CS, Singh S (2010). Risk of myocardial infarction and cardiovascular death associated with inhaled corticosteroids in COPD. Eur. Respir. J..

[56] Vestbo J (2016). Fluticasone furoate and vilanterol and survival in chronic obstructive pulmonary disease with heightened cardiovascular risk (SUMMIT): A double-blind randomised controlled trial. Lancet.

[57] Celli BR (2008). Effect of pharmacotherapy on rate of decline of lung function in chronic obstructive pulmonary disease: Results from the TORCH study. Am. J. Respir. Crit. Care Med..

[58] Zheng Y (2018). Triple therapy in the management of chronic obstructive pulmonary disease: Systematic review and meta-analysis. BMJ.

[59] Ernst P, Gonzalez AV, Brassard P, Suissa S (2007). Inhaled corticosteroid use in chronic obstructive pulmonary disease and the risk of hospitalization for pneumonia. Am. J. Respir. Crit. Care Med..

[60] Brassard P, Suissa S, Kezouh A, Ernst P (2011). Inhaled corticosteroids and risk of tuberculosis in patients with respiratory diseases. Am. J. Respir. Crit. Care Med..

[61] Suissa S, Kezouh A, Ernst P (2010). Inhaled corticosteroids and the risks of diabetes onset and progression. Am. J. Med..

[62] Loke YK, Cavallazzi R, Singh S (2011). Risk of fractures with inhaled corticosteroids in COPD: Systematic review and meta-analysis of randomised controlled trials and observational studies. Thorax.

[63] Smeeth L, Boulis M, Hubbard R, Fletcher AE (2003). A population based case-control study of cataract and inhaled corticosteroids. Br. J. Ophthalmol..

[64] Finney L (2014). Inhaled corticosteroids and pneumonia in chronic obstructive pulmonary disease. Lancet Respir. Med..

[65] Sutton AJ (2002). Meta-analysis of rare and adverse event data. Expert Rev. Pharmacoecon. Outcomes Res..

[66] Jansen JP, Crawford B, Bergman G, Stam W (2008). Bayesian meta-analysis of multiple treatment comparisons: An introduction to mixed treatment comparisons. Value Health.

[67] Agusti A (2018). Inhaled corticosteroids in COPD: Friend or foe?. Eur. Respir. J..

[68] Hutton B (2015). The PRISMA extension statement for reporting of systematic reviews incorporating network meta-analyses of health care interventions: Checklist and explanations. Ann. Intern. Med..

[69] Page MJ (2021). The PRISMA 2020 statement: An updated guideline for reporting systematic reviews. PLoS Med..

[70] Spiegelhalter DJ, Myles JP, Jones DR, Abrams KR (2000). Bayesian methods in health technology assessment: A review. Health Technol. Assess..

[71] Global Strategy for Asthma Management and Prevention. Global Strategy for Asthma Management and Prevention, http://www.ginasthma.org (2022).

[72] McGowan J (2016). PRESS peer review of electronic search strategies: 2015 guideline statement. J. Clin. Epidemiol..

[73] Liu Z, Rich B, Hanley JA (2014). Recovering the raw data behind a non-parametric survival curve. Syst. Rev..

[74] Mackay AJ (2014). Detection and severity grading of COPD exacerbations using the exacerbations of chronic pulmonary disease tool (EXACT). Eur. Respir. J..

[75] Burge S, Wedzicha JA (2003). COPD exacerbations: Definitions and classifications. Eur. Respir. J. Suppl..

[76] Health, U. D. o. & Services, H. (2018).

[77] Program, N. C. I. C. T. E. *Common Terminology Criteria for Adverse Events:(CTCAE)*. (Cancer Therapy Evaluation Program, 2003).

[78] Sterne JAC (2019). RoB 2: A revised tool for assessing risk of bias in randomised trials. BMJ.

[79] Higgins JP (2011). The Cochrane Collaboration's tool for assessing risk of bias in randomised trials. BMJ.

[80] Lu G, Ades AE (2004). Combination of direct and indirect evidence in mixed treatment comparisons. Stat. Med..

[81] Dias, S., Welton, N. J., Sutton, A. J. & Ades, A. E. In *NICE DSU Technical Support Document 2: A Generalised Linear Modelling Framework for Pairwise and Network Meta-Analysis of Randomised Controlled Trials* (National Institute for Health and Care Excellence (NICE) Copyright © 2014 National Institute for Health and Clinical Excellence, unless otherwise stated. All rights reserved., 2014). 27466657

[82] Carlin, B. P., Hong, H., Shamliyan, T. A., Sainfort, F. & Kane, R. L. In *Case Study Comparing Bayesian and Frequentist Approaches for Multiple Treatment Comparisons* (Agency for Healthcare Research and Quality (US), 2013). 23638487

[83] Lee HW, Kim HJ, Jang EJ, Lee CH (2021). Comparisons of efficacy and safety between triple (inhaled corticosteroid/long-acting muscarinic antagonist/long-acting beta-agonist) therapies in chronic obstructive pulmonary disease: Systematic review and Bayesian network meta-analysis. Respir. Int. Rev. Thorac. Dis..

[84] Dias S, Welton NJ, Caldwell DM, Ades AE (2010). Checking consistency in mixed treatment comparison meta-analysis. Stat. Med..

[85] Spiegelhalter, D. J., Abrams, K. R. & Myles, J. P. *Bayesian Approaches to Clinical Trials and Health-Care Evaluation*. Vol. 13 (Wiley, 2004).

[86] Moran JL, Graham PL, Rockliff S, Bersten ADJCC (2010). Updating the evidence for the role of corticosteroids in severe sepsis and septic shock: A Bayesian meta-analytic perspective. Crit. Care.

[87] Salanti G (2012). Indirect and mixed-treatment comparison, network, or multiple-treatments meta-analysis: Many names, many benefits, many concerns for the next generation evidence synthesis tool. Res. Synth. Methods.

