# Supplementary material for: Different inhaled corticosteroid doses in triple therapy for chronic obstructive pulmonary disease: systematic review and Bayesian network meta-analysis
Source: Sci Rep. 2022 Sep 20;12:15698. doi: 10.1038/s41598-022-18353-y (PMC9489688; doi:10.1038/s41598-022-18353-y)
Supplement: Supplementary file 1 — Supplementary Information 1. [file 41598_2022_18353_MOESM1_ESM.docx]

**Supplementary information 1. Excluded references based on full-text review**

| **No.** | **Study title** | **Identifier** | **Author** | **Journal (published year)** | **Major reason for exclusion** |
| --- | --- | --- | --- | --- | --- |
| **Identified studies via databases and registers** | | | | | |
| 1 | A Comparative Study Between Fluticasone Furoate/Umeclidinium/Vilanterol (FF/UMEC/VI) Single Inhaler Triple Therapy Versus Tiotropium Monotherapy in Subjects With Chronic Obstructive Pulmonary Disease (COPD) | NCT03474081 | - | Unpublished | This reference did not describe the exacerbation rate or mortality rate, which were the outcomes of our meta-analysis. |
| 2 | A Randomized Study, Comparing Fluticasone Furoate/Umeclidinium/Vilanterol (FF/UMEC/VI) Single Inhaler Triple Therapy, Versus Multiple Inhaler Therapy (Budesonide/Formoterol Plus Tiotropium) in Subjects With Chronic Obstructive Pulmonary Disease (COPD) | NCT03478683 | - | Unpublished | This reference did not describe the exacerbation rate or mortality rate, which were the outcomes of our meta-analysis. |
| 3 | A Randomized, Open Label, Multicenter, Phase 4 Study For The Comparison Of Efficacy Of Tiotropium Plus Salmeterol/ Fluticasone Propionate Compared With Tiotropium Alone In COPD Patients | NCT00864812 | - | Unpublished | This reference did not describe the exacerbation rate or mortality rate, which were the outcomes of our meta-analysis. |
| 4 | Active Controlled Trial Of Chf5993 Pressurized Metered-Dose Inhaler (pMDI) Vs Symbicort Turbuhaler In Patients With Chronic Obstructive Pulmonary Disease (COPD) (TRIVERSYTI) | NCT03197818 | - | Unpublished | This reference did not describe the exacerbation rate or mortality rate, which were the outcomes of our meta-analysis. |
| 5 | Assessment In a Real World Setting of the Effect of Inhaled Steroid-based Triple Therapy Versus the Combination of Tiotropium and Olodaterol on Reducing Chronic Obstructive Pulmonary Disease (COPD) Exacerbations [AIRWISE] | NCT03265145 | - | Unpublished | This reference did not describe the exacerbation rate or mortality rate, which were the outcomes of our meta-analysis. |
| 6 | Assessment Of Peak Inspiratory Flow Rate In Patients With Chronic Obstructive Pulmonary Disease: Impact On Dose Delivery And Relationship With Response To Fluticasone Furoate/Umeclidinium/Vilanterol Triple Therapy | NCT03478683 NCT03478696 | Anderson et al. | American journal of respiratory and critical care medicine (2020) (Abstract) | This study was eligible for our study, but we used another reference with the same data source. |
| 7 | Benefits Of Budesonide-Containing Therapies On Reducing Lung Function Decline In Patients With COPD In The ETHOS Study | NCT02465567 | Ferguson et al. | Chest (2020) (Abstract) | This study was eligible for our study, but we used another reference with the same data source. |
| 8 | Clinical Effects Of Adding Fluticasone Propionate/Salmeterol (FSC) And Tiotropium (TIO) In Severe-To-Very Severe COPD | Not available | D'Amato et al. | European Respiratory Journal (2005) (Abstract) | This study was eligible for our study, but we used another reference with the same data source. |
| 9 | Combination Of Inhaled Salmeterol/Fluticasone And Tiotropium In The Treatment Of Chronic Obstructive Pulmonary Disease: A Randomised Controlled Trial | Not available | Fang et al. | Chinese journal of tuberculosis and respiratory diseases (2008) | This reference did not describe the exacerbation rate or mortality rate, which were the outcomes of our meta-analysis. |
| 10 | Comparative Study of Fluticasone Furoate(FF)/Umeclidinium Bromide (UMEC)/ Vilanterol (VI) Closed Therapy Versus FF/VI Plus UMEC Open Therapy in Subjects With Chronic Obstructive Pulmonary Disease (COPD) | NCT02729051 | - | Unpublished | The study intervention did not meet the eligibility criteria of our study. |
| 11 | Cost Effectiveness Of Adding Budesonide/Formoterol To Tiotropium In COPD In Four Nordic Countries | NCT00496470 | Nielsen et al. | Respiratory medicine (2013) | This study was eligible for our study, but we used another reference with the same data source. |
| 12 | Cost Effectiveness Of Therapy With Combinations Of Long Acting Bronchodilators And Inhaled Steroids For Treatment Of COPD | ISRCTN29870041 | Najafzadeh et al. | Thorax (2008) | This study was eligible for our study, but we used another reference with the same data source. |
| 13 | Cost-Effectiveness Of Umeclidinium As Add-On To ICS/LABA Therapy In COPD: A UK Perspective | NCT01957163 NCT02119286 NCT01772134 NCT01772147 NCT02257372 | Driessen et al. | Respiratory Medicine (2018) | This study was a pooled analysis of previous RCTs. |
| 14 | Effect Of Fluticasone-Salmeterol Combination+Tiotropium Vs Tiotropium On Exercise Tolerance, The Cause Of Exercise Limitation And Lung Volumes In COPD | NCT01124422 | Maltais et al. | American journal of respiratory and critical care medicine (2012) (Abstract) | The intervention duration of this study was less than 12 weeks. |
| 15 | Efficacy And Safety Of Once-Daily Umeclidinium Added To Fluticasone Furoate/Vilanterol In Chronic Obstructive Pulmonary Disease: Results Of Two Replicate Randomized 12-Week Studies | NCT01772134 NCT01772147 | Siler et al. | Chest (2014) (Abstract) | This study was eligible for our study, but we used another reference with the same data source. |
| 16 | Efficacy And Tolerability Of Budesonide/Formoterol (B/F) Added To Tiotropium (T) Vs T Alone In East-Asian Patients (Pts) With Severe/Very Severe Chronic Obstructive Pulmonary Disease (COPD) | NCT01397890 | Do et al. | European Respiratory Journal (2014) (Abstract) | This study was eligible for our study, but we used another reference with the same data source. |
| 17 | Efficacy Of Budesonide/Formoterol And Tiotropium Combination For The Treatment Of Chinese Patients With Chronic Obstructive Pulmonary Disease | Not available | Feng et al. | Medicine (Baltimore) (2018) | This study was not designed as a parallel group RCT. |
| 18 | Efficacy of Fixed Combination of Beclomethasone Dipropionate (BDP) + Formoterol Fumarate (FF) + Glycopyrronium Bromide (GB) (CHF 5993)Administered Via Dry Powder Inhaler (DPI) in Chronic Obstructive Pulmonary Disease (COPD) (TRI-D) | NCT03590379 | - | Unpublished | This reference did not describe the exacerbation rate or mortality rate, which were the outcomes of our meta-analysis. |
| 19 | Exacerbation Benefit By Blood Eosinophil Counts With Budesonide/Glycopyrronium/Formoterol Metered Dose Inhaler (BGF MDI) At Two Ics Dose Levels In The ETHOS Trial: A Subgroup Analysis | NCT02465567 | Rabe et al. | European Respiratory Journal (2020) (Abstract) | This study was eligible for our study, but we used another reference with the same data source. |
| 20 | Intrepid: Clinical Effectiveness Of Once-Daily Single-Inhaler Fluticasone Furoate/Umeclidinium/Vilanterol Versus Multiple-Inhaler Triple Therapy In Usual Clinical Practice | NCT03467425 | Halpin et al. | American journal of respiratory and critical care medicine (2020) (Abstract) | The study intervention did not meet the eligibility criteria of our study because various doses of ICS were allowed in non-ELLIPTA maintenance therapy group. |
| 21 | INTREPID: Investigation of TRELEGY Effectiveness: Usual Practice Design | NCT03467425 | - | Unpublished | The study intervention did not meet the eligibility criteria of our study because various doses of ICS were allowed in non-ELLIPTA maintenance therapy group. |
| 22 | Once-Daily Triple Therapy in Patients with COPD: Patient-Reported Symptoms and Quality of Life | NCT02345161 | Tabberer et al. | Advances in Therapy (2018) | This study was eligible for our study, but we used another reference with the same data source. |
| 23 | Pharmacokinetics Of Budesonide/Glycopyrrolate/Formoterol Fumarate Metered Dose Inhaler Formulated Using Co-Suspension Delivery Technology After Single And Chronic Dosing In Patients With COPD | NCT02497001 | Dunn et al. | Pulmonary Pharmacology & Therapeutics (2020) | This study was eligible for our study, but we used another reference with the same data source. |
| 24 | Preventing Clinically Important Deterioration With Single-Inhaler Triple Therapy In COPD | NCT02345161 | Naya et al. | ERJ Open Research (2018) | This study was eligible for our study, but we used another reference with the same data source. |
| 25 | Randomized Controlled Trial Of Glycopyrronium Added To Fixed Combination Salmeterol-Fluticasone In COPD: Primary Care And Specialist Site Differences In The Glisten Study | NCT01513460 | Thompson et al. | Respirology (2015) (Abstract) | This study was eligible for our study, but we used another reference with the same data source. |
| 26 | Single-Inhaler Fluticasone Furoate/Umeclidinium/Vilanterol Versus Fluticasone Furoate/Vilanterol Plus Umeclidinium Using Two Inhalers For Chronic Obstructive Pulmonary Disease: A Randomized Non-Inferiority Study | NCT02729051 | Bremner et al. | Respiratory Research (2018) | The study intervention did not meet the eligibility criteria of our study. |
| 27 | Single-Inhaler Triple Therapy Fluticasone Furoate/Umeclidinium/Vilanterol Compared With Tiotropium Monotherapy In Chronic Obstructive Pulmonary Disease: A Post Hoc Analysis By Airflow Limitation | NCT03474081 | Anzueto et al. | Chest (2020) (Abstract) | This study is a post-hoc analysis of another reference with the same data source. We could not obtain additional information from this reference. |
| 28 | Superiority Of "Triple" Therapy With Salmeterol/Fluticasone Propionate And Tiotropium Bromide Versus Individual Components In Moderate To Severe COPD | NCT00325169 | Singh et al. | Thorax (2008) | The intervention duration of this study was less than 12 weeks. |
| 29 | The Effect Of Umeclidinium Added To Inhaled Corticosteroid/Long-Acting Beta2-Agonist In Patients With Symptomatic COPD: A Randomised, Double-Blind, Parallel-Group Study | NCT02257372 | Sousa et al. | NPJ primary care respiratory medicine (2016) | Various combinations of ICS/LABA were included in ICS/LAMA/LABA groups. The study intervention did not meet the eligibility criteria of our study. |
| 30 | Treatment Options For Patients With Allergic Phenotype Coexisting Chronic Obstructive Pulmonary Disease: Is It Better Approach For Treatment? | Not available | Sadigov et al. | American journal of respiratory and critical care medicine (2014) (Abstract) | This reference did not describe the exacerbation rate or mortality rate, which were the outcomes of our meta-analysis. |
| **Identified studies via other methods** | | | | | |
| 31 | A Comparison Study Between the Fixed Dose Triple Combination of Fluticasone Furoate/ Umeclidinium/ Vilanterol Trifenatate (FF/UMEC/VI) With Budesonide/Formoterol in Subjects with Chronic Obstructive Pulmonary Disease (COPD) | GSK study identifier: 116853 | - | Unpublished | This study was eligible for our study, but we used another reference with the same data source. |
| 32 | A phase IIIB, 24-week randomised, double-blind study to compare ‘closed’ triple therapy (FF/UMEC/VI) with 'open' triple therapy (FF/VI + UMEC), in subjects with chronic obstructive pulmonary disease (COPD) | GSK study identifier: 200812 | - | Unpublished | Same triple therapy was compared between fixed and open drug combination devices. |
| 33 | A Randomized, Double Blind, Multi Center, Parallel Group Study to Assess the Efficacy and Safety of PT010 Relative to PT003 and PT009 on COPD Exacerbations over a 52 Week Treatment Period in Subjects With Moderate to Very Severe COPD | Pearl Therapeutics study identifier: PT010005 | - | Unpublished | This study was eligible for our study, but we used another reference with the same data source. |
| 34 | A phase III, 52 week, randomized, double-blind, 3-arm parallel group study, comparing the efficacy, safety and tolerability of the fixed dose triple combination FF/UMEC/VI with the fixed dose dual combinations of FF/VI and UMEC/VI, all administered once-daily in the morning via a dry powder inhaler in subjects with chronic obstructive pulmonary disease | GSK study identifier: 116855 | - | Unpublished | This study was eligible for our study, but we used another reference with the same data source. |
| 35 | A 52-week, double blind, double dummy, randomized, multinational, multicentre, 3-arm parallel group, active controlled clinical trial of fixed combination of beclometasone dipropionate plus formoterol fumarate plus glycopyrrolate bromide administered via pMDI (CHF 5993) versus tiotropium bromide and versus fixed combination of beclometasone dipropionate plus formoterol fumarate administered via pMDI and tiotropium bromide in patients with chronic obstructive pulmonary disease | Chiesi Farmaceutici study identifier: CCD-1208-PR-0090 | - | Unpublished | This study was eligible for our study, but we used another reference with the same data source. |
| 36 | A randomized, parallel group study to evaluate the effect of Umeclidinium (UMEC) added to Inhaled corticosteroid/ long-acting beta-agonist combination therapy in subjects with Chronic Obstructive Pulmonary Disease COPD | GSK study identifier: 201314 | - | Unpublished | The study intervention did not meet the eligibility criteria of our study because various doses of ICS were allowed in both intervention and control groups |
| 37 | A 12-week, double-blind, randomised, parallel group, multi-centre, study to evaluate efficacy and safety of budesonide/formoterol (Symbicort Turbuhaler) 320/9 µg one inhalation twice daily on top of tiotropium (Spiriva) 18 µg one inhalation once daily compared with tiotropium 18 µg one inhalation once daily, in patients with severe chronic obstructive pulmonary disease (COPD) | AstraZeneca study identifier: D5892C00015 | - | - | This study was eligible for our study, but we used another reference with the same data source. |

**Supplementary information 2. Risk of bias in the included studies**


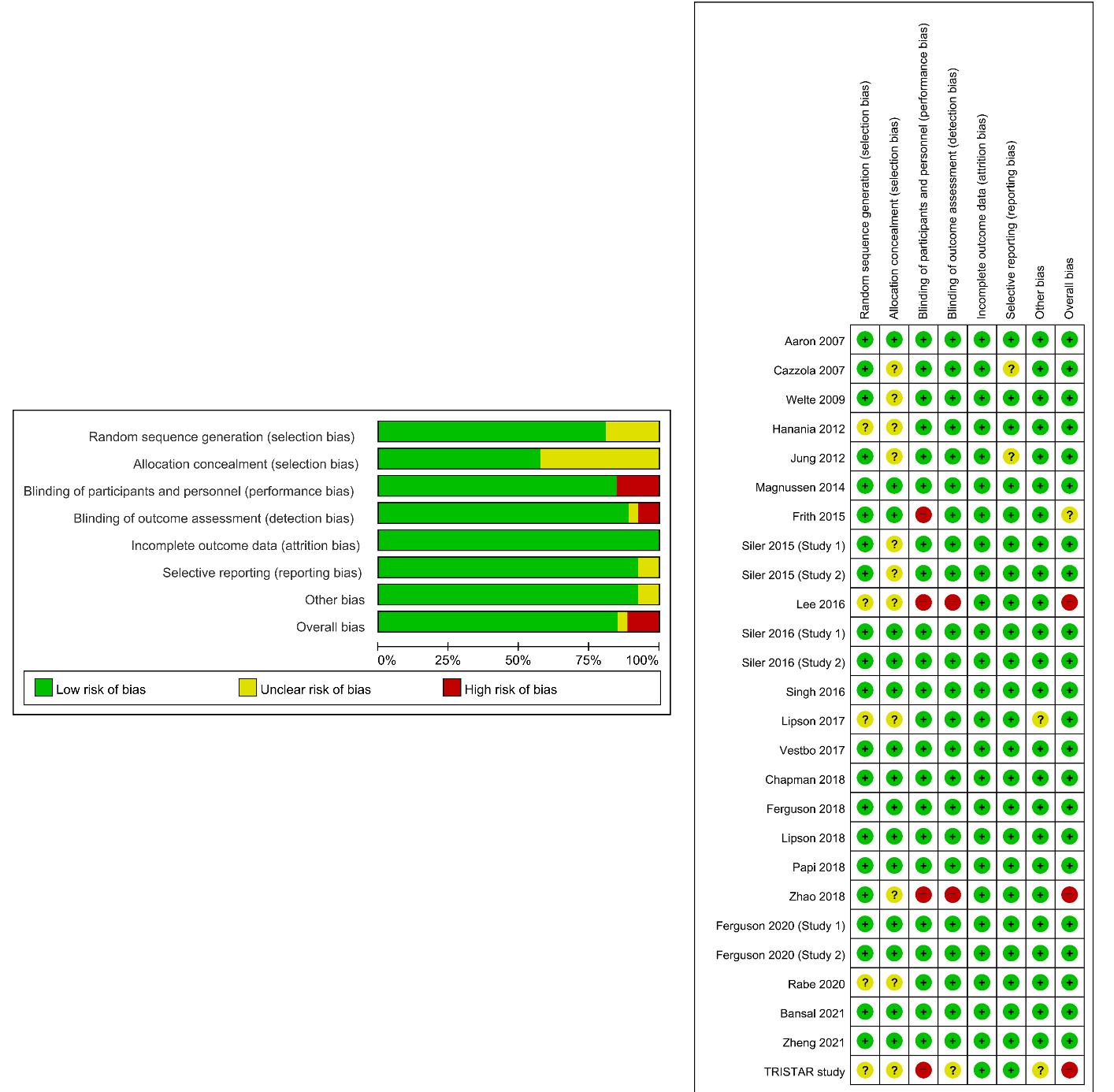


**Supplementary information 3. Detailed information on the risk of bias assessment for the eligible randomized controlled trials**

| **Author (year)** | **The risk of bias domain** | **Assessment of risk of bias and the reason for judgment** |
| --- | --- | --- |
| **Aaron et al. (2007)** | Random sequence generation | Assessment: Low risk  Reason: Random sequence was prepared from a computer-generated random listing of the three treatment allocations. |
|  | Allocation concealment | Assessment: Low risk  Reason: Randomization was performed using central allocation of a randomization schedule and research staff were not aware of the treatment assignment. |
|  | Blinding of participants and personnel | Assessment: Low risk  Reason: Neither research staff nor patients were aware of the treatment assignment before or after randomization. |
|  | Blinding of outcome assessment | Assessment: Low risk  Reason: The specific method of blinding of outcome assessment was not described. However, since our primary outcome was an objective finding (acute exacerbation), it is judged that the outcome was not likely to be influenced by the lack of blinding of outcome assessment. |
|  | Incomplete outcome data | Assessment: Low risk  Reason: Patients in both groups were followed all the way through to the end of the study. As missing data was balanced between two arms, withdrawal or follow-up loss in surrogates would not have influenced our primary and secondary outcomes. |
|  | Selective reporting | Assessment: Low risk  Reason: A pre-existing protocol was described and all the results were reported in the pre-specified manner. |
|  | Other sources of bias | Assessment: Low risk  Reason: This study appears to be free of other sources of bias. |
|  | Overall | Assessment: Low risk |
| **Cazzola et al. (2007)** | Random sequence generation | Assessment: Low risk  Reason: Randomization was achieved by a computer-generated list and performed in blocks of 9. |
|  | Allocation concealment | Assessment: Unclear risk  Reason: Although randomization was performed using a computer-generated list, the method of allocation concealment was not clearly described. |
|  | Blinding of participants and personnel | Assessment: Low risk  Reason: Same inhaler devices were used among the treatment groups. Therefore, it was hardly possible to find out which group the patient belonged to. |
|  | Blinding of outcome assessment | Assessment: Low risk  Reason: The specific method of blinding of outcome assessment was not described. Even if we assume that the investigators who conducted outcome assessment were not blinded, it was difficult for them to make any bias because treatment assignment was blinded during study and our primary outcome was an objective finding (acute exacerbation). Therefore, we concluded that the outcome assessment was unlikely to be biased in this study. |
|  | Incomplete outcome data | Assessment: Low risk  Reason: Although more patients in triple therapy group (n=29) completed 3 month treatment period compared to tiotropium (n=26) or fluticasone/salmeterol (n=26), but the difference in missing data was considered not to influence on the outcome. |
|  | Selective reporting | Assessment: Unclear risk  Reason: A pre-existing protocol was not described and we could not know that all the results were reported accordingly. |
|  | Other sources of bias | Assessment: Low risk  Reason: Sample size of this study was not adequately calculated, and type II error is strongly suspected. However, type II error in individual study will not have an effect on our pooled estimates. |
|  | Overall | Assessment: Low risk |
| **Welte et al. (2009)** | Random sequence generation | Assessment: Low risk  Reason: Random sequence was scheduled based a computer program generated at AstraZeneca. |
|  | Allocation concealment | Assessment: Unclear risk  Reason: The method of allocation concealment was not described in manuscript. There was no explanation whether the computer generated at AstraZeneca enabled allocation concealment from investigators. |
|  | Blinding of participants and personnel | Assessment: Low risk  Reason: Budesonide/Formoterol and placebo were administered via identical device, Turbuhaler, twice daily. |
|  | Blinding of outcome assessment | Assessment: Low risk  Reason: The specific method of blinding of outcome assessment was not described. Even if we assume that the investigators who conducted outcome assessment were not blinded, it was difficult for them to make any bias because treatment assignment was blinded during study and our primary outcome was an objective finding (acute exacerbation). Therefore, we concluded that the outcome assessment was unlikely to be biased in this study. |
|  | Incomplete outcome data | Assessment: Low risk  Reason: The withdrawal rate was about 8-9% and relatively even among arms. As our study extracted data from intention-to-treat participants, there was no impact on our outcomes |
|  | Selective reporting | Assessment: Low risk  Reason: A pre-existing protocol was described and all the results were reported in the pre-specified manner. |
|  | Other sources of bias | Assessment: Low risk  Reason: This study was funded by AstraZeneca and the conflict of interest exists, but appeared to be free of other sources of bias. |
|  | Overall | Assessment: Low risk |
| **Hanania et al. (2012)** | Random sequence generation | Assessment: Unclear risk  Reason: Although authors mentioned that randomization was conducted, the method of Random sequence generation was not described. |
|  | Allocation concealment | Assessment: Unclear risk  Reason: The method of allocation concealment was not described. |
|  | Blinding of participants and personnel | Assessment: Low risk  Reason: Same inhaler devices were used in both treatment groups. Therefore, it was hardly possible to find out which group the patient belonged to |
|  | Blinding of outcome assessment | Assessment: Low risk  Reason: The specific method of blinding of outcome assessment was not described. Even if we assume that the investigators who conducted outcome assessment were not blinded, it was difficult for them to make any bias because treatment assignment was blinded during study and our primary outcome was an objective finding (acute exacerbation). Therefore, we concluded that the outcome assessment was unlikely to be biased in this study. |
|  | Incomplete outcome data | Assessment: Low risk  Reason: About 20-25% of patients withdrew during study, but the reasons of withdrawal were relatively balanced between two arms. Therefore, withdrawal or follow-up loss of included subjects is not considered to have an impact on our primary and secondary outcomes. |
|  | Selective reporting | Assessment: Low risk  Reason: A pre-existing protocol was described and all the results were reported in the pre-specified manner. |
|  | Other sources of bias | Assessment: Low risk  Reason: This study was funded by GlaxoSmithKline and the conflict of interest exists, but other sources of bias was not found. |
|  | Overall | Assessment: Low risk |
| **Jung et al. (2012)** | Random sequence generation | Assessment: Low risk  Reason: Randomization was done in a 1:1 ratio through a computerized random-number generator. |
|  | Allocation concealment | Assessment: Unclear risk  Reason: Although randomization was performed using a computerized random-number generator, the method of allocation concealment was not clearly described. |
|  | Blinding of participants and personnel | Assessment: Low risk  Reason: Neither research staff nor patients were aware of the treatment assignment until randomized. |
|  | Blinding of outcome assessment | Assessment: Low risk  Reason: Although research staff was blinded from identifying treatment assignment of each participants, the specific method of blinding of outcome assessment was not described. Even if we assume that the investigators who conducted outcome assessment were not blinded, it was difficult for them to make any bias because treatment assignment was blinded during study and our primary outcome was an objective finding (acute exacerbation). Therefore, we concluded that the outcome assessment was unlikely to be biased in this study. |
|  | Incomplete outcome data | Assessment: Low risk  Reason: About 13-14% of patients dropped out during study, but the reasons of drop-out were relatively balanced between two treatments. Therefore, withdrawal or follow-up loss of included participants will not have an impact on our primary and secondary outcomes |
|  | Selective reporting | Assessment: Unclear risk  Reason: A pre-existing protocol was not prospectively registered in database, and we could not assure that all the results were reported accordingly. |
|  | Other sources of bias | Assessment: Low risk  Reason: This study was funded by GlaxoSmithKline, AstraZeneca Korea, Boehringer, Nycomed, Altana Pahrma, and Abbott. The conflict of interest may exist, but other sources of bias is not identified. |
|  | Overall | Assessment: Low risk |
| **Magnussen et al. (2014)** | Random sequence generation | Assessment: Low risk  Reason: The randomization list was generated using a validated system. The system which involved a pseudo-random number generator enabled the treatment allocation to be both reproducible and non-predictable. |
|  | Allocation concealment | Assessment: Low risk  Reason: The randomization code will be kept by Clinical Trial Support up to database lock. |
|  | Blinding of participants and personnel | Assessment: Low risk  Reason: All doses of fluticasone and placebo will be provided in a blinding device. |
|  | Blinding of outcome assessment | Assessment: Low risk  Reason: Persons directly involved in the conduct of the trial have no access to the treatment allocation prior to database lock, which takes place after the trial is officially completed and all data queries have been answered. |
|  | Incomplete outcome data | Assessment: Low risk  Reason: The withdrawal rate was 18%–19% and relatively even among arms. As our study extracted data from intention-to-treat participants, there was no impact on our outcomes. |
|  | Selective reporting | Assessment: Low risk  Reason: A pre-existing protocol was described and all the results were reported in the pre-specified manner. |
|  | Other sources of bias | Assessment: Low risk  Reason: This study was funded by Boehringer and the conflict of interest may exist, but other sources of bias is not identified. |
|  | Overall | Assessment: Low risk |
| **Frith et al. (2015)** | Random sequence generation | Assessment: Low risk  Reason: The researchers used “Medidata Balance” for randomization. Medidata Balance used a dynamic minimization algorithm to assign treatments. |
|  | Allocation concealment | Assessment: Low risk  Reason: Although the method of allocation concealment was not clearly described, the Medidata Balance was integrated with a system for electronic data capture and clinical data management, which enables allocation concealment for this study. |
|  | Blinding of participants and personnel | Assessment: High risk  Reason: Blinding could be maintained for the investigator, but patients were not fully blinded because the tiotropium and placebo capsules had different shapes. |
|  | Blinding of outcome assessment | Assessment: Low risk  Reason: The unblinded personnel or pharmacist was not involved in any of the study assessments. |
|  | Incomplete outcome data | Assessment: Low risk  Reason: Only 1 missing data was found among intention-to-treat population. |
|  | Selective reporting | Assessment: Low risk  Reason: A pre-existing protocol was described and all the results were reported in the pre-specified manner. |
|  | Other sources of bias | Assessment: Low risk  Reason: This study was funded by Novartis and the conflict of interest exists, but there were no other sources of bias. |
|  | Overall | Assessment: Low risk |
| **Siler et al. (2015); Study 1 (NCT01957163) & Study 2 (NCT02119286)** | Random sequence generation | Assessment: Low risk  Reason: Randomization codes were generated by GSK using a validated computerized system (RandAll v2.13). |
|  | Allocation concealment | Assessment: Unclear risk  Reason: The method of allocation concealment was not described in manuscript. In addition, we could not find an appropriate information on RandAll v2.13. |
|  | Blinding of participants and personnel | Assessment: Low risk  Reason: Umeclidinium and placebo were administered via same device, the ELLIPTA dry powder inhaler. Therefore, treatments were considered double blinded. |
|  | Blinding of outcome assessment | Assessment: Low risk  Reason: The specific method of blinding of outcome assessment was not described. Even if we assume that the investigators who conducted outcome assessment were not blinded, it was difficult for them to make any bias because treatment assignment was blinded during study and our primary outcome was an objective finding (acute exacerbation). Therefore, we concluded that the outcome assessment was unlikely to be biased in this study. |
|  | Incomplete outcome data | Assessment: Low risk  Reason: The withdrawal rate was relatively even among arms. As our study extracted data from intention-to-treat participants, there was no impact on our outcomes. |
|  | Selective reporting | Assessment: Low risk  Reason: A pre-existing protocol was described and all the results were reported in the pre-specified manner. |
|  | Other sources of bias | Assessment: Low risk  Reason: This study was funded by GlaxoSmithKline and the conflict of interest exists, but other sources of bias was not found. |
|  | Overall | Assessment: Low risk |
| **Lee et al. (2016)** | Random sequence generation | Assessment: Unclear risk  Reason: Although this study was designed as a randomized phase IV trial, the method of random sequence generation was not introduced in the article. |
|  | Allocation concealment | Assessment: Unclear risk  Reason: The method of allocation concealment was not described. |
|  | Blinding of participants and personnel | Assessment: High risk  Reason: This study was an open label study and the participants and the study staffs were not blinded to treatment assignment. |
|  | Blinding of outcome assessment | Assessment: High risk  Reason: This study was an open label study and the investigators were not blinded to outcome assessment. It was possible for the investigators to have an influence on the outcome assessment, because they knew treatment assignment during study. |
|  | Incomplete outcome data | Assessment: Low risk  Reason: About 8-11% of patients did not completed the study, and the reasons of withdrawal were relatively balanced between two arms. Therefore, incomplete outcome data is not considered to have a significant bias on our primary and secondary outcomes |
|  | Selective reporting | Assessment: Low risk  Reason: A pre-existing protocol was prospectively registered in database and all the results were reported in the pre-specified manner. |
|  | Other sources of bias | Assessment: Low risk  Reason: This study was funded by AstraZeneca and the conflict of interest exists, but appeared to be free of other sources of bias. |
|  | Overall | Assessment: High risk |
| **Siler et al. (2016); Study 1 (NCT01772134) & Study 2 (NCT01772147)** | Random sequence generation | Assessment: Low risk  Reason: The randomization schedule was generated using a validated computerized system (RandAll version 2.14). |
|  | Allocation concealment | Assessment: Low risk  Reason: The patients were randomized using an Interactive Voice Response System. |
|  | Blinding of participants and personnel | Assessment: Low risk  Reason: Umeclidinium and placebo were administered via identical device, the ELLIPTA dry powder inhaler. Therefore, treatments were considered double blinded. |
|  | Blinding of outcome assessment | Assessment: Low risk  Reason: The specific method of blinding of outcome assessment was not described. Even if we assume that the investigators who conducted outcome assessment were not blinded, it was difficult for them to make any bias because treatment assignment was blinded during study and our primary outcome was an objective finding (acute exacerbation). Therefore, we concluded that the outcome assessment was unlikely to be biased in this study. |
|  | Incomplete outcome data | Assessment: Low risk  Reason: The withdrawal rate was relatively even among arms. As our study extracted data from intention-to-treat participants, there was no impact on our outcomes. |
|  | Selective reporting | Assessment: Low risk  Reason: A pre-existing protocol was described and all the results were reported in the pre-specified manner. |
|  | Other sources of bias | Assessment: Low risk  Reason: This study was funded by GlaxoSmithKline and the conflict of interest exists, but other sources of bias was not found. |
|  | Overall | Assessment: Low risk |
| **Singh et al. (2016)** | Random sequence generation | Low risk of bias. A randomization list was generated by the interactive response technology provider. |
|  | Allocation concealment | Low risk of bias. The patients were randomized using an interactive response technology system. |
|  | Blinding of participants and personnel | Low risk of bias. The two study treatments were provided in matching inhalers, with patients, investigators, site staff, and sponsor personnel masked to treatment assignment for the duration of the study. |
|  | Blinding of outcome assessment | Low risk of bias. Study investigators and site staffs were blinded to treatment assignment. Therefore, it is assumed that the researchers who were involved in outcome assessment were blinded, |
|  | Incomplete outcome data | Low risk of bias. The withdrawal rate was about 12-15% and relatively even among the two treatments. As our study extracted data from intention-to-treat participants, there was no impact on our outcomes. |
|  | Selective reporting | Low risk of bias. A pre-existing protocol was described and all the results were reported in the pre-specified manner. |
|  | Other sources of bias | Low risk of bias. This study was funded by Chiesi Farmaceutici SpA. and the conflict of interest exists, but other sources of bias was not found. |
|  | Overall | Assessment: Low risk |
| **Lipson et al. (2017)** | Random sequence generation | Assessment: Unclear risk  Reason: Although this study was designed as a randomized double-blind trial, the specific method of random sequence generation was not described in the article. |
|  | Allocation concealment | Assessment: Unclear risk  Reason: The method of allocation concealment was not described in manuscript. |
|  | Blinding of participants and personnel | Assessment: Low risk  Reason: Same inhaler devices were used among the treatment groups. Therefore, it was hardly possible to find out which group the patient belonged to. |
|  | Blinding of outcome assessment | Assessment: Low risk  Reason: The specific method of blinding of outcome assessment was not described. Even if we assume that the investigators who conducted outcome assessment were not blinded, it was difficult for them to make any bias because treatment assignment was blinded during study and our primary outcome was an objective finding (acute exacerbation). Therefore, we concluded that the outcome assessment was unlikely to be biased in this study. |
|  | Incomplete outcome data | Assessment: Low risk  Reason: About 94% of patients completed the study, and about 90% completed the study on investigational treatment. In addition, the reasons of withdrawal were relatively balanced between two arms. Therefore, incomplete outcome data is not considered to have a significant bias on our primary and secondary outcomes |
|  | Selective reporting | Assessment: Low risk  Reason: A pre-existing protocol was prospectively registered in database and all the results were reported in the pre-specified manner. |
|  | Other sources of bias | Assessment: Unclear risk  Reason: This study was originally evaluated ITT population for 24 weeks, but EXT population for extended duration of 52 weeks. This kind of design may lead to multiple comparisons bias or selection bias. |
|  | Overall | Assessment: Low risk |
| **Vestbo et al. (2017)** | Random sequence generation | Assessment: Low risk  Reason: Patients were randomized to treatment by investigators contacting an interactive response technology system, which used a randomization list generated by the provider. |
|  | Allocation concealment | Assessment: Low risk  Reason: The patients were randomized using an interactive response technology system. |
|  | Blinding of participants and personnel | Assessment: Low risk  Reason: Patients, investigators, site staff, and funder personnel were masked to treatment assignment for the duration of the study. |
|  | Blinding of outcome assessment | Assessment: Low risk  Reason: The specific method of blinding of outcome assessment was not described. Even if we assume that the investigators who conducted outcome assessment were not blinded, it was difficult for them to make any bias because treatment assignment was blinded during study and our primary outcome was an objective finding (acute exacerbation). Therefore, we concluded that the outcome assessment was unlikely to be biased in this study. |
|  | Incomplete outcome data | Assessment: Low risk  Reason: The withdrawal rate of tiotropium group was about 2 times higher compared to triple group. However, as our study extracted data from intention-to-treat participants, there was no impact on our outcomes. |
|  | Selective reporting | Assessment: Low risk  Reason: A pre-existing protocol was described and all the results were reported in the pre-specified manner. |
|  | Other sources of bias | Assessment: Low risk  Reason: This study was funded by Chiesi Farmaceutici SpA. and the conflict of interest exists, but other sources of bias was not found. |
|  | Overall | Assessment: Low risk |
| **Chapman et al. (2018)** | Random sequence generation | Assessment: Low risk  Reason: As the randomization numbers were generated using a computerized system that conducted the random assignment of patient numbers to randomization numbers and the randomization numbers was not communicated. |
|  | Allocation concealment | Assessment: Low risk  Reason: As the randomization numbers were generated using a computerized system that conducted the random assignment of patient numbers to randomization numbers and the randomization numbers was not communicated. |
|  | Blinding of participants and personnel | Assessment: Low risk  Reason: Study participants and investigator staffs remained blinded to the identity of the treatment from the time of randomization until database lock. |
|  | Blinding of outcome assessment | Assessment: Low risk  Reason: The persons performing the assessments were blinded to the identity of the treatment from the time of randomization until database lock. Randomization data were kept strictly confidential until the time of unblinding, and were not accessible by anyone involved in the study. |
|  | Incomplete outcome data | Assessment: Low risk  Reason: Patients in both groups were followed all the way through to the end of the study. As missing data was balanced between two arms, withdrawal or follow-up loss in surrogates would not have influenced our primary and secondary outcomes. |
|  | Selective reporting | Assessment: Low risk  Reason: A pre-existing protocol was described and all the results were reported in the pre-specified manner. |
|  | Other sources of bias | Assessment: Low risk  Reason: This study was funded by Novartis and the conflict of interest exists, but there were no other sources of bias. |
|  | Overall | Assessment: Low risk |
| **Ferguson et al. (2018)** | Random sequence generation | Assessment: Low risk  Reason: Randomization number for each patient was generated using an interactive web response system. |
|  | Allocation concealment | Assessment: Low risk  Reason: Although the method of allocation concealment was not clearly described, the allocation concealment is considered achieved, because this study performed randomization using an interactive web response system. As patients received a web-based randomization number that matched to an appropriate treatment, allocation was not disclosed to researchers. |
|  | Blinding of participants and personnel | Assessment: Low risk  Reason: Study participants, investigators, and study sponsors were masked to treatment assignment. Open label therapy (Symbicort Turbuhaler) was used, but this treatment arm was designed as an active comparator. |
|  | Blinding of outcome assessment | Assessment: Low risk  Reason: The specific method of blinding of outcome assessment was not described. Even if we assume that the investigators who conducted outcome assessment were not blinded, it was difficult for them to make any bias because treatment assignment was blinded during study and our primary outcome was an objective finding (acute exacerbation). Therefore, we concluded that the outcome assessment was unlikely to be biased in this study. |
|  | Incomplete outcome data | Assessment: Low risk  Reason: Missing data were relatively balanced between four arms. Therefore, withdrawal or follow-up loss of included subjects would not have influenced our primary and secondary outcomes. |
|  | Selective reporting | Assessment: Low risk  Reason: A pre-existing protocol was described and all the results were reported in the pre-specified manner. |
|  | Other sources of bias | Assessment: Low risk  Reason: This study was funded by AstraZeneca and the conflict of interest exists, but appeared to be free of other sources of bias. |
|  | Overall | Assessment: Low risk |
| **Lipson et al. (2018)** | Random sequence generation | Assessment: Low risk  Reason: A randomization code was generated using a validated computerized system. |
|  | Allocation concealment | Assessment: Low risk  Reason: The study used site-based randomization to allocate treatments using an interactive voice response system to maintain allocation concealment. |
|  | Blinding of participants and personnel | Assessment: Low risk  Reason: As the supplied DPIs were identical in appearance, neither the subject nor the investigator knew which one the subject was receiving. |
|  | Blinding of outcome assessment | Assessment: Low risk  Reason: The specific method of blinding of outcome assessment was not described. Even if we assume that the investigators who conducted outcome assessment were not blinded, it was difficult for them to make any bias because treatment assignment was blinded during study and our primary outcome was an objective finding (acute exacerbation). Therefore, we concluded that the outcome assessment was unlikely to be biased in this study. |
|  | Incomplete outcome data | Assessment: Low risk  Reason: The withdrawal rate was 18%–25% and relatively even among arms. As our study extracted data from intention-to-treat participants, there was no impact on our outcomes. |
|  | Selective reporting | Assessment: Low risk  Reason: A pre-existing protocol was described and all the results were reported in the pre-specified manner. |
|  | Other sources of bias | Assessment: Low risk  Reason: This study appears to be free of other sources of bias. |
|  | Overall | Assessment: Low risk |
| **Papi et al. (2018)** | Random sequence generation | Assessment: Low risk  Reason: A randomization list was generated by the interactive response technology provider. |
|  | Allocation concealment | Assessment: Low risk  Reason: Patients were randomly assigned to treatment groups by central randomization for allocation concealment. |
|  | Blinding of participants and personnel | Assessment: Low risk  Reason: Using a double-dummy approach, patients and investigators were masked to treatment assignment for the duration of the study. |
|  | Blinding of outcome assessment | Assessment: Low risk  Reason: The specific method of blinding of outcome assessment was not described. Even if we assume that the investigators who conducted outcome assessment were not blinded, it was difficult for them to make any bias because treatment assignment was blinded during study and our primary outcome was an objective finding (acute exacerbation). Therefore, we concluded that the outcome assessment was unlikely to be biased in this study. |
|  | Incomplete outcome data | Assessment: Low risk  Reason: The withdrawal rate was 12.8%–15.6% and relatively even among arms. As our study extracted data from intention-to-treat participants, there was no impact on our outcomes. |
|  | Selective reporting | Assessment: Low risk  Reason: A pre-existing protocol was described and all the results were reported in the pre-specified manner. |
|  | Other sources of bias | Assessment: Low risk  Reason: This study appears to be free of other sources of bias. |
|  | Overall | Assessment: Low risk |
| **Zhao et al. (2018)** | Random sequence generation | Assessment: Low risk  Reason: The study participants were randomly divided into a control group and an intervention group by using a random number table. |
|  | Allocation concealment | Assessment: Unclear risk  Reason: The method of allocation concealment was not described in manuscript. |
|  | Blinding of participants and personnel | Assessment: Unclear risk  Reason: This study was an open label study and the participants and the study staffs were not blinded to treatment assignment. |
|  | Blinding of outcome assessment | Assessment: High risk  Reason: This study was an open label study and the investigators were not blinded to outcome assessment. As they knew treatment assignment during study, there may be a risk of bias related with outcome assessment. |
|  | Incomplete outcome data | Assessment: High risk  Reason: The patients who dropped out during the study was only one patient in each treatment group. Therefore, the impact on our outcomes was considered trivial. |
|  | Selective reporting | Assessment: Low risk  Reason: A pre-existing protocol was described and all the results were reported in the pre-specified manner. |
|  | Other sources of bias | Assessment: Low risk  Reason: This study appears to be free of other sources of bias. |
|  | Overall | Assessment: Low risk |
| **Ferguson et al. (2020); Study 1 (NCT03478683) & Study 2 (NCT03478696)** | Random sequence generation | Assessment: Low risk  Reason: An Interactive Web Response System was used to randomize patients one to one. |
|  | Allocation concealment | Assessment: Low risk  Reason: Although the method of allocation concealment was not clearly described, the allocation concealment is considered achieved, because this study performed randomization using an interactive web response system. As patients received a web-based randomization number that matched to an appropriate treatment, allocation was not disclosed to researchers. |
|  | Blinding of participants and personnel | Assessment: Low risk  Reason: Double blind was performed using Ellipta, Handihaler, and MDI devices. |
|  | Blinding of outcome assessment | Assessment: Low risk  Reason: The specific method of blinding of outcome assessment was not described. Even if we assume that the investigators who conducted outcome assessment were not blinded, it was difficult for them to make any bias because treatment assignment was blinded during study and our primary outcome was an objective finding (acute exacerbation). Therefore, we concluded that the outcome assessment was unlikely to be biased in this study. |
|  | Incomplete outcome data | Assessment: Low risk  Reason: Missing data were balanced between two arms. In addition, as we extracted data from intention-to-treat participants before initiating treatment, there was no impact on our study outcomes. |
|  | Selective reporting | Assessment: Low risk  Reason: A pre-existing protocol was described and all the results were reported in the pre-specified manner. |
|  | Other sources of bias | Assessment: Low risk  Reason: This study appears to be free of other sources of bias. |
|  | Overall | Assessment: Low risk |
| **Rabe et al. (2020)** | Random sequence generation | Assessment: Unclear risk  Reason: Although this study was designed as a randomized double-blind trial, the specific method of random sequence generation was not described in the article. |
|  | Allocation concealment | Assessment: Unclear risk  Reason: The method of allocation concealment was not described in manuscript. |
|  | Blinding of participants and personnel | Assessment: Low risk  Reason: All treatments were administered through identical metered-dose inhalers. |
|  | Blinding of outcome assessment | Assessment: Low risk  Reason: The specific method of blinding of outcome assessment was not described. Even if we assume that the investigators who conducted outcome assessment were not blinded, it was difficult for them to make any bias because treatment assignment was blinded during study and our primary outcome was an objective finding (acute exacerbation). Therefore, we concluded that the outcome assessment was unlikely to be biased in this study. |
|  | Incomplete outcome data | Assessment: Low risk  Reason: Missing data were balanced between two arms. In addition, as we extracted data from intention-to-treat participants before initiating treatment, there was no impact on our study outcomes. |
|  | Selective reporting | Assessment: Low risk  Reason: A pre-existing protocol was described and all the results were reported in the pre-specified manner. |
|  | Other sources of bias | Assessment: Low risk  Reason: This study appears to be free of other sources of bias. |
|  | Overall | Assessment: Low risk |
| **Bansal et al. (2021)** | Random sequence generation | Assessment: Low risk  Reason: Eligible patients were randomized 1:1 using an Interactive Web Response System. |
|  | Allocation concealment | Assessment: Low risk  Reason: Although the method of allocation concealment was not clearly described, the allocation concealment is considered achieved, because this study performed randomization using an interactive web response system. As patients received a web-based randomization number that matched to an appropriate treatment, allocation was not disclosed to researchers. |
|  | Blinding of participants and personnel | Assessment: Low risk  Reason: A double-dummy design was used to ensure blinding, with each patient given two inhalers (Ellipta and Handihaler) to administer the active medication and placebo, and patients self-administered treatment each day. |
|  | Blinding of outcome assessment | Assessment: Low risk  Reason: All site personnel involved in efficacy and safety assessments were also blinded to assigned treatment during the study |
|  | Incomplete outcome data | Assessment: Low risk  Reason: Missing data were balanced between two arms. In addition, as we extracted data from intention-to-treat participants before initiating treatment, there was no impact on our study outcomes. |
|  | Selective reporting | Assessment: Low risk  Reason: A pre-existing protocol was described and all the results were reported in the pre-specified manner. |
|  | Other sources of bias | Assessment: Low risk  Reason: This study appears to be free of other sources of bias. |
|  | Overall | Assessment: Low risk |
| **Zheng et al. (2021)** | Random sequence generation | Assessment: Low risk  Reason: Patients were assigned to treatment centrally via interactive response technology (IRT), using a balanced block randomization scheme stratified by area that was generated by the IRT provider. |
|  | Allocation concealment | Assessment: Low risk  Reason: The allocation to treatment was conducted centrally via interactive response technology and was not disclosed to researcher. |
|  | Blinding of participants and personnel | Assessment: Low risk  Reason: investigators, and site and sponsor staff were blinded to treatment assignment by a double-dummy design, with matching placebos. |
|  | Blinding of outcome assessment | Assessment: Low risk  Reason: investigators, and site and sponsor staff were blinded to treatment assignment by a double-dummy design, with matching placebos. |
|  | Incomplete outcome data | Assessment: Low risk  Reason: Missing data were balanced between two arms. In addition, as we extracted data from intention-to-treat participants before initiating treatment, there was no impact on our study outcomes. |
|  | Selective reporting | Assessment: Low risk  Reason: A pre-existing protocol was described and all the results were reported in the pre-specified manner. |
|  | Other sources of bias | Assessment: Low risk  Reason: This study appears to be free of other sources of bias. |
|  | Overall | Assessment: Low risk |
| **TRISTAR study (Unpublished)** | Random sequence generation | Assessment: Unclear risk  Reason: The specific method of random sequence generation was not described. |
|  | Allocation concealment | Assessment: Unclear risk  Reason: The specific method of allocation concealment from participants and medical personnel was not described. |
|  | Blinding of participants and personnel | Assessment: High risk  Reason: This study was an open label study and the participants and the study staffs were not blinded to treatment assignment. |
|  | Blinding of outcome assessment | Assessment: Unclear risk  Reason: The specific method of blinding of outcome assessment was not described. |
|  | Incomplete outcome data | Assessment: Low risk  Reason: The withdrawal rate was about 5-6% and relatively even among arms. As our study extracted data from intention-to-treat participants, there was no impact on our outcomes. |
|  | Selective reporting | Assessment: Low risk  Reason: A pre-existing protocol was described and all the results were reported in the pre-specified manner. |
|  | Other sources of bias | Assessment: Unclear risk  Reason: This study was funded by Chiesi Farmaceutici SpA. The conflict of interest may exist and other sources of bias was unclear. |
|  | Overall | Assessment: High risk |

**Supplementary information 4. Assessment of publication bias in the included studies with four or more direct comparisons**

| Total exacerbation | P-value of Egger test | Estimated OR from Trim-and-fill method | Fail-safe N method |
| --- | --- | --- | --- |
| Triple therapy with MD ICS vs. LAMA | 0.575 | - | - |
| Triple therapy with MD ICS vs. MD ICS/LABA | 0.388 | - | - |
| Triple therapy with LD ICS vs. LD ICS/LABA | 0.002 | 1.178 [0.954-1.455] | Fail-safe N: 58 (k=6) |

Publication bias was evaluated when the number of studies was four or more in each direct comparison.

ICS= inhaled corticosteroid; LABA= long-acting beta-agonist; LAMA= long-acting muscarinic antagonist; LD= low-dose; MD= medium-dose; OR= odds ratio


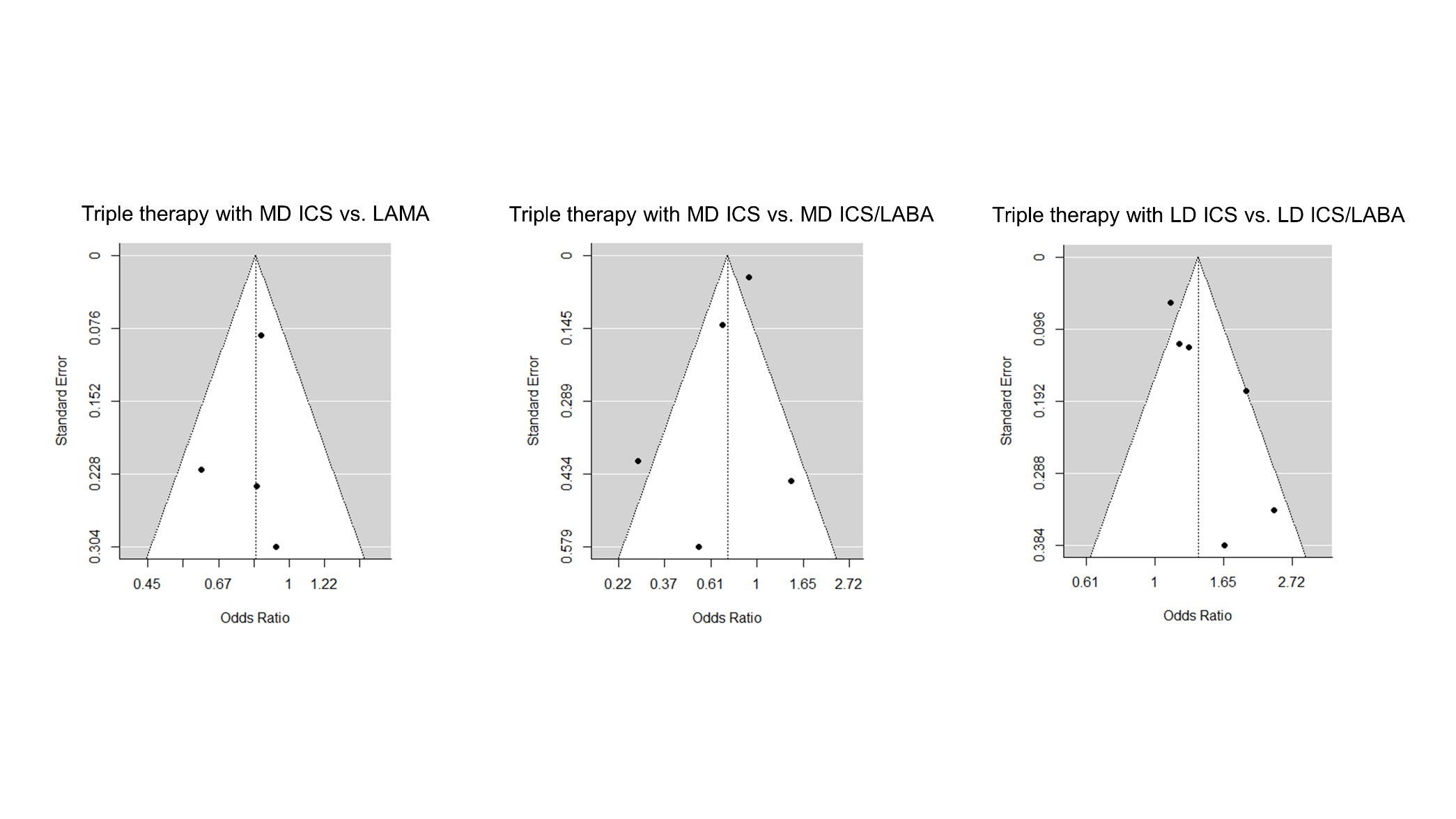


**Supplementary information 5. Estimates of effects and quality ratings for comparison among different inhaled therapies**

| Treatment | Comparator | Median posterior odds ratio (95% CrIs) | Certainty of evidence |
| --- | --- | --- | --- |
| Total exacerbation | | | |
| Triple therapy with HD-ICS | Triple therapy with MD-ICS | 0.81 (0.61-1.14) | Low†‡ |
| Triple therapy with HD-ICS | Triple therapy with LD-ICS | 0.84 (0.62-1.22) | Low†‡ |
| Triple therapy with HD-ICS | LABA/LAMA | 0.70 (0.55-0.93) | High |
| Triple therapy with HD-ICS | HD-ICS/LABA | 0.75 (0.43-1.29) | Moderate* |
| Triple therapy with HD-ICS | MD-ICS/LABA | 0.61 (0.43-0.85) | High |
| Triple therapy with HD-ICS | LD-ICS/LABA | 0.66 (0.45-0.98) | High |
| Triple therapy with HD-ICS | LAMA | 0.65 (0.48-0.93) | High |
| Triple therapy with MD-ICS | Triple therapy with LD-ICS | 1.04 (0.84-1.31) | Moderate* |
| Triple therapy with MD-ICS | LABA/LAMA | 0.86 (0.70-1.05) | High |
| Triple therapy with MD-ICS | HD-ICS/LABA | 0.92 (0.49-1.69) | Low †‡ |
| Triple therapy with MD-ICS | MD-ICS/LABA | 0.75 (0.60-0.89) | High |
| Triple therapy with MD-ICS | LD-ICS/LABA | 0.81 (0.58-1.09) | Low †‡ |
| Triple therapy with MD-ICS | LAMA | 0.81 (0.64-1.01) | Very low*‡§ |
| Triple therapy with LD-ICS | LABA/LAMA | 0.83 (0.65-1.03) | High |
| Triple therapy with LD-ICS | HD-ICS/LABA | 0.89 (0.45-1.63) | Low †‡ |
| Triple therapy with LD-ICS | MD-ICS/LABA | 0.72 (0.53-0.92) | High |
| Triple therapy with LD-ICS | LD-ICS/LABA | 0.78 (0.59-0.98) | Low*‡ |
| Triple therapy with LD-ICS | LAMA | 0.77 (0.58-1.02) | High |
| Total exacerbation, pre-specified subgroup (study duration ≥ 48 weeks) | | | |
| Triple therapy with HD-ICS | Triple therapy with MD-ICS | 0.66 (0.52–0.94) | Low†‡ |
| Triple therapy with HD-ICS | Triple therapy with LD-ICS | 0.66 (0.51–0.94) | Low†‡ |
| Triple therapy with HD-ICS | LABA/LAMA | 0.59 (0.48–0.79) | High |
| Triple therapy with HD-ICS | HD-ICS/LABA | - | - |
| Triple therapy with HD-ICS | MD-ICS/LABA | 0.57 (0.44–0.83) | Low†‡ |
| Triple therapy with HD-ICS | LD-ICS/LABA | 0.61 (0.46–0.91) | Low†‡ |
| Triple therapy with HD-ICS | LAMA | 0.60 (0.45–0.91) | Moderate‡ |
| Triple therapy with MD-ICS | Triple therapy with LD-ICS | 1.00 (0.80–1.23) | Moderate* |
| Triple therapy with MD-ICS | LABA/LAMA | 0.89 (0.74–1.05) | High |
| Triple therapy with MD-ICS | HD-ICS/LABA | - | - |
| Triple therapy with MD-ICS | MD-ICS/LABA | 0.87 (0.71–1.04) | High |
| Triple therapy with MD-ICS | LD-ICS/LABA | 0.93 (0.69–1.22) | Low†‡ |
| Triple therapy with MD-ICS | LAMA | 0.91 (0.72–1.19) | Low*‡ |
| Triple therapy with LD-ICS | LABA/LAMA | 0.89 (0.74–1.07) | High |
| Triple therapy with LD-ICS | HD-ICS/LABA | - | - |
| Triple therapy with LD-ICS | MD-ICS/LABA | 0.86 (0.68–1.09) | Moderate‡ |
| Triple therapy with LD-ICS | LD-ICS/LABA | 0.92 (0.73–1.18) | Very low*†‡ |
| Triple therapy with LD-ICS | LAMA | 0.91 (0.69–1.26) | Moderate‡ |
| Moderate-to-severe exacerbation | | | |
| Triple therapy with HD-ICS | Triple therapy with MD-ICS | 0.73 (0.52–1.09) | Low†‡ |
| Triple therapy with HD-ICS | Triple therapy with LD-ICS | 0.73 (0.53–1.13) | Low†‡ |
| Triple therapy with HD-ICS | LABA/LAMA | 0.63 (0.48–0.88) | High |
| Triple therapy with HD-ICS | HD-ICS/LABA | - | - |
| Triple therapy with HD-ICS | MD-ICS/LABA | 0.57 (0.39–0.85) | Low†‡ |
| Triple therapy with HD-ICS | LD-ICS/LABA | 0.63 (0.42–0.98) | Low†‡ |
| Triple therapy with HD-ICS | LAMA | 0.59 (0.38–0.96) | Low†‡ |
| Triple therapy with MD-ICS | Triple therapy with LD-ICS | 1.01 (0.79–1.33) | Moderate‡ |
| Triple therapy with MD-ICS | LABA/LAMA | 0.87 (0.70–1.07) | High |
| Triple therapy with MD-ICS | HD-ICS/LABA | - | - |
| Triple therapy with MD-ICS | MD-ICS/LABA | 0.79 (0.63–0.95) | High |
| Triple therapy with MD-ICS | LD-ICS/LABA | 0.87 (0.61–1.19) | Low†‡ |
| Triple therapy with MD-ICS | LAMA | 0.81 (0.60–1.08) | Moderate* |
| Triple therapy with LD-ICS | LABA/LAMA | 0.86 (0.67–1.07) | High |
| Triple therapy with LD-ICS | HD-ICS/LABA | - | - |
| Triple therapy with LD-ICS | MD-ICS/LABA | 0.78 (0.57–0.99) | Low‡§ |
| Triple therapy with LD-ICS | LD-ICS/LABA | 0.86 (0.63–1.08) | High |
| Triple therapy with LD-ICS | LAMA | 0.80 (0.56–1.12) | Moderate‡ |
| Moderate-to-severe exacerbation, pre-specified subgroup (FEV_1_ < 65%) | | | |
| Triple therapy with HD-ICS | Triple therapy with MD-ICS | 0.60 (0.37–0.98) | Low†‡ |
| Triple therapy with HD-ICS | Triple therapy with LD-ICS | 0.61 (0.39–1.02) | Low†‡ |
| Triple therapy with HD-ICS | LABA/LAMA | 0.54 (0.35–0.81) | Moderate‡ |
| Triple therapy with HD-ICS | HD-ICS/LABA | - | - |
| Triple therapy with HD-ICS | MD-ICS/LABA | 0.48 (0.28–0.77) | Low†‡ |
| Triple therapy with HD-ICS | LD-ICS/LABA | 0.53 (0.31–0.89) | Low†‡ |
| Triple therapy with HD-ICS | LAMA | 0.48 (0.26–0.83) | Low†‡ |
| Triple therapy with MD-ICS | Triple therapy with LD-ICS | 1.02 (0.79–1.38) | Moderate‡ |
| Triple therapy with MD-ICS | LABA/LAMA | 0.90 (0.69–1.15) | High |
| Triple therapy with MD-ICS | HD-ICS/LABA | - | - |
| Triple therapy with MD-ICS | MD-ICS/LABA | 0.81 (0.60–0.99) | Moderate§ |
| Triple therapy with MD-ICS | LD-ICS/LABA | 0.89 (0.60–1.24) | Low†‡ |
| Triple therapy with MD-ICS | LAMA | 0.80 (0.54–1.10) | Low*‡ |
| Triple therapy with LD-ICS | LABA/LAMA | 0.88 (0.66–1.11) | High |
| Triple therapy with LD-ICS | HD-ICS/LABA | - | - |
| Triple therapy with LD-ICS | MD-ICS/LABA | 0.79 (0.54–1.02) | Moderate‡ |
| Triple therapy with LD-ICS | LD-ICS/LABA | 0.87 (0.62–1.11) | High |
| Triple therapy with LD-ICS | LAMA | 0.78 (0.51–1.10) | Moderate‡ |
| Moderate-to-severe exacerbation, pre-specified subgroup (at least 1 exacerbation event in the past year) | | | |
| Triple therapy with HD-ICS | Triple therapy with MD-ICS | 0.60 (0.36–0.999) | Very low†‡§ |
| Triple therapy with HD-ICS | Triple therapy with LD-ICS | 0.61 (0.38–1.04) | Low†‡ |
| Triple therapy with HD-ICS | LABA/LAMA | 0.54 (0.35–0.83) | Moderate‡ |
| Triple therapy with HD-ICS | HD-ICS/LABA | - | - |
| Triple therapy with HD-ICS | MD-ICS/LABA | 0.48 (0.27–0.80) | Low†‡ |
| Triple therapy with HD-ICS | LD-ICS/LABA | 0.53 (0.30–0.90) | Low†‡ |
| Triple therapy with HD-ICS | LAMA | 0.48 (0.26–0.84) | Low†‡ |
| Triple therapy with MD-ICS | Triple therapy with LD-ICS | 1.03 (0.78–1.40) | Moderate‡ |
| Triple therapy with MD-ICS | LABA/LAMA | 0.90 (0.68–1.18) | High |
| Triple therapy with MD-ICS | HD-ICS/LABA | - | - |
| Triple therapy with MD-ICS | MD-ICS/LABA | 0.81 (0.60–0.996) | Moderate§ |
| Triple therapy with MD-ICS | LD-ICS/LABA | 0.89 (0.59–1.27) | Low†‡ |
| Triple therapy with MD-ICS | LAMA | 0.80 (0.54–1.10) | Low*‡ |
| Triple therapy with LD-ICS | LABA/LAMA | 0.88 (0.66–1.12) | High |
| Triple therapy with LD-ICS | HD-ICS/LABA | - | - |
| Triple therapy with LD-ICS | MD-ICS/LABA | 0.79 (0.53–1.03) | Moderate‡ |
| Triple therapy with LD-ICS | LD-ICS/LABA | 0.87 (0.62–1.11) | High |
| Triple therapy with LD-ICS | LAMA | 0.78 (0.50–1.10) | Moderate‡ |
| All–cause mortality | | | |
| Triple therapy with HD-ICS | Triple therapy with MD-ICS | 1.53 (0.86–2.88) | Low†‡ |
| Triple therapy with HD-ICS | Triple therapy with LD-ICS | 1.26 (0.71–2.39) | Low†‡ |
| Triple therapy with HD-ICS | LABA/LAMA | 0.96 (0.60–1.60) | High |
| Triple therapy with HD-ICS | HD-ICS/LABA | 0.41 (0.01–11.61) | Low*§ |
| Triple therapy with HD-ICS | MD-ICS/LABA | 1.38 (0.73–2.77) | Low†‡ |
| Triple therapy with HD-ICS | LD-ICS/LABA | 1.02 (0.50–1.91) | Low†‡ |
| Triple therapy with HD-ICS | LAMA | 1.07 (0.52–2.32) | High |
| Triple therapy with MD-ICS | Triple therapy with LD-ICS | 0.82 (0.53–1.26) | Moderate* |
| Triple therapy with MD-ICS | LABA/LAMA | 0.62 (0.42–0.92) | High |
| Triple therapy with MD-ICS | HD-ICS/LABA | 0.27 (0.01–7.69) | Very low†‡§ |
| Triple therapy with MD-ICS | MD-ICS/LABA | 0.90 (0.59–1.39) | Moderate‡ |
| Triple therapy with MD-ICS | LD-ICS/LABA | 0.67 (0.35–1.08) | Low†‡ |
| Triple therapy with MD-ICS | LAMA | 0.70 (0.42–1.20) | Moderate* |
| Triple therapy with LD-ICS | LABA/LAMA | 0.76 (0.53–1.09) | High |
| Triple therapy with LD-ICS | HD-ICS/LABA | 0.32 (0.01–9.64) | Very low†‡§ |
| Triple therapy with LD-ICS | MD-ICS/LABA | 1.10 (0.68–1.79) | Moderate‡ |
| Triple therapy with LD-ICS | LD-ICS/LABA | 0.82 (0.46–1.18) | Moderate* |
| Triple therapy with LD-ICS | LAMA | 0.85 (0.46–1.62) | Low†‡ |
| Serious adverse event | | | |
| Triple therapy with HD-ICS | Triple therapy with MD-ICS | 1.17 (0.89–1.65) | Low†‡ |
| Triple therapy with HD-ICS | Triple therapy with LD-ICS | 1.01 (0.75–1.37) | Low†‡ |
| Triple therapy with HD-ICS | LABA/LAMA | 0.98 (0.77–1.27) | High |
| Triple therapy with HD-ICS | HD-ICS/LABA | 1.24 (0.64–2.45) | Moderate* |
| Triple therapy with HD-ICS | MD-ICS/LABA | 1.00 (0.73–1.37) | Low†‡ |
| Triple therapy with HD-ICS | LD-ICS/LABA | 1.03 (0.71–1.40) | Low†‡ |
| Triple therapy with HD-ICS | LAMA | 0.96 (0.67–1.41) | High |
| Triple therapy with MD-ICS | Triple therapy with LD-ICS | 0.86 (0.70–1.02) | Moderate* |
| Triple therapy with MD-ICS | LABA/LAMA | 0.84 (0.68–0.98) | High |
| Triple therapy with MD-ICS | HD-ICS/LABA | 1.06 (0.51–2.22) | Low†‡ |
| Triple therapy with MD-ICS | MD-ICS/LABA | 0.85 (0.69–0.99) | Low‡§ |
| Triple therapy with MD-ICS | LD-ICS/LABA | 0.89 (0.63–1.09) | Low†‡ |
| Triple therapy with MD-ICS | LAMA | 0.81 (0.64–1.04) | Low*‡ |
| Triple therapy with LD-ICS | LABA/LAMA | 0.97 (0.82–1.17) | High |
| Triple therapy with LD-ICS | HD-ICS/LABA | 1.23 (0.60–2.60) | Low*‡ |
| Triple therapy with LD-ICS | MD-ICS/LABA | 0.98 (0.79–1.21) | Moderate‡ |
| Triple therapy with LD-ICS | LD-ICS/LABA | 1.03 (0.79–1.22) | Moderate* |
| Triple therapy with LD-ICS | LAMA | 0.95 (0.71–1.29) | Moderate‡ |
| Serious cardiac adverse event | | | |
| Triple therapy with HD-ICS | Triple therapy with MD-ICS | 1.20 (0.64–2.23) | Low†‡ |
| Triple therapy with HD-ICS | Triple therapy with LD-ICS | 1.13 (0.59–2.20) | Low†‡ |
| Triple therapy with HD-ICS | LABA/LAMA | 0.83 (0.48–1.41) | High |
| Triple therapy with HD-ICS | HD-ICS/LABA | 1.21 (0.35–5.18) | Moderate* |
| Triple therapy with HD-ICS | MD-ICS/LABA | 1.24 (0.60–2.42) | Low†‡ |
| Triple therapy with HD-ICS | LD-ICS/LABA | 0.75 (0.35–1.61) | Low†‡ |
| Triple therapy with HD-ICS | LAMA | 0.97 (0.43–2.14) | Moderate‡ |
| Triple therapy with MD-ICS | Triple therapy with LD-ICS | 0.94 (0.64–1.38) | Moderate* |
| Triple therapy with MD-ICS | LABA/LAMA | 0.69 (0.49–0.96) | High |
| Triple therapy with MD-ICS | HD-ICS/LABA | 1.01 (0.25–4.61) | Low†‡ |
| Triple therapy with MD-ICS | MD-ICS/LABA | 1.04 (0.67–1.52) | Moderate‡ |
| Triple therapy with MD-ICS | LD-ICS/LABA | 0.62 (0.36–1.10) | Low†‡ |
| Triple therapy with MD-ICS | LAMA | 0.81 (0.44–1.44) | Low*‡ |
| Triple therapy with LD-ICS | LABA/LAMA | 0.73 (0.49–1.07) | High |
| Triple therapy with LD-ICS | HD-ICS/LABA | 1.08 (0.25–5.17) | Low†‡ |
| Triple therapy with LD-ICS | MD-ICS/LABA | 1.10 (0.67–1.73) | Moderate‡ |
| Triple therapy with LD-ICS | LD-ICS/LABA | 0.66 (0.42–1.03) | Low*‡ |
| Triple therapy with LD-ICS | LAMA | 0.86 (0.43–1.70) | Moderate‡ |
| Pneumonia | | | |
| Triple therapy with HD-ICS | Triple therapy with MD-ICS | 0.78 (0.45–1.50) | Low†‡ |
| Triple therapy with HD-ICS | Triple therapy with LD-ICS | 0.77 (0.45–1.45) | Low†‡ |
| Triple therapy with HD-ICS | LABA/LAMA | 1.14 (0.73–1.91) | High |
| Triple therapy with HD-ICS | HD-ICS/LABA | 0.48 (0.04–5.15) | Low*‡ |
| Triple therapy with HD-ICS | MD-ICS/LABA | 0.70 (0.41–1.42) | Low†‡ |
| Triple therapy with HD-ICS | LD-ICS/LABA | 0.88 (0.50–1.85) | Low†‡ |
| Triple therapy with HD-ICS | LAMA | 1.07 (0.52–2.35) | Moderate‡ |
| Triple therapy with MD-ICS | Triple therapy with LD-ICS | 0.98 (0.69–1.35) | Moderate* |
| Triple therapy with MD-ICS | LABA/LAMA | 1.47 (1.004–2.01) | Moderate§ |
| Triple therapy with MD-ICS | HD-ICS/LABA | 0.61 (0.04–6.80) | Low†‡ |
| Triple therapy with MD-ICS | MD-ICS/LABA | 0.91 (0.66–1.26) | Moderate‡ |
| Triple therapy with MD-ICS | LD-ICS/LABA | 1.12 (0.72–1.82) | Low†‡ |
| Triple therapy with MD-ICS | LAMA | 1.35 (0.81–2.27) | Low*‡ |
| Triple therapy with LD-ICS | LABA/LAMA | 1.50 (1.06–2.04) | High |
| Triple therapy with LD-ICS | HD-ICS/LABA | 0.63 (0.05–7.08) | Low†‡ |
| Triple therapy with LD-ICS | MD-ICS/LABA | 0.93 (0.65–1.41) | Low†‡ |
| Triple therapy with LD-ICS | LD-ICS/LABA | 1.13 (0.83–1.76) | Low*‡ |
| Triple therapy with LD-ICS | LAMA | 1.39 (0.78–2.53) | Moderate‡ |
| Treatment | **Comparator** | **Median posterior mean difference (95% CrIs)** | **Certainty of evidence** |
| Change of trough FEV_1_, ml |  |  |  |
| Triple therapy with HD-ICS | Triple therapy with MD-ICS | -25.6 (-93.9–43.1) | Low†‡ |
| Triple therapy with HD-ICS | Triple therapy with LD-ICS | -52.9 (-123.2–17.4) | Low†‡ |
| Triple therapy with HD-ICS | LABA/LAMA | 6.4 (-71.1–83) | Low†‡ |
| Triple therapy with HD-ICS | HD-ICS/LABA | 46.2 (-15.3–106.3) | Moderate‡ |
| Triple therapy with HD-ICS | MD-ICS/LABA | 41.1 (-33.8–116.6) | Low†‡ |
| Triple therapy with HD-ICS | LD-ICS/LABA | 73.3 (-0.8–149.6) | Low†‡ |
| Triple therapy with HD-ICS | LAMA | 44.7 (-15.2–105.1) | Moderate‡ |
| Triple therapy with MD-ICS | Triple therapy with LD-ICS | -27.5 (-54.6–0.2) | Moderate* |
| Triple therapy with MD-ICS | LABA/LAMA | 31.8 (-4.8–70.2) | Moderate‡ |
| Triple therapy with MD-ICS | HD-ICS/LABA | 71.5 (1.9–140.6) | Low†‡ |
| Triple therapy with MD-ICS | MD-ICS/LABA | 66.6 (34.4–98.8) | Moderate‡ |
| Triple therapy with MD-ICS | LD-ICS/LABA | 98.9 (63.5–138.3) | Low†‡ |
| Triple therapy with MD-ICS | LAMA | 70.3 (37.8–102.8) | Low*‡ |
| Triple therapy with LD-ICS | LABA/LAMA | 59.5 (22.8–96.2) | Moderate‡ |
| Triple therapy with LD-ICS | HD-ICS/LABA | 99.3 (27.8–168.6) | Low†‡ |
| Triple therapy with LD-ICS | MD-ICS/LABA | 94.1 (53.5–134.6) | Low†‡ |
| Triple therapy with LD-ICS | LD-ICS/LABA | 126.6 (100.6–155.5) | Moderate* |
| Triple therapy with LD-ICS | LAMA | 97.8 (60–134.8) | Moderate‡ |
| Change of SGRQ score |  |  |  |
| Triple therapy with HD-ICS | Triple therapy with MD-ICS | 0.3 (-1.6–2.1) | Low†‡ |
| Triple therapy with HD-ICS | Triple therapy with LD-ICS | 0.3 (-1.6–2) | Low†‡ |
| Triple therapy with HD-ICS | LABA/LAMA | -1.3 (-2.9–0.1) | High |
| Triple therapy with HD-ICS | HD-ICS/LABA | -2.7 (-4.9–-0.6) | Low*‡ |
| Triple therapy with HD-ICS | MD-ICS/LABA | -1.2 (-3.2–0.6) | Low†‡ |
| Triple therapy with HD-ICS | LD-ICS/LABA | -1 (-2.8–1.2) | Low†‡ |
| Triple therapy with HD-ICS | LAMA | -2.9 (-5.3–-0.6) | Low†‡ |
| Triple therapy with MD-ICS | Triple therapy with LD-ICS | 0 (-0.9–0.9) | Moderate* |
| Triple therapy with MD-ICS | LABA/LAMA | -1.6 (-2.7–-0.5) | High |
| Triple therapy with MD-ICS | HD-ICS/LABA | -3 (-5.8–-0.2) | Low†‡ |
| Triple therapy with MD-ICS | MD-ICS/LABA | -1.5 (-2.5–-0.6) | Moderate‡ |
| Triple therapy with MD-ICS | LD-ICS/LABA | -1.3 (-2.4–0.2) | Low†‡ |
| Triple therapy with MD-ICS | LAMA | -3.1 (-4.8–-1.6) | Low*‡ |
| Triple therapy with LD-ICS | LABA/LAMA | -1.6 (-2.6–-0.5) | High |
| Triple therapy with LD-ICS | HD-ICS/LABA | -3 (-5.8–-0.2) | Low†‡ |
| Triple therapy with LD-ICS | MD-ICS/LABA | -1.5 (-2.7–-0.4) | Moderate‡ |
| Triple therapy with LD-ICS | LD-ICS/LABA | -1.3 (-2–-0.03) | Very low*‡§ |
| Triple therapy with LD-ICS | LAMA | -3.1 (-4.8–-1.6) | Moderate‡ |

* Risk of bias. † Inconsistency (including the case where consistency assumption could not be evaluated). ‡ Indirectness (because of questionable comparability or intransitivity). § Imprecision (§§ Severe imprecision). ‖ Publication bias.

CrI= credible interval; HD= high-dose; ICS= inhaled corticosteroid; LABA= long-acting beta-agonist; LAMA= long-acting muscarinic antagonist; LD= low-dose; MD= medium-dose

**Supplementary information 6.** **Sensitivity analyses to evaluate the risk of total exacerbation associated with different inhaled therapies**

|  | Triple therapy with HD ICS | Triple therapy with MD ICS | Triple therapy with LD ICS | LABA/LAMA | HD ICS/LABA | MD ICS/LABA | LD ICS/LABA | LAMA |
| --- | --- | --- | --- | --- | --- | --- | --- | --- |
| FEV_1_ <65%, (14 studies, 32,981 patients) | | | | | | | | |
| Rank | 1 | 3 | 2 | 5 | 6 | 8 | 4 | 7 |
| SUCRA, % | 96.54 | 70.23 | 76.04 | 40.19 | 27.34 | 23.54 | 40.23 | 25.89 |
| NMA estimate OR (95% CrI) | | | | | | | | |
| Triple therapy with HD ICS | 1 |  |  |  |  |  |  |  |
| Triple therapy with MD ICS | 0.7 (0.51–1.04) | 1 |  |  |  |  |  |  |
| Triple therapy with LD ICS | 0.72 (0.53–1.09) | 1.03 (0.84–1.3) | 1 |  |  |  |  |  |
| LABA/LAMA | 0.61 (0.47–0.85)* | 0.87 (0.7–1.06) | 0.85 (0.66–1.02) | 1 |  |  |  |  |
| HD ICS/LABA | 0.42 (0.1–1.89) | 0.6 (0.14–2.71) | 0.58 (0.13–2.64) | 0.69 (0.16–3.15) | 1 |  |  |  |
| MD ICS/LABA | 0.56 (0.39–0.84)* | 0.8 (0.62–0.97)* | 0.78 (0.56–0.98)* | 0.92 (0.69–1.18) | 1.32 (0.29–5.84) | 1 |  |  |
| LD ICS/LABA | 0.61 (0.42–0.94)* | 0.87 (0.63–1.16) | 0.85 (0.63–1.05) | 1 (0.75–1.31) | 1.45 (0.31–6.31) | 1.09 (0.78–1.56) | 1 |  |
| LAMA | 0.56 (0.4–0.83)* | 0.81 (0.63–0.999)* | 0.79 (0.58–1.004) | 0.93 (0.71–1.2) | 1.33 (0.3–5.69) | 1.01 (0.75–1.37) | 0.93 (0.66–1.32) | 1 |
| At least 1 exacerbation event in the past year (12 studies, 32,436 patients) | | | | | | | | |
| Rank | 1 | 2 | 3 | 5 | - | 7 | 4 | 6 |
| SUCRA, % | 96.68 | 75.65 | 69.96 | 34.67 | - | 16.82 | 34.89 | 21.3 |
| NMA estimate OR (95% CrI) | | | | | | | | |
| Triple therapy with HD ICS | 1 |  |  |  |  |  |  |  |
| Triple therapy with MD ICS | 0.72 (0.53–1.13) | 1 |  |  |  |  |  |  |
| Triple therapy with LD ICS | 0.73 (0.54–1.18) | 1.03 (0.82–1.3) | 1 |  |  |  |  |  |
| LABA/LAMA | 0.62 (0.47–0.89)* | 0.87 (0.67–1.06) | 0.84 (0.64–1.03) | 1 |  |  |  |  |
| HD ICS/LABA |  |  |  | - | - |  |  |  |
| MD ICS/LABA | 0.57 (0.39–0.89)* | 0.8 (0.61–0.97)* | 0.78 (0.56–0.98)* | 0.92 (0.69–1.2) | - | 1 |  |  |
| LD ICS/LABA | 0.62 (0.42–1.0003) | 0.86 (0.6–1.17) | 0.84 (0.62–1.06) | 1 (0.73–1.35) | - | 1.08 (0.75–1.57) | 1 |  |
| LAMA | 0.58 (0.41–0.9)* | 0.81 (0.61–1.03) | 0.78 (0.57–1.04) | 0.93 (0.7–1.26) | - | 1.01 (0.74–1.43) | 0.93 (0.65–1.37) | 1 |
| mMRC ≥2 or CAT ≥10 (14 studies, 32,915 patients) | | | | | | | | |
| Rank |  | 2 | 1 |  | 3 | 6 | 4 | 5 |
| SUCRA, % |  | 79.97 | 94.27 |  | 48.1 | 16.45 | 41.14 | 20.07 |
| NMA estimate OR (95% CrI) | | | | | | | | |
| Triple therapy with HD ICS | - |  |  |  |  |  |  |  |
| Triple therapy with MD ICS | - | 1 |  |  |  |  |  |  |
| Triple therapy with LD ICS | - | 1.05 (0.91–1.24) | 1 |  |  |  |  |  |
| LABA/LAMA | - | 0.92 (0.79–1.05) | 0.87 (0.73–0.99)* | 1 |  |  |  |  |
| HD ICS/LABA | - | - | - | - | - |  |  |  |
| MD ICS/LABA | - | 0.83 (0.69–0.95)* | 0.79 (0.62–0.92)* | 0.91 (0.75–1.05) | - | 1 |  |  |
| LD ICS/LABA | - | 0.9 (0.7–1.07) | 0.86 (0.68–0.98)* | 0.98 (0.78–1.16) | - | 1.08 (0.85–1.34) | 1 |  |
| LAMA | - | 0.83 (0.66–1.02) | 0.79 (0.6–0.99)* | 0.91 (0.7–1.16) | - | 1 (0.77–1.32) | 0.92 (0.7–1.27) | 1 |
| Mean blood eosinophil count ≥200/uL (3 studies, 5,589 patients) | | | | | | | |  |
| Rank | - | 1 | - | 2 | - | 4 | - | 3 |
| SUCRA, % | - | 82.92 | - | 54.5 | - | 29.11 | - | 33.47 |
| NMA estimate OR (95% CrI) | | | | | | | | |
| Triple therapy with HD ICS | - |  |  |  |  |  |  |  |
| Triple therapy with MD ICS | - | 1 |  |  |  |  |  |  |
| Triple therapy with LD ICS | - | - | - |  |  |  |  |  |
| LABA/LAMA | - | 0.93 (0.68–1.25) | - | 1 |  |  |  |  |
| HD ICS/LABA | - | - | - | - | - |  |  |  |
| MD ICS/LABA | - | 0.83 (0.61–1.14) | - | 0.90 (0.59–1.38) | - | 1 |  |  |
| LD ICS/LABA | - | - | - | - | - | - | - |  |
| LAMA | - | 0.86 (0.65–1.13) | - | 0.93 (0.62–1.39) | - | 0.97 (0.65–1.47) | - | 1 |
| Study duration <24 weeks (8 studies, 4,697 patients) | | | | | | | | |
| Rank | 1 | 3 | 2 | - | 4 | 5 | 7 | 6 |
| SUCRA, % | 72.36 | 62.04 | 62.35 | - | 49.37 | 38.93 | 29.76 | 35.19 |
| NMA estimate OR (95% CrI) | | | | | | | | |
| Triple therapy with HD ICS | 1 |  |  |  |  |  |  |  |
| Triple therapy with MD ICS | 0.69 (0.04–9.98) | 1 |  |  |  |  |  |  |
| Triple therapy with LD ICS | 0.7 (0.04–10.07) | 1.03 (0.09–10.93) | 1 |  |  |  |  |  |
| LABA/LAMA | - | - | - | - |  |  |  |  |
| HD ICS/LABA | 0.6 (0.12–2.22) | 0.85 (0.06–12.95) | 0.85 (0.06–13.12) | - | 1 |  |  |  |
| MD ICS/LABA | 0.35 (0.01–6.9) | 0.5 (0.14–1.79) | 0.49 (0.03–7.59) | - | 0.59 (0.03–11.78) | 1 |  |  |
| LD ICS/LABA | 0.43 (0.02–8.17) | 0.64 (0.04–9.6) | 0.63 (0.17–2.38) | - | 0.74 (0.04–14.41) | 1.28 (0.06–24.39) | 1 |  |
| LAMA | 0.41 (0.04–3.48) | 0.6 (0.11–3.38) | 0.6 (0.1–3.32) | - | 0.71 (0.08–6.19) | 1.21 (0.15–10) | 0.97 (0.11–7.86) | 1 |
| Study duration ≥24 weeks (15 studies, 34,985 patients) | | | | | | | | |
| Rank | 1 | 3 | 2 | 5 | - | 6 | 7 | 4 |
| SUCRA, % | 97 | 68.58 | 72.76 | 34.62 | - | 27.44 | 9.88 | 39.71 |
| NMA estimate OR (95% CrI) | | | | | | | | |
| Triple therapy with HD ICS | 1 |  |  |  |  |  |  |  |
| Triple therapy with MD ICS | 0.78 (0.6–1.06) | 1 |  |  |  |  |  |  |
| Triple therapy with LD ICS | 0.79 (0.6–1.11) | 1.02 (0.83–1.26) | 1 |  |  |  |  |  |
| LABA/LAMA | 0.69 (0.55–0.89)* | 0.88 (0.74–1.05) | 0.87 (0.7–1.05) | 1 |  |  |  |  |
| HD ICS/LABA | - | - | - | - | - |  |  |  |
| MD ICS/LABA | 0.61 (0.46–0.84)* | 0.79 (0.64–0.93)* | 0.78 (0.59–0.97)* | 0.9 (0.71–1.08) | - | 1 |  |  |
| LD ICS/LABA | 0.66 (0.47–0.94)* | 0.86 (0.62–1.1) | 0.84 (0.64–1.03) | 0.97 (0.72–1.23) | - | 1.08 (0.79–1.46) | 1 |  |
| LAMA | 0.7 (0.51–1.01) | 0.9 (0.71–1.14) | 0.88 (0.65–1.19) | 1.02 (0.78–1.35) | - | 1.14 (0.86–1.56) | 1.05 (0.76–1.55) | 1 |
| Study duration <48 weeks (16 studies, 12,295 patients) | | | | | | | | |
| Rank | 3 | 2 | 1 | 4 | 5 | 8 | 6 | 7 |
| SUCRA, % | 71.14 | 72.22 | 85.42 | 51.24 | 38.96 | 14.19 | 35.67 | 31.18 |
| NMA estimate OR (95% CrI) | | | | | | | | |
| Triple therapy with HD ICS | 1 |  |  |  |  |  |  |  |
| Triple therapy with MD ICS | 1 (0.46–1.97) | 1 |  |  |  |  |  |  |
| Triple therapy with LD ICS | 1.15 (0.45–2.62) | 1.15 (0.7–1.93) | 1 |  |  |  |  |  |
| LABA/LAMA | 0.85 (0.45–1.48) | 0.86 (0.51–1.48) | 0.74 (0.37–1.57) | 1 |  |  |  |  |
| HD ICS/LABA | 0.75 (0.38–1.42) | 0.76 (0.31–1.86) | 0.66 (0.24–1.81) | 0.88 (0.39–2.02) | 1 |  |  |  |
| MD ICS/LABA | 0.62 (0.27–1.2) | 0.62 (0.42–0.86)* | 0.54 (0.28–0.96)* | 0.73 (0.39–1.18) | 0.82 (0.31–1.98) | 1 |  |  |
| LD ICS/LABA | 0.74 (0.26–1.84) | 0.75 (0.38–1.42) | 0.65 (0.42–0.96)* | 0.87 (0.36–1.97) | 0.98 (0.32–2.89) | 1.21 (0.57–2.61) | 1 |  |
| LAMA | 0.72 (0.32–1.5) | 0.73 (0.49–1.07) | 0.63 (0.36–1.06) | 0.85 (0.44–1.56) | 0.95 (0.37–2.42) | 1.17 (0.71–2.04) | 0.97 (0.49–1.95) | 1 |
| Study duration ≥48 weeks (7 studies, 27,387 patients) | | | | | | | | |
| Rank | 1 | 3 | 2 | 6 | - | 7 | 4 | 5 |
| SUCRA, % | 98.88 | 66.13 | 67.03 | 26.01 | - | 17.68 | 39.83 | 34.45 |
| NMA estimate OR (95% CrI) | | | | | | | | |
| Triple therapy with HD ICS | 1 |  |  |  |  |  |  |  |
| Triple therapy with MD ICS | 0.66 (0.52–0.94)* | 1 |  |  |  |  |  |  |
| Triple therapy with LD ICS | 0.66 (0.51–0.94)* | 1 (0.8–1.23) | 1 |  |  |  |  |  |
| LABA/LAMA | 0.59 (0.48–0.79)* | 0.89 (0.74–1.05) | 0.89 (0.74–1.07) | 1 |  |  |  |  |
| HD ICS/LABA | - | - | - | - | - |  |  |  |
| MD ICS/LABA | 0.57 (0.44–0.83)* | 0.87 (0.71–1.04) | 0.86 (0.68–1.09) | 0.97 (0.78–1.21) | - | 1 |  |  |
| LD ICS/LABA | 0.61 (0.46–0.91)* | 0.93 (0.69–1.22) | 0.92 (0.73–1.18) | 1.04 (0.81–1.33) | - | 1.07 (0.79–1.45) | 1 |  |
| LAMA | 0.6 (0.45–0.91)* | 0.91 (0.72–1.19) | 0.91 (0.69–1.26) | 1.02 (0.8–1.37) | - | 1.05 (0.8–1.46) | 0.98 (0.71–1.45) | 1 |

CrI= credible interval; HD= high-dose; ICS= inhaled corticosteroid; LABA= long-acting beta-agonist; LAMA= long-acting muscarinic antagonist; LD= low-dose; MD= medium-dose; NMA= network meta-analysis; OR= odds ratio; SUCRA= surface under the cumulative ranking curve

Median odds ratio with 95% credible interval was calculated as a row to column ratio. If the OR is significantly lower than 1, the drug in the left row is more beneficial than the other drug in the upper column.

* indicates that the posterior probability is either less than 0.025 or more than 0.975, which is considered statistically significant.

**Supplementary information 7. Sensitivity analyses to evaluate the risk of moderate-to-severe exacerbation associated with different inhaled therapies**

|  | Triple therapy with HD ICS | Triple therapy with MD ICS | Triple therapy with LD ICS | LABA/LAMA | HD ICS/LABA | MD ICS/LABA | LD ICS/LABA | LAMA |
| --- | --- | --- | --- | --- | --- | --- | --- | --- |
| FEV1 <65%, (9 studies, 30,254 patients) | | | | | | | | |
| Rank | 1 | 3 | 2 | 4 | - | 7 | 5 | 6 |
| SUCRA, % | 98.6 | 66.8 | 72.73 | 39.31 | - | 16.23 | 37.66 | 18.68 |
| NMA estimate OR (95% CrI) | | | | | | | | |
| Triple therapy with HD ICS | 1 |  |  |  |  |  |  |  |
| Triple therapy with MD ICS | 0.6 (0.37–0.98)* | 1 |  |  |  |  |  |  |
| Triple therapy with LD ICS | 0.61 (0.39–1.02) | 1.02 (0.79–1.38) | 1 |  |  |  |  |  |
| LABA/LAMA | 0.54 (0.35–0.81) | 0.9 (0.69–1.15) | 0.88 (0.66–1.11) | 1 |  |  |  |  |
| HD ICS/LABA | - | - | - | - | - |  |  |  |
| MD ICS/LABA | 0.48 (0.28–0.77)* | 0.81 (0.6–0.99)* | 0.79 (0.54–1.02) | 0.9 (0.64–1.18) | - | 1 |  |  |
| LD ICS/LABA | 0.53 (0.31–0.89)* | 0.89 (0.6–1.24) | 0.87 (0.62–1.11) | 1 (0.7–1.34) | - | 1.1 (0.76–1.65) | 1 |  |
| LAMA | 0.48 (0.26–0.83)* | 0.8 (0.54–1.1) | 0.78 (0.51–1.1) | 0.89 (0.58–1.3) | - | 0.99 (0.66–1.51) | 0.9 (0.56–1.4) | 1 |
| At least 1 exacerbation event in the past year (9 studies, 30,254 patients) | | | | | | | | |
| Rank | 1 | 3 | 2 | 4 |  | 7 | 5 | 6 |
| SUCRA, % | 98.4 | 66.24 | 72.69 | 39.45 |  | 16.5 | 37.76 | 18.96 |
| NMA estimate OR (95% CrI) | | | | | | | | |
| Triple therapy with HD ICS | 1 |  |  |  |  |  |  |  |
| Triple therapy with MD ICS | 0.6 (0.36–0.999)* | 1 |  |  |  |  |  |  |
| Triple therapy with LD ICS | 0.61 (0.38–1.04) | 1.03 (0.78–1.4) | 1 |  |  |  |  |  |
| LABA/LAMA | 0.54 (0.35–0.83)* | 0.9 (0.68–1.18) | 0.88 (0.66–1.12) | 1 |  |  |  |  |
| HD ICS/LABA | - | - | - | - | - |  |  |  |
| MD ICS/LABA | 0.48 (0.27–0.8)* | 0.81 (0.6–0.996)* | 0.79 (0.53–1.03) | 0.9 (0.63–1.19) | - | 1 |  |  |
| LD ICS/LABA | 0.53 (0.3–0.9)* | 0.89 (0.59–1.27) | 0.87 (0.62–1.11) | 0.99 (0.69–1.35) | - | 1.1 (0.74–1.7) | 1 |  |
| LAMA | 0.48 (0.26–0.84)* | 0.8 (0.54–1.1) | 0.78 (0.5–1.1) | 0.89 (0.57–1.31) | - | 0.99 (0.65–1.53) | 0.9 (0.56–1.44) | 1 |
| mMRC ≥2 or CAT ≥10 (9 studies, 29,301 patients) | | | | | | | | |
| Rank | - | 2 | 1 | 4 | - | 5 | 3 | 6 |
| SUCRA, % | - | 83.23 | 90.89 | 40.9 | - | 21.73 | 45.06 | 18.19 |
| NMA estimate OR (95% CrI) | | | | | | | | |
| Triple therapy with HD ICS | - |  |  |  |  |  |  |  |
| Triple therapy with MD ICS | - | 1 |  |  |  |  |  |  |
| Triple therapy with LD ICS | - | 1.02 (0.89–1.21) | 1 |  |  |  |  |  |
| LABA/LAMA | - | 0.9 (0.78–1.02) | 0.88 (0.76–0.99)* | 1 |  |  |  |  |
| HD ICS/LABA | - | - | - | - | - |  |  |  |
| MD ICS/LABA | - | 0.85 (0.74–0.97)* | 0.84 (0.69–0.97)* | 0.95 (0.82–1.1) | - | 1 |  |  |
| LD ICS/LABA | - | 0.91 (0.73–1.09) | 0.89 (0.73–1.01) | 1.01 (0.84–1.19) | - | 1.06 (0.86–1.31) | 1 |  |
| LAMA | - | 0.83 (0.68–1.003) | 0.81 (0.63–1.01) | 0.93 (0.73–1.15) | - | 0.97 (0.77–1.23) | 0.92 (0.71–1.2) | 1 |
| Mean blood eosinophil count ≥200/uL (3 studies, 5,589 patients) | | | | | | | | |
| Rank | - | 1 | - | 2 | - | 4 | - | 3 |
| SUCRA, % | - | 82.6 | - | 55.38 | - | 29.05 | - | 32.96 |
| NMA estimate OR (95% CrI) | | | | | | | | |
| Triple therapy with HD ICS | - |  |  |  |  |  |  |  |
| Triple therapy with MD ICS | - | 1 |  |  |  |  |  |  |
| Triple therapy with LD ICS | - | - | - |  |  |  |  |  |
| LABA/LAMA | - | 0.93 (0.69–1.25) | - | 1 |  |  |  |  |
| HD ICS/LABA | - | - | - | - | - |  |  |  |
| MD ICS/LABA | - | 1.11 (0.73–1.69) | - | 1.19 (0.88–1.62) | - | 1 |  |  |
| LD ICS/LABA | - | - | - | - | - | - | - |  |
| LAMA | - | 1.08 (0.72–1.62) | - | 1.17 (0.89–1.52) | - | 0.98 (0.65–1.47) | - | 1 |
| Study duration ≥24 weeks (11 studies, 32,745 patients) | | | | | | | | |
| Rank | 1 | 2 | 3 | 5 | - | 7 | 6 | 4 |
| SUCRA, % | 96.55 | 72.26 | 65.01 | 33.8 | - | 13.12 | 29.56 | 39.7 |
| NMA estimate OR (95% CrI) | | | | | | | | |
| Triple therapy with HD ICS | 1 |  |  |  |  |  |  |  |
| Triple therapy with MD ICS | 0.74 (0.53–1.13) | 1 |  |  |  |  |  |  |
| Triple therapy with LD ICS | 0.72 (0.51–1.11) | 0.97 (0.73–1.27) | 1 |  |  |  |  |  |
| LABA/LAMA | 0.64 (0.48–0.89)* | 0.85 (0.68–1.05) | 0.88 (0.69–1.12) | 1 |  |  |  |  |
| HD ICS/LABA | - | - | - | - | - |  |  |  |
| MD ICS/LABA | 0.58 (0.4–0.86)* | 0.78 (0.61–0.93)* | 0.8 (0.59–1.05) | 0.91 (0.71–1.14) | - | 1 |  |  |
| LD ICS/LABA | 0.62 (0.41–0.98)* | 0.84 (0.57–1.16) | 0.86 (0.64–1.12) | 0.98 (0.7–1.31) | - | 1.07 (0.75–1.55) | 1 |  |
| LAMA | 0.65 (0.41–1.12) | 0.87 (0.63–1.21) | 0.9 (0.59–1.4) | 1.02 (0.69–1.54) | - | 1.12 (0.78–1.71) | 1.04 (0.67–1.76) | 1 |
| Study duration <48 weeks (6 studies, 6,607 patients) | | | | | | | | |
| Rank | - | 1 | - | 2 | - | 4 | - | 3 |
| SUCRA, % | - | 82.62 | - | 55.03 | - | 28.96 | - | 33.39 |
| NMA estimate OR (95% CrI) | | | | | | | | |
| Triple therapy with HD ICS | - |  |  |  |  |  |  |  |
| Triple therapy with MD ICS | - | 1 |  |  |  |  |  |  |
| Triple therapy with LD ICS | - | - | - |  |  |  |  |  |
| LABA/LAMA | - | 0.93 (0.69–1.25) | - | 1 |  |  |  |  |
| HD ICS/LABA | - | - | - | - | - |  |  |  |
| MD ICS/LABA | - | 0.84 (0.61–1.15) | - | 0.9 (0.59–1.39) | - | 1 |  |  |
| LD ICS/LABA | - | - | - | - | - | - | - |  |
| LAMA | - | 0.86 (0.65–1.13) | - | 0.92 (0.62–1.38) | - | 1.03 (0.68–1.56) | - | 1 |
| Study duration ≥48 weeks (3 studies, 5,589patients) | | | | | | | | |
| Rank | - | 1 | - | 2 | - | 4 | - | 3 |
| SUCRA, % | - | 83.07 | - | 55.25 | - | 29 | - | 32.69 |
| NMA estimate OR (95% CrI) | | | | | | | | |
| Triple therapy with HD ICS | - |  |  |  |  |  |  |  |
| Triple therapy with MD ICS | - | 1 |  |  |  |  |  |  |
| Triple therapy with LD ICS | - | - | - |  |  |  |  |  |
| LABA/LAMA | - | 0.93 (0.68–1.23) | - | 1 |  |  |  |  |
| HD ICS/LABA | - | - | - | - | - |  |  |  |
| MD ICS/LABA | - | 0.84 (0.61–1.14) | - | 0.9 (0.59–1.39) | - | 1 |  |  |
| LD ICS/LABA | - | - | - | - | - | - | - |  |
| LAMA | - | 0.85 (0.65–1.11) | - | 0.92 (0.63–1.38) | - | 1.02 (0.68–1.53) | - | 1 |

CrI= credible interval; HD= high-dose; ICS= inhaled corticosteroid; LABA= long-acting beta-agonist; LAMA= long-acting muscarinic antagonist; LD= low-dose; MD= medium-dose; NMA= network meta-analysis; OR= odds ratio; SUCRA= surface under the cumulative ranking curve

Median odds ratio with 95% credible interval was calculated as a row to column ratio. If the OR is significantly lower than 1, the drug in the left row is more beneficial than the other drug in the upper column.

Subgroup analysis in RCTs with study duration <48 weeks could not be evaluated because a complete network was not established.

* indicates that the posterior probability is either less than 0.025 or more than 0.975, which is considered statistically significant.

**Supplementary information 8. Sensitivity analyses to evaluate the risk of all-cause mortality associated with different inhaled therapies**

|  | Triple therapy with HD ICS | | Triple therapy with MD ICS | Triple therapy with LD ICS | LABA/LAMA | | HD ICS/LABA | MD ICS/LABA | LD ICS/LABA | LAMA |
| --- | --- | --- | --- | --- | --- | --- | --- | --- | --- | --- |
| FEV_1_ <65%, (16 studies, 34,645 patients) | | | | | | | | | | |
| Rank | 7 | | 1 | 3 | 8 | | 4 | 2 | 6 | 5 |
| SUCRA, % | 32.01 | | 85.87 | 56.14 | 20.52 | | 55.62 | 66.49 | 40.26 | 43.09 |
| NMA estimate OR (95% CrI) | | | | | | | | | | |
| Triple therapy with HD ICS | 1 | |  |  |  | |  |  |  |  |
| Triple therapy with MD ICS | 1.66 (0.89–3.06) | | 1 |  |  | |  |  |  |  |
| Triple therapy with LD ICS | 1.24 (0.67–2.25) | | 0.74 (0.49–1.14) | 1 |  | |  |  |  |  |
| LABA/LAMA | 0.94 (0.56–1.55) | | 0.57 (0.38–0.84)* | 0.76 (0.54–1.1) | 1 | |  |  |  |  |
| HD ICS/LABA | 3.56 (0–2.95×E+06) | | 2.17 (0–2.00×E+06) | 2.9 (0–2.69×E+06) | 3.87 (0–3.68×E+06) | | 1 |  |  |  |
| MD ICS/LABA | 1.38 (0.7–2.69) | | 0.83 (0.52–1.28) | 1.11 (0.68–1.81) | 1.46 (0.92–2.31) | | 0.39 (0–5.43×E+06) | 1 |  |  |
| LD ICS/LABA | 1.1 (0.56–2.05) | | 0.66 (0.38–1.1) | 0.89 (0.58–1.31) | 1.16 (0.74–1.75) | | 0.31 (0–5.33×E+06) | 0.8 (0.44–1.4) | 1 |  |
| LAMA | 1.11 (0.55–2.35) | | 0.67 (0.41–1.13) | 0.91 (0.5–1.7) | 1.18 (0.68–2.15) | | 0.31 (0–4.41×E+06) | 0.81 (0.43–1.61) | 1.03 (0.53–2.09) | 1 |
| At least 1 exacerbation event in the past year (13 studies, 33,095 patients) | | | | | | | | | | |
| Rank | 6 | | 1 | 3 | 7 | | - | 2 | 5 | 4 |
| SUCRA, % | 32.63 | | 92.9 | 55.78 | 16.6 | | - | 69.39 | 38.14 | 44.54 |
| NMA estimate OR (95% CrI) | | | | | | | | | | |
| Triple therapy with HD ICS | 1 | |  |  |  | |  |  |  |  |
| Triple therapy with MD ICS | 1.63 (0.89–3.17) | | 1 |  |  | |  |  |  |  |
| Triple therapy with LD ICS | 1.19 (0.65–2.22) | | 0.73 (0.46–1.11) | 1 |  | |  |  |  |  |
| LABA/LAMA | 0.92 (0.55–1.58) | | 0.56 (0.37–0.84)* | 0.77 (0.55–1.12) | 1 | |  |  |  |  |
| HD ICS/LABA | - | | - | - | - | | - |  |  |  |
| MD ICS/LABA | 1.35 (0.68–2.72) | | 0.82 (0.52–1.27) | 1.13 (0.69–1.89) | 1.45 (0.91–2.37) | | - | 1 |  |  |
| LD ICS/LABA | 1.06 (0.54–2.07) | | 0.65 (0.36–1.09) | 0.89 (0.58–1.34) | 1.15 (0.71–1.75) | | - | 0.79 (0.43–1.4) | 1 |  |
| LAMA | 1.11 (0.54–2.48) | | 0.68 (0.4–1.16) | 0.94 (0.49–1.81) | 1.21 (0.67–2.25) | | - | 0.82 (0.44–1.63) | 1.05 (0.51–2.25) | 1 |
| mMRC ≥2 or CAT ≥10 (15 studies, 34,032 patients) | | | | | | | | | | |
| Rank | - | | 1 | 3 | 6 | | - | 2 | 5 | 4 |
| SUCRA, % | - | | 81.83 | 61.2 | 21.29 | | - | 73.26 | 28.25 | 34.16 |
| NMA estimate OR (95% CrI) | | | | | | | | | | |
| Triple therapy with HD ICS | - | |  |  |  | |  |  |  |  |
| Triple therapy with MD ICS | - | | 1 |  |  | |  |  |  |  |
| Triple therapy with LD ICS | - | | 0.85 (0.53–1.47) | 1 |  | |  |  |  |  |
| LABA/LAMA | - | | 0.65 (0.43–1.09) | 0.77 (0.5–1.22) | 1 | |  |  |  |  |
| HD ICS/LABA | - | | - | - | - | | - |  |  |  |
| MD ICS/LABA | - | | 0.95 (0.6–1.62) | 1.11 (0.63–2) | 1.45 (0.84–2.49) | | - | 1 |  |  |
| LD ICS/LABA | - | | 0.68 (0.3–1.21) | 0.81 (0.38–1.21) | 1.05 (0.46–1.63) | | - | 0.72 (0.29–1.3) | 1 |  |
| LAMA | - | | 0.69 (0.35–1.58) | 0.81 (0.35–1.99) | 1.05 (0.46–2.59) | | - | 0.72 (0.31–1.81) | 1.01 (0.43–3.38) | 1 |
| Mean blood eosinophil count ≥200/uL (3 studies, 5,589 patients) | | | | | |  | | | | |
| Rank | - | | 1 | - | 3 | | - | 2 | - | 4 |
| SUCRA, % | - | | 73.52 | - | 41.15 | | - | 60.26 | - | 25.07 |
| NMA estimate OR (95% CrI) | | | | | | | | | | |
| Triple therapy with HD ICS | - | |  |  |  | |  |  |  |  |
| Triple therapy with MD ICS | - | | 1 |  |  | |  |  |  |  |
| Triple therapy with LD ICS | - | | - | - |  | |  |  |  |  |
| LABA/LAMA | - | | 0.75 (0.32–1.71) | - | 1 | |  |  |  |  |
| HD ICS/LABA | - | | - | - | - | | - |  |  |  |
| MD ICS/LABA | - | | 0.92 (0.38–2.27) | - | 1.23 (0.36–4.14) | | - | 1 |  |  |
| LD ICS/LABA | - | | - | - | - | | - | - | - |  |
| LAMA | - | | 0.63 (0.30–1.37) | - | 0.85 (0.27–2.60) | | - | 0.69 (0.22–2.22) | - | 1 |
| Study duration <24 weeks (11 studies, 6,816 patients) | | | | | | | | | | |
| Rank | 5 | | 3 | 2 | - | | 6 | 1 | 7 | 4 |
| SUCRA, % | 42.12 | | 59.8 | 63.16 | - | | 35.71 | 64.75 | 25.47 | 58.99 |
| NMA estimate OR (95% CrI) | | | | | | | | | | |
| Triple therapy with HD ICS | 1 | |  |  |  | |  |  |  |  |
| Triple therapy with MD ICS | 160.87 (0–4.63×E+16) | | 1 |  |  | |  |  |  |  |
| Triple therapy with LD ICS | 207.26 (0–4.15×E+16) | | 1.08 (0.05–45.78) | 1 |  | |  |  |  |  |
| LABA/LAMA | - | | - | - | - | |  |  |  |  |
| HD ICS/LABA | 0.48 (0.01–30.54) | | 0 (0–7.62×E+10) | 0 (0–5.93×E+10) | - | | 1 |  |  |  |
| MD ICS/LABA | 250.14 (0–1.42×E+17) | | 1.44 (0.06–72.16) | 1.35 (0.01–192.87) | - | | 569.99 (0–2.27×E+17) | 1 |  |  |
| LD ICS/LABA | 11.91 (0–2.43×E+15) | | 0.06 (0–7.33) | 0.06 (0–0.96)* | - | | 25.83 (0–4.29×E+15) | 0.04 (0–11.15) | 1 |  |
| LAMA | 161.23 (0–3.18×E+16) | | 0.94 (0.09–14.92) | 0.85 (0.04–16.89) | - | | 386.1 (0–7.63×E+16) | 0.67 (0.01–40.71) | 14.15 (0.22–1.52×E+03) | 1 |
| Study duration ≥24 weeks (13 studies, 34,188 patients) | | | | | | | | | | |
| Rank | 6 | 1 | | 3 | 7 | | - | 2 | 4 | 5 |
| SUCRA, % | 29.83 | 88 | | 58.77 | 18.93 | | - | 71.98 | 41.45 | 41.02 |
| NMA estimate OR (95% CrI) | | | | | | | | | | |
| Triple therapy with HD ICS | 1 |  | |  |  | |  |  |  |  |
| Triple therapy with MD ICS | 1.56 (0.85–2.84) | 1 | |  |  | |  |  |  |  |
| Triple therapy with LD ICS | 1.24 (0.69–2.23) | 0.8 (0.51–1.23) | | 1 |  | |  |  |  |  |
| LABA/LAMA | 0.96 (0.6–1.58) | 0.62 (0.43–0.9)* | | 0.78 (0.55–1.13) | 1 | |  |  |  |  |
| HD ICS/LABA | - | - | | - | - | | - |  |  |  |
| MD ICS/LABA | 1.39 (0.72–2.66) | 0.89 (0.58–1.38) | | 1.11 (0.69–1.84) | 1.44 (0.9–2.23) | | - | 1 |  |  |
| LD ICS/LABA | 1.11 (0.58–2.1) | 0.71 (0.42–1.21) | | 0.89 (0.59–1.34) | 1.16 (0.72–1.74) | | - | 0.8 (0.45–1.42) | 1 |  |
| LAMA | 1.1 (0.53–2.4) | 0.7 (0.41–1.29) | | 0.88 (0.45–1.8) | 1.14 (0.61–2.2) | | - | 0.79 (0.41–1.63) | 0.99 (0.48–2.14) | 1 |
| Study duration <48 weeks (17 studies, 13,617 patients) | | | | | | | | | | |
| Rank | 2 | 4 | | 6 | 1 | | 7 | 3 | 8 | 5 |
| SUCRA, % | 60.17 | 57.04 | | 47.53 | 70.35 | | 43.75 | 58.75 | 14.26 | 48.16 |
| NMA estimate OR (95% CrI) | | | | | | | | | | |
| Triple therapy with HD ICS | 1 |  | |  |  | |  |  |  |  |
| Triple therapy with MD ICS | 0.82 (0.03–26.19) | 1 | |  |  | |  |  |  |  |
| Triple therapy with LD ICS | 0.55 (0.01–38.26) | 0.71 (0.11–5.87) | | 1 |  | |  |  |  |  |
| LABA/LAMA | 1.32 (0.11–17.65) | 1.63 (0.15–16.2) | | 2.33 (0.09–45.47) | 1 | |  |  |  |  |
| HD ICS/LABA | 0.47 (0.01–27.82) | 0.58 (0–109.65) | | 0.81 (0–185.53) | 0.36 (0–41.98) | | 1 |  |  |  |
| MD ICS/LABA | 0.89 (0.03–27.73) | 1.09 (0.22–5.29) | | 1.52 (0.1–17.57) | 0.67 (0.06–7.37) | | 1.85 (0.01–471.81) | 1 |  |  |
| LD ICS/LABA | 0.15 (0–12.06) | 0.18 (0.01–2.53) | | 0.26 (0.03–1.3) | 0.11 (0–3.82) | | 0.31 (0–131.99) | 0.17 (0.01–3.54) | 1 |  |
| LAMA | 0.6 (0.01–46.1) | 0.75 (0.1–7.35) | | 1.06 (0.11–11.84) | 0.45 (0.02–14.03) | | 1.35 (0.01–506.27) | 0.69 (0.06–11.64) | 4.15 (0.27–103.21) | 1 |
| Study duration ≥48 weeks (7 studies, 27,387 patients) | | | | | | | | | | |
| Rank | 6 | 1 | | 3 | 7 | | - | 2 | 5 | 4 |
| SUCRA, % | 31.65 | 87.59 | | 56.24 | 16.52 | | - | 71.15 | 41.87 | 45.01 |
| NMA estimate OR (95% CrI) | | | | | | | | | | |
| Triple therapy with HD ICS | 1 |  | |  |  | |  |  |  |  |
| Triple therapy with MD ICS | 1.59 (0.81–3.15) | 1 | |  |  | |  |  |  |  |
| Triple therapy with LD ICS | 1.21 (0.64–2.35) | 0.77 (0.47–1.26) | | 1 |  | |  |  |  |  |
| LABA/LAMA | 0.93 (0.54–1.62) | 0.58 (0.38–0.9)* | | 0.76 (0.53–1.11) | 1 | |  |  |  |  |
| HD ICS/LABA | - | - | | - | - | | - |  |  |  |
| MD ICS/LABA | 1.39 (0.67–2.89) | 0.88 (0.54–1.43) | | 1.15 (0.67–1.93) | 1.5 (0.9–2.47) | | - | 1 |  |  |
| LD ICS/LABA | 1.1 (0.54–2.32) | 0.7 (0.38–1.29) | | 0.91 (0.57–1.48) | 1.19 (0.73–1.93) | | - | 0.79 (0.42–1.54) | 1 |  |
| LAMA | 1.14 (0.5–2.57) | 0.71 (0.4–1.31) | | 0.93 (0.45–1.93) | 1.22 (0.63–2.37) | | - | 0.81 (0.4–1.7) | 1.02 (0.47–2.29) | 1 |

CrI= credible interval; HD= high-dose; ICS= inhaled corticosteroid; LABA= long-acting beta-agonist; LAMA= long-acting muscarinic antagonist; LD= low-dose; MD= medium-dose; NMA= network meta-analysis; OR= odds ratio; SUCRA= surface under the cumulative ranking curve

Median odds ratio with 95% credible interval was calculated as a row to column ratio. If the OR is significantly lower than 1, the drug in the left row is more beneficial than the other drug in the upper column.

* indicates that the posterior probability is either less than 0.025 or more than 0.975, which is considered statistically significant.

**Supplementary information 9. Sensitivity analyses to evaluate the trough FEV_1_ change associated with different inhaled therapies**

|  | Triple therapy with HD ICS | Triple therapy with MD ICS | Triple therapy with LD ICS | LABA/LAMA | HD ICS/LABA | MD ICS/LABA | LD ICS/LABA | LAMA |
| --- | --- | --- | --- | --- | --- | --- | --- | --- |
| FEV_1_ <65%, (12 studies, 21,239 patients) | | | | | | | | |
| Rank | 3 | 2 | 1 | 4 | 6 | 7 | 8 | 5 |
| SUCRA, % | 70.35 | 78.49 | 95.5 | 46.57 | 35.08 | 32.39 | 5.97 | 35.65 |
| NMA estimate OR (95% CrI) | | | | | | | | |
| Triple therapy with HD ICS | 0 |  |  |  |  |  |  |  |
| Triple therapy with MD ICS | -12.2 (-100–77.2) | 0 |  |  |  |  |  |  |
| Triple therapy with LD ICS | -38 (-128.4–53.5) | -26 (-64.6–13) | 0 |  |  |  |  |  |
| LABA/LAMA | 32.7 (-77.4–146.2) | 44.5 (-30.7–122.2) | 70.8 (7.1–137.2)* | 0 |  |  |  |  |
| HD ICS/LABA | 46.3 (-28.4–124.5) | 58.8 (-30.7–144.9) | 84.8 (-7–174.9) | 13.7 (-101.5–125.5) | 0 |  |  |  |
| MD ICS/LABA | 50.8 (-49.8–155) | 62.5 (9.7–114.8)* | 88.6 (23.9–155.3)* | 18.3 (-75.9–108.5) | 3.4 (-98.2–107.3) | 0 |  |  |
| LD ICS/LABA | 93.2 (-9.4–199.8) | 105.1 (43.7–169.3)* | 131.2 (83–181.9)* | 60.4 (-6.6–125.9) | 46.4 (-53.8–153.3) | 42.6 (-37.3–126.1) | 0 |  |
| LAMA | 44.9 (-30.8–122.8) | 57.3 (11.3–100.1)* | 83.3 (31–132.9)* | 12.5 (-71.2–93.2) | -1.7 (-77.5–75.4) | -4.8 (-75.2–62.1) | -47.9 (-120.8–20) | 0 |
| At least 1 exacerbation event in the past year (8 studies, 19,263 patients) | | | | | | | | |
| Rank | - | 2 | 1 | 3 | - | 5 | 6 | 4 |
| SUCRA, % | - | 73.65 | 95.71 | 53.25 | - | 28.63 | 12.11 | 36.64 |
| NMA estimate OR (95% CrI) | | | | | | | | |
| Triple therapy with HD ICS | - |  |  |  |  |  |  |  |
| Triple therapy with MD ICS | - | 0 |  |  |  |  |  |  |
| Triple therapy with LD ICS | - | -43.7 (-127.9–41.9) | 0 |  |  |  |  |  |
| LABA/LAMA | - | 28.3 (-96.5–159.7) | 71.6 (-26.4–171.6) | 0 |  |  |  |  |
| HD ICS/LABA | - | - | - | - | - |  |  |  |
| MD ICS/LABA | - | 62.2 (-14.3–135.9) | 105.6 (-7.7–214.3) | 33.9 (-118.2–181.4) | - | 0 |  |  |
| LD ICS/LABA | - | 89.5 (-20.7–202.3) | 132.2 (60.7–210.8)* | 61 (-34.6–158.2) | - | 27.2 (-104.4–165.1) | 0 |  |
| LAMA | - | 50.9 (-21.4–121.6) | 94 (8.4–177.6)* | 22.5 (-112.5–147.2) | - | -11.5 (-114.4–91.1) | -38.7 (-154.4–71.3) | 0 |
| mMRC ≥2 or CAT ≥10 (12 studies, 22,974 patients) | | | | | | | | |
| Rank | - | 2 | 1 | 3 | - | 4 | 6 | 5 |
| SUCRA, % | - | 79.79 | 98.92 | 58.59 | - | 31.6 | 6.18 | 24.93 |
| NMA estimate OR (95% CrI) | | | | | | | | |
| Triple therapy with HD ICS | - |  |  |  |  |  |  |  |
| Triple therapy with MD ICS | - | 0 |  |  |  |  |  |  |
| Triple therapy with LD ICS | - | -25.6 (-55.8–5.5) | 0 |  |  |  |  |  |
| LABA/LAMA | - | 32.6 (-8.9–75.9) | 58.5 (16.3–99.3)* | 0 |  |  |  |  |
| HD ICS/LABA | - | - | - | - | - |  |  |  |
| MD ICS/LABA | - | 68.1 (25.6–112.2)* | 93.5 (42.9–143.1)* | 35.1 (-16.2–85.6) | - | 0 |  |  |
| LD ICS/LABA | - | 98.9 (58–141.3)* | 124.3 (93.9–155.3)* | 66 (20.9–112.4)* | - | 30.9 (-25.4–88.6) | 0 |  |
| LAMA | - | 76.6 (38.1–116.8)* | 102.2 (58.7–145.1)* | 43.7 (-10.2–99.5) | - | 8.5 (-48.6–66.8) | -22.3 (-74.7–29.3) | 0 |
| Study duration <24 weeks (7 studies, 4,082 patients) | | | | | | | | |
| Rank | 3 | 2 | 1 | - | 5 | - | 6 | 4 |
| SUCRA, % | 65.76 | 76.2 | 93.51 | - | 29.3 | - | 4.73 | 30.49 |
| NMA estimate OR (95% CrI) | | | | | | | | |
| Triple therapy with HD ICS | 1 |  |  |  |  |  |  |  |
| Triple therapy with MD ICS | -20.4 (-98.2–69.3) | 1 |  |  |  |  |  |  |
| Triple therapy with LD ICS | -35.5 (-110.8–49.3) | -15.1 (-55.2–20.6) | 1 |  |  |  |  |  |
| LABA/LAMA | - | - | - | - |  |  |  |  |
| HD ICS/LABA | 46.2 (-14.2–106.9) | 66.4 (-24.3–144.5) | 81.6 (-0.6–157.8) | - | 1 |  |  |  |
| MD ICS/LABA | - | - | - | - | - | - |  |  |
| LD ICS/LABA | 84.8 (1.2–178.6)* | 105.5 (45.6–159.6)* | 120.5 (78.3–161.4)* | - | 38.8 (-45.3–132.3) | - | 1 |  |
| LAMA | 45.3 (-15–106) | 65.2 (5.7–117.1)* | 80.8 (26.1–127.4)* | - | -1.1 (-62.9–61.1) | - | -39.7 (-107.3–22.1) | 1 |
| Study duration ≥24 weeks (10 studies, 20,741 patients) | | | | | | | | |
| Rank | - | 2 | 1 | 3 | - | 4 | 6 | 5 |
| SUCRA, % | - | 76.53 | 98.53 | 60.6 | - | 30.89 | 14.51 | 18.95 |
| NMA estimate OR (95% CrI) | | | | | | | | |
| Triple therapy with HD ICS | - |  |  |  |  |  |  |  |
| Triple therapy with MD ICS | - | 1 |  |  |  |  |  |  |
| Triple therapy with LD ICS | - | -51.1 (-116.3–13) | 1 |  |  |  |  |  |
| LABA/LAMA | - | 21.4 (-38.6–83.7) | 73.1 (12.6–136.4)* | 1 |  |  |  |  |
| HD ICS/LABA | - | - | - | - | - |  |  |  |
| MD ICS/LABA | - | 64.2 (17.7–111.4)* | 115.4 (41.2–189.2)* | 42.7 (-26.5–106.6) | - | 1 |  |  |
| LD ICS/LABA | - | 86.3 (15.6–169)* | 137.5 (90.5–198.6)* | 64.7 (1.1–139)* | - | 22.5 (-56.6–113.1) | 1 |  |
| LAMA | - | 80.1 (25.2–133)* | 131.6 (46.4–214.8)* | 58.6 (-26.3–138.8) | - | 15.9 (-56.7–88.6) | -6.9 (-110.8–81.9) | 1 |
| Study duration <48 weeks (14 studies, 10,412 patients) | | | | | | | | |
| Rank | 4 | 2 | 1 | 3 | 6 | 5 | 8 | 7 |
| SUCRA, % | 61.62 | 81.06 | 96.28 | 66.1 | 27.87 | 35.84 | 3.5 | 27.73 |
| NMA estimate OR (95% CrI) | | | | | | | | |
| Triple therapy with HD ICS | 1 |  |  |  |  |  |  |  |
| Triple therapy with MD ICS | -31.1 (-105.8–47.9) | 1 |  |  |  |  |  |  |
| Triple therapy with LD ICS | -52.9 (-132.1–28.3) | -22 (-55.3–11.6) | 1 |  |  |  |  |  |
| LABA/LAMA | -9.9 (-100.5–87.3) | 21.3 (-33.6–77.3) | 43 (-20.5–109.5) | 1 |  |  |  |  |
| HD ICS/LABA | 45.8 (-20.1–111.4) | 76.7 (-2–153.1) | 99 (18.2–176.3)* | 55.8 (-40.4–149.6) | 1 |  |  |  |
| MD ICS/LABA | 33.7 (-53.3–124.9) | 65 (20.8–108.8)* | 86.6 (32.9–141.7)* | 43.5 (-13.2–100.4) | -12.2 (-98.3–78.1) | 1 |  |  |
| LD ICS/LABA | 90 (7.1–178.7)* | 121 (73.1–170.6)* | 142.7 (108.4–180.3)* | 99.9 (25.6–173)* | 43.7 (-38.6–134.5) | 55.9 (-7.6–122.1) | 1 |  |
| LAMA | 44.8 (-19.9–110.6) | 75.8 (33.5–115.9)* | 97.5 (51.3–140.8)* | 54.5 (-16.3–121.6) | -0.9 (-66.6–65.4) | 10.8 (-50.7–70.5) | -45.2 (-105.4–10) | 1 |
| Study duration ≥48 weeks (3 studies, 14,411 patients) | | | | | | | | |
| Rank | - | 1 | 2 | 3 | - | 4 | 6 | 5 |
| SUCRA, % | - | 67.97 | 66.71 | 47.92 | - | 42.84 | 31.88 | 42.68 |
| NMA estimate OR (95% CrI) | | | | | | | | |
| Triple therapy with HD ICS | - |  |  |  |  |  |  |  |
| Triple therapy with MD ICS | - | 1 |  |  |  |  |  |  |
| Triple therapy with LD ICS | - | 32.5 (-1632.3–1676.4) | 1 |  |  |  |  |  |
| LABA/LAMA | - | 86.5 (-1577.4–1714.4) | 53.8 (-67.7–177.3) | 1 |  |  |  |  |
| HD ICS/LABA | - | - | - | - | - |  |  |  |
| MD ICS/LABA | - | 63.2 (-63.2–189.5) | 32.8 (-1609.4–1693.7) | -22 (-1662.2–1636.6) | - | 1 |  |  |
| LD ICS/LABA | - | 126.8 (-1532–1780.1) | 96.9 (-24.9–220) | 43.3 (-79.5–167.6) | - | 62.6 (-1587.8–1711.4) | 1 |  |
| LAMA | - | 63.5 (-59.8–183.9) | 32.1 (-1614.3–1684.1) | -19.1 (-1656.6–1633.8) | - | 0.1 (-177.6–174.2) | -62.6 (-1709.4–1593.6) | 1 |

CrI= credible interval; FEV_1_= forced expiratory volume in one second; HD= high-dose; ICS= inhaled corticosteroid; LABA= long-acting beta-agonist; LAMA= long-acting muscarinic antagonist; LD= low-dose; MD= medium-dose; NMA= network meta-analysis; SUCRA= surface under the cumulative ranking curve

Mean difference with 95% credible interval was calculated by subtracting row (left) from column (upper). If the mean difference is significantly lower than 0, the drug in the left row is more beneficial than the other drug in the upper column.

Subgroup analysis in RCTs with mean blood eosinophil count ≥200/uL could not be evaluated because a complete network was not established.

* indicates that the posterior probability is either less than 0.025 or more than 0.975, which is considered statistically significant

**Supplementary information 10. Sensitivity analyses to evaluate the SGRQ score change associated with different inhaled therapies**

|  | Triple therapy with HD ICS | Triple therapy with MD ICS | Triple therapy with LD ICS | LABA/LAMA | HD ICS/LABA | MD ICS/LABA | LD ICS/LABA | LAMA |
| --- | --- | --- | --- | --- | --- | --- | --- | --- |
| FEV_1_ <65%, (11 studies, 24,347 patients) | | | | | | | | |
| Rank | 3 | 1 | 2 | 5 | - | 4 | 6 | 7 |
| SUCRA, % | 69.47 | 88.7 | 86.79 | 32.35 | - | 40.22 | 28.92 | 3.54 |
| NMA estimate OR (95% CrI) | | | | | | | | |
| Triple therapy with HD ICS | 1 |  |  |  |  |  |  |  |
| Triple therapy with MD ICS | 0.7 (-1.2–2.6) | 1 |  |  |  |  |  |  |
| Triple therapy with LD ICS | 0.6 (-1.2–2.5) | -0.1 (-0.8–0.7) | 1 |  |  |  |  |  |
| LABA/LAMA | -1.2 (-2.8–0.4) | -1.9 (-2.9–-0.9)* | -1.8 (-2.8–-0.9)* | 1 |  |  |  |  |
| HD ICS/LABA | - | - | - | - | - |  |  |  |
| MD ICS/LABA | -1 (-3.2–0.9) | -1.7 (-3–-0.6)* | -1.6 (-2.9–-0.5)* | 0.2 (-1.2–1.4) | - | 1 |  |  |
| LD ICS/LABA | -1.3 (-3.2–0.6) | -2 (-3.2–-0.8)* | -1.9 (-2.9–-1)* | -0.1 (-1.2–1) | - | -0.3 (-1.7–1.3) | 1 |  |
| LAMA | -2.5 (-4.7–-0.2)* | -3.1 (-4.6–-1.8)* | -3.1 (-4.6–-1.7)* | -1.2 (-2.9–0.4) | - | -1.4 (-3.2–0.4) | -1.1 (-2.8–0.5) | 1 |
| At least 1 exacerbation event in the past year (9 studies, 22,910 patients) | | | | | | | | |
| Rank | 3 | 1 | 2 | 5 | - | 4 | 6 | 7 |
| SUCRA, % | 68.62 | 87.34 | 86.06 | 33.44 | - | 38.79 | 30.29 | 5.46 |
| NMA estimate OR (95% CrI) | | | | | | | | |
| Triple therapy with HD ICS | 1 |  |  |  |  |  |  |  |
| Triple therapy with MD ICS | 0.7 (-1.9–3.3) | 1 |  |  |  |  |  |  |
| Triple therapy with LD ICS | 0.6 (-1.8–3.2) | -0.1 (-1.3–1.3) | 1 |  |  |  |  |  |
| LABA/LAMA | -1.2 (-3.3–0.8) | -1.9 (-3.5–-0.4)* | -1.8 (-3.2–-0.6)* | 1 |  |  |  |  |
| HD ICS/LABA | - | - | - | - | - |  |  |  |
| MD ICS/LABA | -1.1 (-3.9–1.5) | -1.7 (-3.5–-0.4)* | -1.7 (-3.6–-0.3)* | 0.2 (-1.8–1.7) | - | 1 |  |  |
| LD ICS/LABA | -1.3 (-4–1.3) | -2 (-3.8–-0.3)* | -1.9 (-3.4–-0.6)* | -0.1 (-1.7–1.5) | - | -0.3 (-2.1–2) | 1 |  |
| LAMA | -2.5 (-5.5–0.4) | -3.2 (-4.9–-1.5)* | -3.1 (-4.9–-1.4)* | -1.3 (-3.4–0.7) | - | -1.4 (-3.4–0.9) | -1.2 (-3.3–1) | 1 |
| mMRC ≥2 or CAT ≥10 (12 studies, 24,139 patients) | | | | | | | | |
| Rank | - | 2 | 1 | 4 | - | 3 | 5 | 6 |
| SUCRA, % | - | 88.65 | 91.22 | 32.86 | - | 50.88 | 32.72 | 3.67 |
| NMA estimate OR (95% CrI) | | | | | | | | |
| Triple therapy with HD ICS | - |  |  |  |  |  |  |  |
| Triple therapy with MD ICS | - | 1 |  |  |  |  |  |  |
| Triple therapy with LD ICS | - | 0.1 (-0.6–0.7) | 1 |  |  |  |  |  |
| LABA/LAMA | - | -1.6 (-2.4–-0.9)* | -1.7 (-2.4–-1)* | 1 |  |  |  |  |
| HD ICS/LABA | - | - | - | - | - |  |  |  |
| MD ICS/LABA | - | -1.3 (-2–-0.6)* | -1.3 (-2.2–-0.5)* | 0.4 (-0.5–1.2) | - | 1 |  |  |
| LD ICS/LABA | - | -1.7 (-2.5–-0.7)* | -1.7 (-2.3–-1)* | 0 (-0.9–0.8) | - | -0.4 (-1.4–0.6) | 1 |  |
| LAMA | - | -3.1 (-5.1–-1.1)* | -3.2 (-5.1–-1.3)* | -1.5 (-3.5–0.5) | - | -1.8 (-3.9–0.2) | -1.5 (-3.5–0.5) | 1 |
| Study duration ≥24 weeks (9 studies, 23,976 patients) | | | | | | | | |
| Rank | 3 | 1 | 2 | 6 | - | 5 | 4 | - |
| SUCRA, % | 64.66 | 73.83 | 73.48 | 19.9 | - | 22.97 | 45.19 | - |
| NMA estimate OR (95% CrI) | | | | | | | | |
| Triple therapy with HD ICS | 1 |  |  |  |  |  |  |  |
| Triple therapy with MD ICS | 0.2 (-3.3–3.6) | 1 |  |  |  |  |  |  |
| Triple therapy with LD ICS | 0.2 (-3.3–3.6) | 0 (-2.3–2.2) | 1 |  |  |  |  |  |
| LABA/LAMA | -1.4 (-4–1.2) | -1.6 (-3.8–0.7) | -1.6 (-3.8–0.7) | 1 |  |  |  |  |
| HD ICS/LABA | - | - | - | - | - |  |  |  |
| MD ICS/LABA | -1.3 (-5–2.1) | -1.5 (-3.8–0.5) | -1.5 (-4.2–0.9) | 0 (-2.4–2.3) | - | 1 |  |  |
| LD ICS/LABA | -0.7 (-4.1–3.4) | -0.9 (-3.5–2.5) | -0.9 (-2.7–1.7) | 0.7 (-1.7–3.9) | - | 0.7 (-2.1–4.4) | 1 |  |
| LAMA | - | - | - | - | - | - | - | - |
| Study duration <48 weeks (16 studies, 12,951 patients) | | | | | | | | |
| Rank | 3 | 2 | 1 | 5 | 7 | 6 | 4 | 8 |
| SUCRA, % | 68.22 | 74.31 | 79.17 | 38.57 | 24.55 | 35.08 | 65.52 | 14.56 |
| NMA estimate OR (95% CrI) | | | | | | | | |
| Triple therapy with HD ICS | 1 |  |  |  |  |  |  |  |
| Triple therapy with MD ICS | 0.2 (-4.9–5.3) | 1 |  |  |  |  |  |  |
| Triple therapy with LD ICS | 0.4 (-4.9–5.8) | 0.2 (-1.8–2.3) | 1 |  |  |  |  |  |
| LABA/LAMA | -1.5 (-5.3–2.2) | -1.7 (-5–1.6) | -1.9 (-5.8–1.9) | 1 |  |  |  |  |
| HD ICS/LABA | -2.7 (-6.5–1.1) | -3 (-9.3–3.4) | -3.2 (-9.9–3.6) | -1.3 (-6.6–4.2) | 1 |  |  |  |
| MD ICS/LABA | -1.6 (-6.7–3.5) | -1.8 (-3.8–0.1) | -2 (-4.9–0.8) | -0.1 (-3.4–3.3) | 1.2 (-5.2–7.4) | 1 |  |  |
| LD ICS/LABA | -0.1 (-5.7–6) | -0.3 (-2.9–2.8) | -0.5 (-2.3–1.7) | 1.4 (-2.8–6) | 2.6 (-4.2–9.8) | 1.5 (-1.7–5.2) | 1 |  |
| LAMA | -3 (-8.7–2.7) | -3.2 (-5.8–-0.7)* | -3.4 (-6.3–-0.8)* | -1.5 (-5.7–2.7) | -0.3 (-7.1–6.6) | -1.4 (-4.6–1.7) | -2.9 (-6.6–0.2) | 1 |
| Study duration ≥48 weeks (3 studies, 17,453 patients) | | | | | | | | |
| Rank | 3 | 1 | 2 | 5 | - | 4 | 6 | - |
| SUCRA, % | 61.26 | 85.47 | 77.27 | 19.27 | - | 37.75 | 18.98 | - |
| NMA estimate OR (95% CrI) | | | | | | | | |
| Triple therapy with HD ICS | 1 |  |  |  |  |  |  |  |
| Triple therapy with MD ICS | 0.8 (-2.3–3.8) | 1 |  |  |  |  |  |  |
| Triple therapy with LD ICS | 0.5 (-2.3–3.2) | -0.3 (-2.4–1.7) | 1 |  |  |  |  |  |
| LABA/LAMA | -1.2 (-3.5–1.1) | -2 (-3.9–0.1) | -1.7 (-3.2–-0.1)* | 1 |  |  |  |  |
| HD ICS/LABA | - | - | - | - | - |  |  |  |
| MD ICS/LABA | -0.7 (-3.8–2.4) | -1.5 (-3.7–0.6) | -1.2 (-3.2–0.8) | 0.5 (-1.6–2.5) | - | 1 |  |  |
| LD ICS/LABA | -1.3 (-4.3–1.7) | -2.1 (-4.6–0.5) | -1.7 (-3.7–0.2) | -0.1 (-2.1–1.9) | - | -0.6 (-3.2–2) | 1 |  |
| LAMA | - | - | - | - | - | - | - | - |

CrI= credible interval; HD= high-dose; ICS= inhaled corticosteroid; LABA= long-acting beta-agonist; LAMA= long-acting muscarinic antagonist; LD= low-dose; MD= medium-dose; NMA= network meta-analysis; SGRQ= St. George's respiratory questionnaire; SUCRA= surface under the cumulative ranking curve

Mean difference with 95% credible interval was calculated by subtracting row (left) from column (upper). If the mean difference is significantly lower than 0, the drug in the left row is more beneficial than the other drug in the upper column.

Subgroup analyses in RCTs with mean blood eosinophil count ≥200/uL and study duration <24 weeks could not be evaluated because a complete network was not established.

* indicates that the posterior probability is either less than 0.025 or more than 0.975, which is considered statistically significant

**Supplementary information 11. Sensitivity analyses to evaluate the risk of serious adverse events associated with different inhaled therapies**

|  | Triple therapy with HD ICS | Triple therapy with MD ICS | Triple therapy with LD ICS | LABA/LAMA | HD ICS/LABA | MD ICS/LABA | LD ICS/LABA | LAMA |
| --- | --- | --- | --- | --- | --- | --- | --- | --- |
| FEV_1_ <65%, (17 studies, 35,102 patients) | | | | | | | | |
| Rank | 4 | 1 | 6 | 5 | 3 | 8 | 2 | 7 |
| SUCRA, % | 52.1 | 80.88 | 40.2 | 40.89 | 55.14 | 33.92 | 61.62 | 35.25 |
| NMA estimate OR (95% CrI) | | | | | | | | |
| Triple therapy with HD ICS | 1 |  |  |  |  |  |  |  |
| Triple therapy with MD ICS | 1.1 (0.8–1.56) | 1 |  |  |  |  |  |  |
| Triple therapy with LD ICS | 0.97 (0.68–1.32) | 0.88 (0.71–1.04) | 1 |  |  |  |  |  |
| LABA/LAMA | 0.97 (0.73–1.28) | 0.88 (0.72–1.05) | 1 (0.85–1.22) | 1 |  |  |  |  |
| HD ICS/LABA | 1.9 (0–7.35×E+05) | 1.73 (0–6.55×E+05) | 1.96 (0–7.55×E+05) | 1.96 (0–7.48×E+05) | 1 |  |  |  |
| MD ICS/LABA | 0.94 (0.65–1.33) | 0.86 (0.68–1.02) | 0.98 (0.78–1.21) | 0.97 (0.77–1.2) | 0.5 (0–9.36×E+07) | 1 |  |  |
| LD ICS/LABA | 1.03 (0.7–1.45) | 0.94 (0.69–1.17) | 1.07 (0.86–1.3) | 1.07 (0.82–1.31) | 0.54 (0–1.01×E+08) | 1.09 (0.82–1.45) | 1 |  |
| LAMA | 0.93 (0.64–1.4) | 0.85 (0.66–1.09) | 0.97 (0.73–1.32) | 0.96 (0.73–1.31) | 0.5 (0–9.45×E+07) | 0.99 (0.73–1.38) | 0.91 (0.66–1.32) | 1 |
| At least 1 exacerbation event in the past year (13 studies, 33,097 patients) | | | | | | | | |
| Rank | 3 | 5 | 1 | 2 | - | 6 | 4 | 7 |
| SUCRA, % | 54.49 | 42.76 | 86.86 | 67.95 | - | 34.57 | 42.84 | 20.5 |
| NMA estimate OR (95% CrI) | | | | | | | | |
| Triple therapy with HD ICS | 1 |  |  |  |  |  |  |  |
| Triple therapy with MD ICS | 1.1 (0.82–1.55) | 1 |  |  |  |  |  |  |
| Triple therapy with LD ICS | 0.97 (0.7–1.31) | 0.88 (0.71–1.04) | 1 |  |  |  |  |  |
| LABA/LAMA | 0.97 (0.75–1.28) | 0.89 (0.73–1.05) | 1 (0.86–1.21) | 1 |  |  |  |  |
| HD ICS/LABA | - | - | - | - | - |  |  |  |
| MD ICS/LABA | 0.95 (0.67–1.32) | 0.86 (0.69–1.02) | 0.97 (0.79–1.21) | 0.97 (0.78–1.19) | - | 1 |  |  |
| LD ICS/LABA | 1.04 (0.72–1.43) | 0.95 (0.7–1.18) | 1.07 (0.87–1.3) | 1.07 (0.84–1.3) | - | 1.1 (0.83–1.43) | 1 |  |
| LAMA | 0.87 (0.6–1.3) | 0.79 (0.61–1.03) | 0.9 (0.67–1.24) | 0.9 (0.67–1.22) | - | 0.92 (0.68–1.28) | 0.84 (0.61–1.23) | 1 |
| mMRC ≥2 or CAT ≥10 (16 studies, 34,375 patients) | | | | | | | | |
| Rank | - | 1 | 4 | 5 | - | 3 | 2 | 6 |
| SUCRA, % | - | 91.92 | 43.49 | 34.1 | - | 48.29 | 51.52 | 30.7 |
| NMA estimate OR (95% CrI) | | | | | | | | |
| Triple therapy with HD ICS | - |  |  |  |  |  |  |  |
| Triple therapy with MD ICS | - | 1 |  |  |  |  |  |  |
| Triple therapy with LD ICS | - | 0.87 (0.68–1.03) | 1 |  |  |  |  |  |
| LABA/LAMA | - | 0.85 (0.67–1.01) | 0.98 (0.8–1.19) | 1 |  |  |  |  |
| HD ICS/LABA | - | - | - | - | - |  |  |  |
| MD ICS/LABA | - | 0.88 (0.69–1.05) | 1.01 (0.8–1.3) | 1.03 (0.82–1.3) | - | 1 |  |  |
| LD ICS/LABA | - | 0.89 (0.59–1.11) | 1.02 (0.76–1.23) | 1.05 (0.75–1.3) | - | 1.01 (0.69–1.31) | 1 |  |
| LAMA | - | 0.81 (0.58–1.13) | 0.94 (0.66–1.39) | 0.96 (0.67–1.44) | - | 0.92 (0.64–1.4) | 0.92 (0.63–1.52) | 1 |
| Mean blood eosinophil count ≥200/uL (3 studies, 5,589 patients) | | | | | | | |  |
| Rank | - | 1 | - | 2 | - | 3 | - | 4 |
| SUCRA, % | - | 74.85 | - | 66.35 | - | 30.55 | - | 28.25 |
| NMA estimate OR (95% CrI) | | | | | | | | |
| Triple therapy with HD ICS | - |  |  |  |  |  |  |  |
| Triple therapy with MD ICS | - | 1 |  |  |  |  |  |  |
| Triple therapy with LD ICS | - | - | - |  |  |  |  |  |
| LABA/LAMA | - | 0.99 (0.67–1.46) | - | 1 |  |  |  |  |
| HD ICS/LABA | - | - | - | - | - |  |  |  |
| MD ICS/LABA | - | 0.83 (0.57–1.20) | - | 0.84 (0.49–1.44) | - | 1 |  |  |
| LD ICS/LABA | - | - | - | - | - | - | - |  |
| LAMA | - | 0.82 (0.52–1.17) | - | 0.83 (0.49–1.41) | - | 0.99 (0.59–1.65) | - | 1 |
| Study duration <24 weeks (11 studies, 6,818 patients) | | | | | | | | |
| Rank | - | 1 | 4 | 5 | - | 3 | 2 | 6 |
| SUCRA, % | - | 91.95 | 44.05 | 34.56 | - | 48.05 | 52.13 | 29.27 |
| NMA estimate OR (95% CrI) | | | | | | | | |
| Triple therapy with HD ICS | - |  |  |  |  |  |  |  |
| Triple therapy with MD ICS | - | 1 |  |  |  |  |  |  |
| Triple therapy with LD ICS | - | 0.87 (0.68–1.03) | 1 |  |  |  |  |  |
| LABA/LAMA | - | 0.85 (0.67–1.01) | 0.98 (0.8–1.2) | 1 |  |  |  |  |
| HD ICS/LABA | - | - | - | - | - |  |  |  |
| MD ICS/LABA | - | 0.88 (0.69–1.05) | 1.01 (0.8–1.28) | 1.03 (0.82–1.3) | - | 1 |  |  |
| LD ICS/LABA | - | 0.89 (0.59–1.11) | 1.02 (0.76–1.23) | 1.05 (0.75–1.28) | - | 1.01 (0.69–1.31) | 1 |  |
| LAMA | - | 0.81 (0.57–1.12) | 0.93 (0.65–1.39) | 0.95 (0.66–1.43) | - | 0.92 (0.63–1.39) | 0.91 (0.62–1.52) | 1 |
| Study duration ≥24 weeks (14 studies, 34,805 patients) | | | | | | | | |
| Rank | 4 | 1 | 5 | 6 | - | 3 | 2 | 7 |
| SUCRA, % | 43.44 | 86.93 | 42.38 | 33.11 | - | 45.69 | 65.86 | 32.57 |
| NMA estimate OR (95% CrI) | | | | | | | | |
| Triple therapy with HD ICS | 1 |  |  |  |  |  |  |  |
| Triple therapy with MD ICS | 1.13 (0.87–1.52) | 1 |  |  |  |  |  |  |
| Triple therapy with LD ICS | 1 (0.77–1.33) | 0.89 (0.72–1.05) | 1 |  |  |  |  |  |
| LABA/LAMA | 0.98 (0.79–1.25) | 0.87 (0.73–1.01) | 0.98 (0.84–1.16) | 1 |  |  |  |  |
| HD ICS/LABA | - | - | - | - | - |  |  |  |
| MD ICS/LABA | 1.01 (0.77–1.37) | 0.9 (0.75–1.05) | 1.01 (0.83–1.24) | 1.03 (0.86–1.24) | - | 1 |  |  |
| LD ICS/LABA | 1.06 (0.78–1.44) | 0.95 (0.71–1.16) | 1.06 (0.86–1.27) | 1.08 (0.85–1.31) | - | 1.05 (0.79–1.32) | 1 |  |
| LAMA | 0.96 (0.67–1.41) | 0.85 (0.65–1.11) | 0.95 (0.7–1.34) | 0.97 (0.72–1.33) | - | 0.94 (0.7–1.3) | 0.9 (0.64–1.32) | 1 |
| Study duration <48 weeks (18 studies, 14,236 patients) | | | | | | | | |
| Rank | 7 | 1 | 4 | 6 | 3 | 5 | 8 | 2 |
| SUCRA, % | 32.61 | 87.61 | 53.01 | 35.74 | 53.65 | 48.48 | 29.72 | 59.16 |
| NMA estimate OR (95% CrI) | | | | | | | | |
| Triple therapy with HD ICS | - |  |  |  |  |  |  |  |
| Triple therapy with MD ICS | 1.75 (0.58–5.51) | - |  |  |  |  |  |  |
| Triple therapy with LD ICS | 1.28 (0.38–4.44) | 0.73 (0.45–1.2) | - |  |  |  |  |  |
| LABA/LAMA | 1.07 (0.46–2.54) | 0.62 (0.29–1.25) | 0.84 (0.34–1.97) | - |  |  |  |  |
| HD ICS/LABA | 1.27 (0.5–3.24) | 0.73 (0.16–3.11) | 1.01 (0.2–4.61) | 1.19 (0.33–4.19) | - |  |  |  |
| MD ICS/LABA | 1.24 (0.39–3.64) | 0.7 (0.42–1.09) | 0.97 (0.47–1.81) | 1.15 (0.54–2.3) | 0.97 (0.22–4.08) | - |  |  |
| LD ICS/LABA | 1.01 (0.26–3.79) | 0.58 (0.27–1.2) | 0.79 (0.43–1.33) | 0.94 (0.33–2.64) | 0.79 (0.15–4.18) | 0.82 (0.34–2) | - |  |
| LAMA | 1.38 (0.41–4.69) | 0.78 (0.49–1.26) | 1.07 (0.57–1.95) | 1.28 (0.53–3.03) | 1.06 (0.23–5.18) | 1.11 (0.59–2.28) | 1.35 (0.6–3.11) | - |
| Study duration ≥48 weeks (7 studies, 27,387 patients) | | | | | | | | |
| Rank | 3 | 2 | 4 | 5 | - | 6 | 1 | 7 |
| SUCRA, % | 54.14 | 70.17 | 48.71 | 39.87 | - | 38.24 | 78.19 | 20.69 |
| NMA estimate OR (95% CrI) | | | | | | | | |
| Triple therapy with HD ICS | 1 |  |  |  |  |  |  |  |
| Triple therapy with MD ICS | 1.04 (0.78–1.41) | 1 |  |  |  |  |  |  |
| Triple therapy with LD ICS | 0.99 (0.74–1.32) | 0.95 (0.78–1.14) | 1 |  |  |  |  |  |
| LABA/LAMA | 0.97 (0.76–1.24) | 0.93 (0.78–1.11) | 0.98 (0.84–1.15) | 1 |  |  |  |  |
| HD ICS/LABA | - | - | - | - | - |  |  |  |
| MD ICS/LABA | 0.96 (0.7–1.31) | 0.92 (0.76–1.1) | 0.97 (0.79–1.18) | 0.99 (0.81–1.2) | - | 1 |  |  |
| LD ICS/LABA | 1.07 (0.78–1.46) | 1.03 (0.8–1.31) | 1.08 (0.89–1.33) | 1.1 (0.9–1.35) | - | 1.11 (0.87–1.46) | 1 |  |
| LAMA | 0.87 (0.59–1.3) | 0.84 (0.62–1.13) | 0.88 (0.63–1.24) | 0.9 (0.65–1.24) | - | 0.91 (0.65–1.29) | 0.81 (0.56–1.18) | 1 |

CrI= credible interval; HD= high-dose; ICS= inhaled corticosteroid; LABA= long-acting beta-agonist; LAMA= long-acting muscarinic antagonist; LD= low-dose; MD= medium-dose; NMA= network meta-analysis; OR= odds ratio; SUCRA= surface under the cumulative ranking curve

Median odds ratio with 95% credible interval was calculated as a row to column ratio. If the OR is significantly lower than 1, the drug in the left row is more beneficial than the other drug in the upper column.

* indicates that the posterior probability is either less than 0.025 or more than 0.975, which is considered statistically significant.

**Supplementary information 12. Sensitivity analyses to evaluate the risk of serious cardiac adverse events associated with different inhaled therapies**

|  | Triple therapy with HD ICS | Triple therapy with MD ICS | Triple therapy with LD ICS | LABA/LAMA | HD ICS/LABA | MD ICS/LABA | LD ICS/LABA | LAMA |
| --- | --- | --- | --- | --- | --- | --- | --- | --- |
| FEV_1_ <65%, (14 studies, 33,851 patients) | | | | | | | | |
| Rank | 5 | 2 | 3 | 7 | - | 1 | 6 | 4 |
| SUCRA, % | 35.9 | 68.18 | 64.32 | 21.92 | - | 88.29 | 25.28 | 46.11 |
| NMA estimate OR (95% CrI) | | | | | | | | |
| Triple therapy with HD ICS | 1 |  |  |  |  |  |  |  |
| Triple therapy with MD ICS | 1.31 (0.65–2.68) | 1 |  |  |  |  |  |  |
| Triple therapy with LD ICS | 1.27 (0.61–2.64) | 0.97 (0.65–1.44) | 1 |  |  |  |  |  |
| LABA/LAMA | 0.93 (0.49–1.73) | 0.71 (0.48–0.997)* | 0.73 (0.48–1.08) | 1 |  |  |  |  |
| HD ICS/LABA | - | - | - | - | - |  |  |  |
| MD ICS/LABA | 1.63 (0.73–3.57) | 1.24 (0.78–1.98) | 1.28 (0.76–2.16) | 1.75 (1.06–2.96)* | - | 1 |  |  |
| LD ICS/LABA | 0.92 (0.39–2.15) | 0.7 (0.36–1.27) | 0.72 (0.42–1.21) | 0.99 (0.54–1.75) | - | 0.56 (0.27–1.13) | 1 |  |
| LAMA | 1.11 (0.49–2.71) | 0.85 (0.48–1.55) | 0.88 (0.46–1.78) | 1.2 (0.65–2.43) | - | 0.68 (0.34–1.46) | 1.22 (0.55–2.94) | 1 |
| At least 1 exacerbation event in the past year (12 studies, 32,391 patients) | | | | | | | | |
| Rank | 5 | 3 | 2 | 7 | - | 1 | 6 | 4 |
| SUCRA, % | 35.88 | 61.72 | 72.2 | 21.7 | - | 85.52 | 29.89 | 43.06 |
| NMA estimate OR (95% CrI) | | | | | | | | |
| Triple therapy with HD ICS | 1 |  |  |  |  |  |  |  |
| Triple therapy with MD ICS | 1.25 (0.59–2.66) | 1 |  |  |  |  |  |  |
| Triple therapy with LD ICS | 1.36 (0.61–2.98) | 1.09 (0.66–1.78) | 1 |  |  |  |  |  |
| LABA/LAMA | 0.92 (0.48–1.76) | 0.74 (0.49–1.09) | 0.68 (0.43–1.08) | 1 |  |  |  |  |
| HD ICS/LABA | - | - | - | - | - |  |  |  |
| MD ICS/LABA | 1.57 (0.69–3.66) | 1.27 (0.77–2.08) | 1.17 (0.64–2.1) | 1.71 (1.01–2.95)* | - | 1 |  |  |
| LD ICS/LABA | 0.96 (0.38–2.31) | 0.77 (0.38–1.5) | 0.71 (0.4–1.22) | 1.04 (0.54–1.9) | - | 0.61 (0.28–1.3) | 1 |  |
| LAMA | 1.06 (0.44–2.75) | 0.86 (0.48–1.57) | 0.79 (0.38–1.7) | 1.16 (0.6–2.34) | - | 0.67 (0.32–1.5) | 1.11 (0.47–2.76) | 1 |
| mMRC ≥2 or CAT ≥10 (16 studies, 34,375 patients) | | | | | | | | |
| Rank | - | 2 | 3 | 5 | - | 1 | 6 | 4 |
| SUCRA, % | - | 75.03 | 67.4 | 21.42 | - | 75.94 | 16.63 | 43.57 |
| NMA estimate OR (95% CrI) | | | | | | | | |
| Triple therapy with HD ICS | - |  |  |  |  |  |  |  |
| Triple therapy with MD ICS | - | 1 |  |  |  |  |  |  |
| Triple therapy with LD ICS | - | 0.95 (0.63–1.39) | 1 |  |  |  |  |  |
| LABA/LAMA | - | 0.69 (0.47–0.97)* | 0.72 (0.48–1.08) | 1 |  |  |  |  |
| HD ICS/LABA | - | - | - | - | - |  |  |  |
| MD ICS/LABA | - | 1.02 (0.64–1.54) | 1.08 (0.64–1.75) | 1.49 (0.91–2.4) | - | 1 |  |  |
| LD ICS/LABA | - | 0.63 (0.35–1.1) | 0.66 (0.42–1.05) | 0.92 (0.53–1.58) | - | 0.62 (0.33–1.18) | 1 |  |
| LAMA | - | 0.8 (0.42–1.53) | 0.84 (0.39–1.79) | 1.17 (0.56–2.47) | - | 0.78 (0.37–1.74) | 1.26 (0.56–3.02) | 1 |
| Mean blood eosinophil count ≥200/uL (3 studies, 5,589 patients) | | | | | | | |  |
| Rank | - | 1 | - | 3 | - | 2 | - | 4 |
| SUCRA, % | - | 66.39 | - | 39.97 | - | 60.69 | - | 32.95 |
| NMA estimate OR (95% CrI) | | | | | | | | |
| Triple therapy with HD ICS | - |  |  |  |  |  |  |  |
| Triple therapy with MD ICS | - | 1 |  |  |  |  |  |  |
| Triple therapy with LD ICS | - | - | - |  |  |  |  |  |
| LABA/LAMA | - | 0.86 (0.51–1.42) | - | 1 |  |  |  |  |
| HD ICS/LABA | - | - | - | - | - |  |  |  |
| MD ICS/LABA | - | 1.00 (0.46–2.22) | - | 1.18 (0.46–2.92) | - | 1 |  |  |
| LD ICS/LABA | - | - | - | - | - | - | - |  |
| LAMA | - | 0.79 (0.42–1.51) | - | 0.93 (0.42–2.14) | - | 0.79 (0.29–2.17) | - | 1 |
| Study duration ≥24 weeks (13 studies, 33,824 patients) | | | | | | | | |
| Rank | 4 | 3 | 2 | 7 | - | 1 | 5 | 6 |
| SUCRA, % | 49.49 | 61.42 | 75.2 | 22.02 | - | 80.27 | 31.32 | 30.27 |
| NMA estimate OR (95% CrI) | | | | | | | | |
| Triple therapy with HD ICS | 1 |  |  |  |  |  |  |  |
| Triple therapy with MD ICS | 1.1 (0.56–2.26) | 1 |  |  |  |  |  |  |
| Triple therapy with LD ICS | 1.23 (0.59–2.59) | 1.11 (0.68–1.84) | 1 |  |  |  |  |  |
| LABA/LAMA | 0.82 (0.46–1.48) | 0.75 (0.51–1.08) | 0.67 (0.42–1.06) | 1 |  |  |  |  |
| HD ICS/LABA | - | - | - | - | - |  |  |  |
| MD ICS/LABA | 1.3 (0.6–2.81) | 1.18 (0.72–1.88) | 1.06 (0.57–1.85) | 1.58 (0.93–2.56) | - | 1 |  |  |
| LD ICS/LABA | 0.86 (0.37–1.99) | 0.78 (0.39–1.53) | 0.7 (0.4–1.22) | 1.04 (0.56–1.93) | - | 0.66 (0.32–1.42) | 1 |  |
| LAMA | 0.83 (0.34–2.16) | 0.76 (0.4–1.42) | 0.68 (0.31–1.51) | 1.01 (0.5–2.08) | - | 0.65 (0.3–1.42) | 0.98 (0.39–2.44) | 1 |
| Study duration <48 weeks (16 studies, 13,165 patients) | | | | | | | | |
| Rank | 2 | 3 | 5 | 6 | 1 | 7 | 8 | 4 |
| SUCRA, % | 70.8 | 63.24 | 48.04 | 44.52 | 74.61 | 28.85 | 16.43 | 53.51 |
| NMA estimate OR (95% CrI) | | | | | | | | |
| Triple therapy with HD ICS | 1 |  |  |  |  |  |  |  |
| Triple therapy with MD ICS | 0.68 (0.08–5.54) | 1 |  |  |  |  |  |  |
| Triple therapy with LD ICS | 0.51 (0.05–4.69) | 0.78 (0.36–1.7) | 1 |  |  |  |  |  |
| LABA/LAMA | 0.53 (0.13–1.89) | 0.79 (0.15–3.96) | 1.01 (0.17–5.9) | 1 |  |  |  |  |
| HD ICS/LABA | 1.21 (0.3–5.18) | 1.86 (0.14–23.27) | 2.43 (0.15–33.43) | 2.31 (0.35–18.05) | 1 |  |  |  |
| MD ICS/LABA | 0.37 (0.04–3.02) | 0.56 (0.21–1.4) | 0.72 (0.21–2.32) | 0.71 (0.14–3.53) | 0.31 (0.02–3.88) | 1 |  |  |
| LD ICS/LABA | 0.28 (0.03–2.94) | 0.42 (0.15–1.18) | 0.54 (0.26–1.12) | 0.54 (0.08–3.72) | 0.22 (0.02–3.85) | 0.75 (0.19–3.17) | 1 |  |
| LAMA | 0.61 (0.04–8.44) | 0.91 (0.19–4.24) | 1.17 (0.23–5.53) | 1.16 (0.13–10.41) | 0.49 (0.02–10.24) | 1.63 (0.26–10.59) | 2.19 (0.37–12.54) | 1 |
| Study duration ≥48 weeks (7 studies, 27,387 patients) | | | | | | | | |
| Rank | 5 | 3 | 2 | 7 | - | 1 | 4 | 6 |
| SUCRA, % | 36.62 | 60.94 | 70.95 | 22.41 | - | 84.97 | 40.07 | 34.05 |
| NMA estimate OR (95% CrI) | | | | | | | | |
| Triple therapy with HD ICS | 1 |  |  |  |  |  |  |  |
| Triple therapy with MD ICS | 1.25 (0.57–2.73) | 1 |  |  |  |  |  |  |
| Triple therapy with LD ICS | 1.36 (0.59–3.09) | 1.09 (0.62–1.88) | 1 |  |  |  |  |  |
| LABA/LAMA | 0.92 (0.47–1.79) | 0.74 (0.48–1.12) | 0.68 (0.41–1.12) | 1 |  |  |  |  |
| HD ICS/LABA | - | - | - | - | - |  |  |  |
| MD ICS/LABA | 1.59 (0.67–3.74) | 1.27 (0.76–2.14) | 1.17 (0.63–2.2) | 1.73 (0.99–3.01) | - | 1 |  |  |
| LD ICS/LABA | 1.05 (0.39–2.73) | 0.83 (0.38–1.78) | 0.77 (0.39–1.53) | 1.14 (0.56–2.24) | - | 0.66 (0.28–1.5) | 1 |  |
| LAMA | 0.98 (0.38–2.67) | 0.78 (0.41–1.56) | 0.72 (0.31–1.71) | 1.06 (0.51–2.34) | - | 0.61 (0.27–1.47) | 0.94 (0.35–2.67) | 1 |

CrI= credible interval; HD= high-dose; ICS= inhaled corticosteroid; LABA= long-acting beta-agonist; LAMA= long-acting muscarinic antagonist; LD= low-dose; MD= medium-dose; NMA= network meta-analysis; OR= odds ratio; SUCRA= surface under the cumulative ranking curve

Median odds ratio with 95% credible interval was calculated as a row to column ratio. If the OR is significantly lower than 1, the drug in the left row is more beneficial than the other drug in the upper column.

Subgroup analysis in RCTs with study duration <24 weeks could not be evaluated because a complete network was not established.

* indicates that the posterior probability is either less than 0.025 or more than 0.975, which is considered statistically significant.

**Supplementary information 13. Sensitivity analyses to evaluate the risk of pneumonia associated with different inhaled therapies**

|  | Triple therapy with HD ICS | Triple therapy with MD ICS | Triple therapy with LD ICS | LABA/LAMA | HD ICS/LABA | MD ICS/LABA | LD ICS/LABA | LAMA |
| --- | --- | --- | --- | --- | --- | --- | --- | --- |
| FEV_1_ <65%, (16 studies, 35,012 patients) | | | | | | | | |
| Rank | 2 | 5 | 6 | 1 | - | 7 | 4 | 3 |
| SUCRA, % | 68.6 | 33.31 | 27.77 | 84.43 | - | 16.15 | 57.14 | 62.61 |
| NMA estimate OR (95% CrI) | | | | | | | | |
| Triple therapy with HD ICS | 1 |  |  |  |  |  |  |  |
| Triple therapy with MD ICS | 0.73 (0.35–1.74) | 1 |  |  |  |  |  |  |
| Triple therapy with LD ICS | 0.71 (0.34–1.63) | 0.97 (0.63–1.44) | 1 |  |  |  |  |  |
| LABA/LAMA | 1.1 (0.58–2.22) | 1.5 (0.94–2.22) | 1.55 (1.01–2.3)* | 1 |  |  |  |  |
| HD ICS/LABA | - | - | - | - | - |  |  |  |
| MD ICS/LABA | 0.64 (0.29–1.57) | 0.87 (0.56–1.32) | 0.9 (0.55–1.51) | 0.58 (0.36–1.01) | - | 1 |  |  |
| LD ICS/LABA | 0.86 (0.42–2.42) | 1.18 (0.69–2.34) | 1.2 (0.82–2.25) | 0.78 (0.51–1.57) | - | 1.34 (0.74–2.92) | 1 |  |
| LAMA | 0.93 (0.37–2.5) | 1.26 (0.7–2.27) | 1.3 (0.67–2.58) | 0.84 (0.43–1.68) | - | 1.44 (0.71–2.92) | 1.07 (0.43–2.24) | 1 |
| At least 1 exacerbation event in the past year (13 studies, 33,097 patients) | | | | | | | | |
| Rank | 2 | 5 | 6 | 1 | - | 7 | 4 | 3 |
| SUCRA, % | 66.67 | 37.26 | 24.91 | 81.59 | - | 18.48 | 55.67 | 65.41 |
| NMA estimate OR (95% CrI) | | | | | | | | |
| Triple therapy with HD ICS | 1 |  |  |  |  |  |  |  |
| Triple therapy with MD ICS | 0.75 (0.34–1.98) | 1 |  |  |  |  |  |  |
| Triple therapy with LD ICS | 0.7 (0.31–1.69) | 0.93 (0.54–1.44) | 1 |  |  |  |  |  |
| LABA/LAMA | 1.09 (0.54–2.37) | 1.45 (0.84–2.22) | 1.56 (0.97–2.51) | 1 |  |  |  |  |
| HD ICS/LABA | - | - | - | - | - |  |  |  |
| MD ICS/LABA | 0.65 (0.28–1.78) | 0.86 (0.52–1.37) | 0.93 (0.54–1.73) | 0.59 (0.35–1.1) | - | 1 |  |  |
| LD ICS/LABA | 0.86 (0.38–2.62) | 1.14 (0.61–2.39) | 1.22 (0.78–2.43) | 0.78 (0.47–1.66) | - | 1.33 (0.66–2.99) | 1 |  |
| LAMA | 0.97 (0.36–2.8) | 1.28 (0.66–2.41) | 1.39 (0.65–2.96) | 0.89 (0.42–1.91) | - | 1.49 (0.66–3.19) | 1.12 (0.41–2.56) | 1 |
| mMRC ≥2 or CAT ≥10 (16 studies, 34,375 patients) | | | | | | | | |
| Rank | - | 4 | 5 | 1 | - | 6 | 3 | 2 |
| SUCRA, % | - | 29.77 | 28.08 | 84.65 | - | 23.48 | 53.34 | 80.67 |
| NMA estimate OR (95% CrI) | | | | | | | | |
| Triple therapy with HD ICS | - |  |  |  |  |  |  |  |
| Triple therapy with MD ICS | - | 1 |  |  |  |  |  |  |
| Triple therapy with LD ICS | - | 1 (0.67–1.46) | 1 |  |  |  |  |  |
| LABA/LAMA | - | 1.5 (0.995–2.14) | 1.51 (1.003–2.17)* | 1 |  |  |  |  |
| HD ICS/LABA | - | - | - | - | - |  |  |  |
| MD ICS/LABA | - | 0.96 (0.67–1.46) | 0.96 (0.63–1.59) | 0.64 (0.43–1.07) | - | 1 |  |  |
| LD ICS/LABA | - | 1.15 (0.71–2.07) | 1.14 (0.8–1.88) | 0.76 (0.51–1.35) | - | 1.2 (0.68–2.17) | 1 |  |
| LAMA | - | 1.57 (0.79–3.2) | 1.57 (0.75–3.45) | 1.05 (0.5–2.35) | - | 1.63 (0.73–3.54) | 1.36 (0.57–3.11) | 1 |
| Mean blood eosinophil count ≥200/uL (3 studies, 5,589 patients) | | | | | | | |  |
| Rank | - | 4 | - | 3 | - | 2 | - | 1 |
| SUCRA, % | - | 27.58 | - | 36.62 | - | 59.63 | - | 76.17 |
| NMA estimate OR (95% CrI) | | | | | | | | |
| Triple therapy with HD ICS | - |  |  |  |  |  |  |  |
| Triple therapy with MD ICS | - | 1 |  |  |  |  |  |  |
| Triple therapy with LD ICS | - | - | - |  |  |  |  |  |
| LABA/LAMA | - | 1.29 (0.6–2.82) | - | 1 |  |  |  |  |
| HD ICS/LABA | - | - | - | - | - |  |  |  |
| MD ICS/LABA | - | 1.04 (0.51–2.07) | - | 0.81 (0.29–2.27) | - | 1 |  |  |
| LD ICS/LABA | - | - | - | - | - | - | - |  |
| LAMA | - | 1.51 (0.75–3.13) | - | 1.18 (0.41–3.42) | - | 1.46 (0.54–4) | - | 1 |
| Study duration ≥24 weeks (15 studies, 34,985 patients) | | | | | | | | |
| Rank | 3 | 5 | 6 | 2 | - | 7 | 4 | 1 |
| SUCRA, % | 58.63 | 34.62 | 21.05 | 78.52 | - | 20.61 | 50.65 | 85.94 |
| NMA estimate OR (95% CrI) | | | | | | | | |
| Triple therapy with HD ICS | 1 |  |  |  |  |  |  |  |
| Triple therapy with MD ICS | 0.8 (0.43–1.83) | 1 |  |  |  |  |  |  |
| Triple therapy with LD ICS | 0.74 (0.38–1.58) | 0.92 (0.54–1.38) | 1 |  |  |  |  |  |
| LABA/LAMA | 1.14 (0.68–2.12) | 1.42 (0.89–2.04) | 1.54 (1.03–2.36)* | 1 |  |  |  |  |
| HD ICS/LABA | - | - | - | - | - |  |  |  |
| MD ICS/LABA | 0.73 (0.39–1.73) | 0.91 (0.61–1.36) | 0.98 (0.64–1.76) | 0.64 (0.42–1.1) | - | 1 |  |  |
| LD ICS/LABA | 0.9 (0.46–2.35) | 1.12 (0.63–2.16) | 1.2 (0.82–2.22) | 0.78 (0.5–1.52) | - | 1.23 (0.67–2.41) | 1 |  |
| LAMA | 1.38 (0.58–4.38) | 1.71 (0.88–3.73) | 1.86 (0.88–5.02) | 1.22 (0.58–3.09) | - | 1.89 (0.88–4.53) | 1.52 (0.62–3.99) | 1 |
| Study duration <48 weeks (18 studies, 14,326 patients) | | | | | | | | |
| Rank | 7 | 4 | 5 | 2 | 8 | 6 | 1 | 3 |
| SUCRA, % | 35.54 | 53.27 | 50.81 | 55.91 | 22.7 | 43.08 | 82.88 | 55.82 |
| NMA estimate OR (95% CrI) | | | | | | | | |
| Triple therapy with HD ICS | 1 |  |  |  |  |  |  |  |
| Triple therapy with MD ICS | 1.49 (0.23–9.54) | 1 |  |  |  |  |  |  |
| Triple therapy with LD ICS | 1.47 (0.19–11.51) | 0.99 (0.43–2.36) | 1 |  |  |  |  |  |
| LABA/LAMA | 1.52 (0.38–6.51) | 1.02 (0.31–3.63) | 1.03 (0.23–4.76) | 1 |  |  |  |  |
| HD ICS/LABA | 0.51 (0.04–5.77) | 0.35 (0.02–7.33) | 0.34 (0.01–8.13) | 0.33 (0.02–5.67) | 1 |  |  |  |
| MD ICS/LABA | 1.3 (0.21–8.62) | 0.87 (0.43–1.92) | 0.88 (0.29–2.75) | 0.85 (0.25–2.88) | 2.52 (0.13–55.14) | 1 |  |  |
| LD ICS/LABA | 2.83 (0.27–25.24) | 1.87 (0.48–6.55) | 1.89 (0.65–4.82) | 1.85 (0.27–9.9) | 5.45 (0.19–147.85) | 2.17 (0.43–8.66) | 1 |  |
| LAMA | 1.57 (0.19–12.87) | 1.06 (0.4–2.87) | 1.07 (0.34–3.44) | 1.02 (0.21–4.8) | 3.13 (0.12–75.57) | 1.2 (0.36–4.09) | 0.56 (0.13–2.74) | 1 |
| Study duration ≥48 weeks (7 studies, 27,387 patients) | | | | | | | | |
| Rank | 3 | 6 | 5 | 2 | - | 7 | 4 | 1 |
| SUCRA, % | 63.61 | 32.63 | 33.57 | 77.38 | - | 24.31 | 38.33 | 80.17 |
| NMA estimate OR (95% CrI) | | | | | | | | |
| Triple therapy with HD ICS | 1 |  |  |  |  |  |  |  |
| Triple therapy with MD ICS | 0.74 (0.31–2.37) | 1 |  |  |  |  |  |  |
| Triple therapy with LD ICS | 0.74 (0.29–2.25) | 1.01 (0.47–1.9) | 1 |  |  |  |  |  |
| LABA/LAMA | 1.1 (0.5–2.67) | 1.49 (0.74–2.45) | 1.48 (0.78–2.58) | 1 |  |  |  |  |
| HD ICS/LABA | - | - | - | - | - |  |  |  |
| MD ICS/LABA | 0.68 (0.27–2.39) | 0.93 (0.49–1.76) | 0.92 (0.46–2.09) | 0.62 (0.34–1.43) | - | 1 |  |  |
| LD ICS/LABA | 0.76 (0.25–2.68) | 1.04 (0.37–2.45) | 1.04 (0.45–2.25) | 0.7 (0.32–1.64) | - | 1.13 (0.37–2.79) | 1 |  |
| LAMA | 1.26 (0.41–6.7) | 1.69 (0.73–4.91) | 1.69 (0.62–6.58) | 1.15 (0.46–4.37) | - | 1.83 (0.66–6.21) | 1.64 (0.53–7.82) | 1 |

CrI= credible interval; HD= high-dose; ICS= inhaled corticosteroid; LABA= long-acting beta-agonist; LAMA= long-acting muscarinic antagonist; LD= low-dose; MD= medium-dose; NMA= network meta-analysis; OR= odds ratio; SUCRA= surface under the cumulative ranking curve

Median odds ratio with 95% credible interval was calculated as a row to column ratio. If the OR is significantly lower than 1, the drug in the left row is more beneficial than the other drug in the upper column.

Subgroup analysis in RCTs with study duration <24 weeks could not be evaluated because a complete network was not established.

* indicates that the posterior probability is either less than 0.025 or more than 0.975, which is considered statistically significant.

**Supplementary information 14. Consistency analysis between direct and indirect effect size**

| Treatment | Comparator | Indirect OR estimated from NMA | | Direct OR based estimated from paired meta-analysis | | Consistency assumption |
| --- | --- | --- | --- | --- | --- | --- |
|  |  | **Posterior median** | **95% CrIs** | **OR** | **95% CIs** | **P-value** |
| Total exacerbation | | | | | | |
| Triple therapy with LD ICS | LABA/LAMA | 0.72 | 0.45–1.14 | 0.90 | 0.67–1.22 | 0.395 |
| Triple therapy with MD ICS | LABA/LAMA | 0.73 | 0.49–1.09 | 0.93 | 0.70–1.23 | 0.313 |
| Triple therapy with HD ICS | LABA/LAMA | 0.59 | 0.12–3.00 | 0.68 | 0.53–0.93 | 0.838 |
| LD ICS/LABA | LABA/LAMA | 1.30 | 1.22–2.05 | 0.96 | 0.63–1.45 | 0.285 |
| MD ICS/LABA | LABA/LAMA | 1.35 | 1.11–2.05 | 1.10 | 0.82–1.55 | 0.405 |
| LAMA | LABA/LAMA | 1.15 | 1.19–1.58 | 0.91 | 0.51–1.73 | 0.489 |
| Triple therapy with MD ICS | Triple therapy with LD ICS | 1.05 | 0.78–1.46 | 1.10 | 0.76–1.67 | 0.847 |
| MD ICS/LABA | Triple therapy with LD ICS | 1.14 | 1.32–1.70 | 1.68 | 1.21–2.53 | 0.120 |
| LAMA | Triple therapy with LD ICS | 1.67 | 1.10–3.00 | 1.21 | 0.88–1.70 | 0.357 |
| LAMA | Triple therapy with MD ICS | 1.25 | 0.95–1.65 | 1.22 | 0.78–1.95 | 0.919 |
| LAMA | Triple therapy with HD ICS | 1.25 | 0.70–2.27 | 1.72 | 1.07–2.66 | 0.391 |
| LAMA | HD ICS/LABA | 1.01 | 0.22–4.95 | 1.23 | 0.59–2.44 | 0.818 |
| Moderate-to-severe exacerbation | | | | | | |
| Triple therapy with LD ICS | LABA/LAMA | 0.59 | 0.25–1.38 | 0.90 | 0.65–1.25 | 0.131 |
| Triple therapy with MD ICS | LABA/LAMA | 0.80 | 0.41–1.72 | 0.89 | 0.63–1.21 | 0.757 |
| LD ICS/LABA | LABA/LAMA | 1.21 | 0.61–2.32 | 0.96 | 0.59–1.55 | 0.491 |
| MD ICS/LABA | LABA/LAMA | 1.34 | 0.80–2.39 | 1.07 | 0.76–1.51 | 0.394 |
| Triple therapy with MD ICS | Triple therapy with LD ICS | 1.07 | 0.72–1.70 | 1.01 | 0.66–1.60 | 0.851 |
| MD ICS/LABA | Triple therapy with LD ICS | 1.55 | 1.01–2.56 | 1.14 | 0.74–1.73 | 0.215 |
| LAMA | Triple therapy with LD ICS | 1.11 | 0.69–1.68 | 1.65 | 0.92–3.00 | 0.268 |
| LAMA | Triple therapy with MD ICS | 1.72 | 0.88–3.32 | 1.15 | 0.80–1.62 | 0.273 |
| All-cause mortality | | | | | | |
| Triple therapy with LD ICS | LABA/LAMA | 1.36 | 0.33–5.47 | 0.76 | 0.50–1.16 | 0.448 |
| Triple therapy with MD ICS | LABA/LAMA | 0.43 | 0.17–1.04 | 0.71 | 0.42–1.46 | 0.323 |
| Triple therapy with HD ICS | LABA/LAMA | 1.09 | 0–3.58xE+09 | 0.94 | 0.58–1.54 | 0.984 |
| LD ICS/LABA | LABA/LAMA | 3.00 | 0.84–13.46 | 0.83 | 0.44–1.51 | 0.077 |
| MD ICS/LABA | LABA/LAMA | 0.67 | 0.25–1.80 | 0.71 | 0.38–1.38 | 0.891 |
| LAMA | LABA/LAMA | 1.01 | 0.50–2.08 | 0.67 | 6.69–2.61 | 0.572 |
| Triple therapy with MD ICS | Triple therapy with LD ICS | 1.01 | 0.41–2.25 | 0.72 | 0.37–1.49 | 0.526 |
| MD ICS/LABA | Triple therapy with LD ICS | 0.98 | 0.40–2.51 | 0.85 | 0.40–1.82 | 0.798 |
| LAMA | Triple therapy with LD ICS | 1.19 | 1.63–2.44 | 0.51 | 0.04–4.95 | 0.509 |
| LAMA | Triple therapy with MD ICS | 0.84 | 0.25–2.69 | 1.68 | 0.91–3.32 | 0.288 |
| LAMA | Triple therapy with HD ICS | 1.07 | 0.41–2.72 | 0.59 | 0.14–2.66 | 0.519 |
| LAMA | HD ICS/LABA | 0.50 | 0.02–16.44 | 0.41 | 0–2.65xE+10 | 0.998 |
| Serious adverse event | | | | | | |
| Triple therapy with LD ICS | LABA/LAMA | 1.15 | 0.73–1.77 | 0.98 | 0.79–1.23 | 0.552 |
| Triple therapy with MD ICS | LABA/LAMA | 0.66 | 0.48–0.91 | 0.95 | 0.77–1.13 | 0.067 |
| Triple therapy with HD ICS | LABA/LAMA | 13.46 | 0.01–4.42.E+05 | 0.98 | 0.77–1.28 | 0.519 |
| LD ICS/LABA | LABA/LAMA | 1.26 | 0.80–2.05 | 0.89 | 0.64–1.25 | 0.178 |
| MD ICS/LABA | LABA/LAMA | 1.22 | 0.84–1.84 | 0.93 | 0.66–1.17 | 0.189 |
| LAMA | LABA/LAMA | 1.03 | 0.75–1.40 | 1.06 | 0.41–2.69 | 0.950 |
| Triple therapy with MD ICS | Triple therapy with LD ICS | 0.85 | 0.58–1.20 | 0.87 | 0.67–1.08 | 0.943 |
| MD ICS/LABA | Triple therapy with LD ICS | 1.06 | 0.74–1.49 | 0.98 | 0.66–1.43 | 0.703 |
| LAMA | Triple therapy with LD ICS | 1.09 | 0.81–1.49 | 0.76 | 0.30–1.84 | 0.464 |
| LAMA | Triple therapy with MD ICS | 1.05 | 0.53–2.10 | 1.26 | 0.96–1.65 | 0.590 |
| LAMA | Triple therapy with HD ICS | 1.05 | 0.66–1.58 | 1.06 | 0.43–2.61 | 0.995 |
| LAMA | HD ICS/LABA | 1.35 | 0.62–2.72 | 0.05 | 0–1.99.E+04 | 0.484 |
| Serious cardiac adverse event | | | | | | |
| Triple therapy with LD ICS | LABA/LAMA | 1.08 | 0.49–2.41 | 0.66 | 0.40–1.09 | 0.346 |
| Triple therapy with MD ICS | LABA/LAMA | 0.43 | 0.20–1.01 | 0.76 | 0.52–1.13 | 0.212 |
| LD ICS/LABA | LABA/LAMA | 1.52 | 1.57–3.67 | 0.76 | 0.41–1.82 | 0.320 |
| MD ICS/LABA | LABA/LAMA | 0.93 | 0.42–2.05 | 0.58 | 0.33–1.11 | 0.334 |
| LAMA | LABA/LAMA | 0.85 | 0.40–1.65 | 0.88 | 0.10–6.69 | 0.974 |
| Triple therapy with MD ICS | Triple therapy with LD ICS | 1.03 | 0.44–2.36 | 0.93 | 0.57–1.49 | 0.860 |
| MD ICS/LABA | Triple therapy with LD ICS | 1.35 | 0.64–3.00 | 0.76 | 0.38–1.57 | 0.279 |
| LAMA | Triple therapy with LD ICS | 1.28 | 0.63–2.59 | 0.39 | 0.01–4.48 | 0.340 |
| LAMA | Triple therapy with MD ICS | 0.81 | 0.14–3.32 | 1.30 | 0.74–2.44 | 0.535 |
| LAMA | Triple therapy with HD ICS | 1.09 | 0.44–2.51 | 0.89 | 0.08–6.69 | 0.840 |
| Pneumonia | | | | | | |
| Triple therapy with LD ICS | LABA/LAMA | 0.98 | 0.44–2.36 | 1.57 | 1.12–2.14 | 0.316 |
| Triple therapy with MD ICS | LABA/LAMA | 1.79 | 0.77–3.67 | 1.35 | 0.84–2.08 | 0.502 |
| LD ICS/LABA | LABA/LAMA | 0.61 | 0.25–1.62 | 1.55 | 1.08–2.61 | 0.092 |
| MD ICS/LABA | LABA/LAMA | 1.57 | 0.68–3.32 | 1.57 | 1.22–2.66 | 0.996 |
| LAMA | LABA/LAMA | 1.14 | 0.60–2.10 | 0 | 0–1.63 | 0.080 |
| Triple therapy with MD ICS | Triple therapy with LD ICS | 0.77 | 0.37–1.42 | 1.1 | 0.68–1.68 | 0.292 |
| MD ICS/LABA | Triple therapy with LD ICS | 0.82 | 0.44–1.57 | 1.27 | 0.73–2.32 | 0.281 |
| LAMA | Triple therapy with LD ICS | 0.68 | 0.37–1.31 | 1 | 0.17–5.47 | 0.695 |
| LAMA | Triple therapy with MD ICS | 0.60 | 0.06–3.00 | 0.75 | 0.44–1.35 | 0.807 |
| \| Treatment \| Comparator \| Indirect mean difference estimated from NMA \| \| Direct mean difference based estimated from paired meta-analysis \| \| Consistency assumption \| \| --- \| --- \| --- \| --- \| --- \| --- \| --- \| \| **Posterior median mean difference** \| **95% CrIs** \| **Mean difference** \| **95% CIs** \| **P-value** \| | | | | | | |
| Change of trough FEV_1_, ml | | | | | | |
| Triple therapy with LD ICS | LABA/LAMA | 43 | -7.9–93 | 54 | 8.8–100 | 0.685 |
| Triple therapy with MD ICS | LABA/LAMA | 47 | -14–12 | 21 | -41–85 | 0.514 |
| LD ICS/LABA | LABA/LAMA | -99 | -160–-44 | -43 | -88–1.4 | 0.099 |
| MD ICS/LABA | LABA/LAMA | -15 | -89–62 | -45 | -110–18 | 0.503 |
| Triple therapy with MD ICS | Triple therapy with LD ICS | -31 | -82–2 | -25 | -60–1 | 0.841 |
| LAMA | Triple therapy with LD ICS | -110 | -150–-54 | -87 | -150–-25 | 0.624 |
| LAMA | Triple therapy with MD ICS | -57 | -130–12 | -74 | -110–-37 | 0.624 |
| Change of SGRQ score | | | | | | |
| Triple therapy with LD ICS | LABA/LAMA | -1.9 | -4.7–0.84 | -1.7 | -3.3–0.09 | 0.868 |
| Triple therapy with MD ICS | LABA/LAMA | -1.5 | -3.9–1.3 | -1.6 | -3.4–0.36 | 0.925 |
| LD ICS/LABA | LABA/LAMA | -0.87 | -3.4–0.98 | 0.014 | -2.3–2.2 | 0.476 |
| MD ICS/LABA | LABA/LAMA | 0.75 | -2–3.9 | -0.41 | -2.1–1.3 | 0.439 |
| Triple therapy with MD ICS | Triple therapy with LD ICS | -0.21 | -2.5–1.8 | 0.061 | -1.2–1.4 | 0.805 |
| MD ICS/LABA | Triple therapy with LD ICS | 1.8 | 0.15–3.5 | 1.1 | -1.2–3.4 | 0.566 |
| LAMA | Triple therapy with LD ICS | 3.1 | 0.79–5.6 | 3.2 | 0.82–5.6 | 0.948 |
| LAMA | Triple therapy with MD ICS | 3.2 | 0.65–5.8 | 3.1 | 1.1–5.3 | 0.934 |

CI= confidence interval; CrI= credible interval; FEV_1_= forced expiratory volume in one second; HD= high-dose; ICS= inhaled corticosteroid; LABA= long-acting beta-agonist; LAMA= long-acting muscarinic antagonist; LD= low-dose; MD= medium-dose; NMA= network meta-analysis; OR= odds ratio; Sd= standard deviation; SGRQ= St. George's respiratory questionnaire

**Supplementary information 15. Search strategy for the present systematic review and network meta-analysis**

**MEDLINE search strategy**

**#1. COPD patients**

"Lung Diseases, Obstructive"[Mesh:noexp] OR "Pulmonary Disease, Chronic Obstructive"[Mesh] OR "Pulmonary Emphysema"[Mesh] OR "Chronic obstructive pulmonary disease"[tiab] OR "Emphysema"[tiab] OR "Chronic bronchitis"[tiab] OR "Chronic obstructive lung disease"[tiab] OR "Obstructive lung disease"[tiab] OR "Obstructive pulmonary disease"[tiab] OR "Obstructive lung diseases"[tiab] OR "Obstructive pulmonary diseases"[tiab] OR "COPD"[tiab]

**#2. LABA (General)**

"adrenergic beta-2 receptor agonists/administration and dosage"[Mesh] OR "adrenergic beta-2 receptor agonists/adverse effects"[Mesh] OR "adrenergic beta-2 receptor agonists/pharmacology"[Mesh] OR "adrenergic beta-2 receptor agonists/therapeutic use"[Mesh] OR "adrenergic beta-2 receptor agonists/toxicity"[Mesh] OR (("long-acting"[tiab] or "long acting"[tiab] or "ultra-long acting"[tiab] or "ultra-long-acting"[tiab]) **AND** (β agonist*[tiab] OR β-agonist*[tiab] OR β2 agonist*[tiab] OR β2-agonist*[tiab] OR β-2 agonist*[tiab] OR β-2-agonist*[tiab] OR B2 agonist*[tiab] OR B2-agonist*[tiab] OR B-2 agonist*[tiab] OR B-2-agonist*[tiab] OR β(2) agonist*[tiab] OR β(2)-agonist*[tiab] OR β adrenergic agonist*[tiab] OR β-adrenergic agonist*[tiab] OR β2 adrenergic agonist*[tiab] OR β2-adrenergic agonist*[tiab] OR β-2 adrenergic agonist*[tiab] OR β-2-adrenergic agonist*[tiab] OR B2 adrenergic agonist*[tiab] OR B2-adrenergic agonist*[tiab] OR B-2 adrenergic agonist*[tiab] OR B-2-adrenergic agonist*[tiab] OR β(2) adrenergic agonist*[tiab] OR β(2)-adrenergic agonist*[tiab] OR β adrenoceptor agonist*[tiab] OR β-adrenoceptor agonist*[tiab] OR β2 adrenoceptor agonist*[tiab] OR β2-adrenoceptor agonist*[tiab] OR β-2 adrenoceptor agonist*[tiab] OR β-2-adrenoceptor agonist*[tiab] OR B2 adrenoceptor agonist*[tiab] OR B2-adrenoceptor agonist*[tiab] OR B-2 adrenoceptor agonist*[tiab] OR B-2-adrenoceptor agonist*[tiab] OR β(2) adrenoceptor agonist*[tiab] OR β(2)-adrenoceptor agonist*[tiab] OR beta agonist*[tiab] OR beta-agonist*[tiab] OR beta2 agonist*[tiab] OR beta2-agonist*[tiab] OR beta-2 agonist*[tiab] OR beta-2-agonist*[tiab] OR beta(2) agonist*[tiab] OR beta(2)-agonist*[tiab] OR beta adrenergic agonist*[tiab] OR beta-adrenergic agonist*[tiab] OR beta2 adrenergic agonist*[tiab] OR beta2-adrenergic agonist*[tiab] OR beta-2 adrenergic agonist*[tiab] OR beta-2-adrenergic agonist*[tiab] OR beta(2) adrenergic agonist*[tiab] OR beta(2)-adrenergic agonist*[tiab] OR beta adrenoceptor agonist*[tiab] OR beta-adrenoceptor agonist*[tiab] OR beta2 adrenoceptor agonist*[tiab] OR beta2-adrenoceptor agonist*[tiab] OR beta-2 adrenoceptor agonist*[tiab] OR beta-2-adrenoceptor agonist*[tiab] OR beta(2) adrenoceptor agonist*[tiab] OR beta(2)-adrenoceptor agonist*[tiab])) OR "LABA"[tiab] OR "LABAs"[tiab] OR "ultra-LABA"[tiab] OR "ultra-LABAs"[tiab]

**#3. LABA (Individual drugs)**

"Formoterol fumarate"[Mesh] OR "formoterol"[tiab] OR "formoterol-fumarate"[tiab] OR "eformoterol"[tiab] OR "Atock"[tiab] OR "Atimos"[tiab] OR "Foradil"[tiab] OR "Foradile"[tiab] OR "Oxeze"[tiab] OR "Oxis"[tiab] OR "Perforomist"[tiab] OR "BD 40A"[tiab] OR "HSDB 7287"[tiab] OR "5ZZ84GCW8B"[tiab] OR "73573-87-2"[tiab] OR "183814-30-4"[tiab] OR "Indacaterol"[Supplementary Concept] OR "Indacaterol"[tiab] OR "Indacaterol-maleate"[tiab] OR "Onbrez"[tiab] OR "Arcapta"[tiab] OR "QAB 149"[tiab] OR "QAB-149"[tiab] OR "QAB149"[tiab] OR "8OR09251MQ"[tiab] OR "312753-06-3"[tiab] OR "Olodaterol"[Supplementary Concept] OR "Olodaterol"[tiab] OR "Striverdi"[tiab] OR "BI 1744 CL"[tiab] OR "BI 1744"[tiab] OR "BI-1744"[tiab] OR "BI-1744-CL"[tiab] OR "BI1744"[tiab] OR "BI1744CL"[tiab] OR "VD2YSN1AFD"[tiab] OR "868049-49-4"[tiab] OR "Salmeterol Xinafoate"[Mesh] OR "Salmeterol"[tiab] OR "Salmeterol-xinafoate"[tiab] OR "Aeromax"[tiab] OR "Arial"[tiab] OR "Asmerole"[tiab] OR "Astmerole"[tiab] OR "Beglan"[tiab] OR "Betamican"[tiab] OR "Dilamax"[tiab] OR "Inaspir"[tiab] OR "Salmetedur"[tiab] OR "Serevent"[tiab] OR "Ultrabeta"[tiab] OR "GR 33343 X"[tiab] OR "GR 33343-X"[tiab] OR "GR 33343X"[tiab] OR "HSDB 7315"[tiab] OR "SN408D"[tiab] OR "6EW8Q962A5"[tiab] OR "2I4BC502BT"[tiab] OR " 89365-50-4"[tiab] OR " 94749-08-3"[tiab] OR "Vilanterol"[Supplementary Concept] OR ("Vilanterol"[tiab] OR "Vilanterol-trifenatate"[tiab] OR "Ellipta"[tiab] OR "GW 642444M"[tiab] OR "GW 642444x"[tiab] OR "GW-642444M"[tiab] OR "GW-642444x"[tiab] OR "GW642444M"[tiab] OR "GW642444x"[tiab] OR "028LZY775B"[tiab] OR "503068-34-6"[tiab])

**#4. LAMA (General)**

"Muscarinic Antagonists/administration and dosage"[Mesh] OR "Muscarinic Antagonists/adverse effects"[Mesh] OR "Muscarinic Antagonists/pharmacology"[Mesh] OR "Muscarinic Antagonists/therapeutic use"[Mesh] OR "Muscarinic Antagonists/toxicity"[Mesh] OR "Cholinergic Antagonists/administration and dosage"[Mesh] OR "Cholinergic Antagonists/adverse effects"[Mesh] OR "Cholinergic Antagonists/pharmacology"[Mesh] OR "Cholinergic Antagonists/therapeutic use"[Mesh] OR "Cholinergic Antagonists/toxicity"[Mesh] OR (("long-acting"[tiab] or "long acting"[tiab] or "ultra-long acting"[tiab] or "ultra-long-acting"[tiab]) **AND** (muscarinic antagonist*[tiab] OR muscarinic receptor antagonist*[tiab] OR antimuscarinic agent*[tiab] OR anti-muscarinic agent*[tiab] OR muscarinic blocker*[tiab] OR muscarinic receptor blocker*[tiab] OR Cholinergic antagonist*[tiab] OR Cholinergic receptor antagonist*[tiab] OR anticholinergic agent*[tiab] OR anti-cholinergic agent*[tiab] OR cholinergic blocker*[tiab] OR cholinergic receptor blocker*[tiab] OR cholinolytic agent*[tiab])) OR "LAMA"[tiab] OR "LAMAs"[tiab] OR "ultra-LAMA"[tiab] OR "ultra-LAMAs"[tiab]

**#5. LAMA (Individual drugs)**

"Aclidinium bromide"[Supplementary Concept] OR "aclidinium"[tiab] OR "aclidinium-bromide"[tiab] OR "Tudorza"[tiab] OR "Eklira"[tiab] OR "Bretaris"[tiab] OR "LAS 34273"[tiab] OR "LAS W-330"[tiab] OR "UQW7UF9N91"[tiab] OR "320345-99-1"[tiab] OR "Glycopyrrolate"[Mesh] OR "Glycopyrronium"[tiab] OR "Glycopyrronium-bromide"[tiab] OR "Glycopyrrolate"[tiab] OR "Erythro-glycopyrronium"[tiab] OR "NVA237"[tiab] OR "NVA-237"[tiab] OR "Seebri"[tiab] OR "Enurev"[tiab] OR "Tovanor"[tiab] OR "DRM-04"[tiab] OR "DRM04"[tiab] OR "9SFK0PX55W "[tiab] OR "V92SO9WP2I"[tiab] OR "51186-83-5"[tiab] OR "Tiotropium Bromide"[Mesh] OR "Tiotropium"[tiab] OR "Tiotropium-bromide"[tiab] OR "Tiotropium-bromide-monohydrate"[tiab] OR "Spriva"[tiab] OR "BA 679 BR"[tiab] OR "BA-679 BR"[tiab] OR "BA 679BR"[tiab] OR "0EB439235F"[tiab] OR "XX112XZP0J"[tiab] OR "L64SXO195N"[tiab] OR "186691-13-4"[tiab] OR "136310-93-5"[tiab] OR "411207-31-3"[tiab] OR "GSK573719"[Supplementary Concept] OR "Umeclidinium"[tiab] OR "Umeclidinium-bromide"[tiab] OR "Incruse"[tiab] OR "Incruse Ellipta"[tiab] OR "GSK573719"[tiab] OR "GSK-573719"[tiab] OR "GSK573719A"[tiab] OR "7AN603V4JV"[tiab] OR "869113-09-7"[tiab]

**#6. ICS (General)**

(Inhal*[tiab]) **AND** ((("Steroids/administration and dosage"[Mesh] OR "Steroids/adverse effects"[Mesh] OR "Steroids/pharmacology"[Mesh] OR "Steroids/therapeutic use"[Mesh] OR "Steroids/toxicity"[Mesh] OR "Adrenal Cortex Hormones/administration and dosage"[Mesh] OR "Adrenal Cortex Hormones/adverse effects"[Mesh] OR "Adrenal Cortex Hormones/pharmacology"[Mesh] OR "Adrenal Cortex Hormones/therapeutic use"[Mesh] OR "Adrenal Cortex Hormones/toxicity"[Mesh]) **AND** ("Bronchodilator Agents" [Pharmacological Action] OR "Anti-Asthmatic Agents" [Pharmacological Action])) OR corticosteroid*[tiab] OR cortico-steroid*[tiab] OR glucocorticoid*[tiab] OR steroid*[tiab]) OR "ICS"[tiab]

**#7. ICS (Individual drugs)**

"Beclomethasone"[Mesh] OR "beclomethasone 17-monopropionate"[Supplementary Concept] OR "Beclomethasone"[tiab] OR "Beclometasone"[tiab] OR "Beclomethasone-17-monopropionate"[tiab] OR "Beclometasone-17-monopropionate"[tiab] OR "Beclomethasone-dipropionate"[tiab] OR "Beclometasone-dipropionate"[tiab] OR "Beclomethasone-dipropionate-monohydrate"[tiab] OR "Beclometasone-dipropionate-monohydrate"[tiab] OR "Beclodisk"[tiab] OR "Becloforte"[tiab] OR "Beclovent"[tiab] OR "Qvar"[tiab] OR "Vancenase"[tiab] OR "Vanceril"[tiab] OR "4H7L9AI22I"[tiab] OR "5B307S63B2"[tiab] OR "77011-63-3"[tiab] OR "5534-09-8"[tiab] OR "Budesonide"[Mesh] OR "Budesonide"[tiab] OR "Pulmicort"[tiab] OR "Rhinocort"[tiab] OR "Noex"[tiab] OR "Q3OKS62Q6X"[tiab] OR "51333-22-3"[tiab] OR "Fluticasone"[Mesh] OR "fluticasone propionate-17-carboxylic acid" [Supplementary Concept] OR "fluticasone furoate"[Supplementary Concept] OR Fluticason*[tiab] OR "Fluticasone-propionate"[tiab] OR "Fluticasone-furoate"[tiab] OR "CCI 18781"[tiab] OR "GW 685698X"[tiab] OR "GSK 685698"[tiab] OR "Flovent"[tiab] OR "Arnuity"[tiab] OR "O2GMZ0LF5W"[tiab] OR "JS86977WNV"[tiab] OR "80474-14-2"[tiab] OR "397864-44-7"[tiab] OR "Mometasone Furoate"[Mesh]) OR Mometason*[tiab] OR "Mometasone-furoate"[tiab] OR "Mometasone-furoate-monohydrate"[tiab] OR "Sch 32088"[tiab] OR "Asmanex"[tiab] OR "Ecural"[tiab] OR "Elocom"[tiab] OR "Elomet"[tiab] OR "Eziwin"[tiab] OR "MometAid"[tiab] OR "Novasone"[tiab] OR "8HR4QJ6DW8"[tiab] OR "105102-22-5"[tiab] OR "Triamcinolone"[Mesh] OR "Triamcinolone acetonide"[Mesh] OR "Triamcinolone"[tiab] OR "Azmacort"[tiab] OR "F446C597KA"[tiab] OR "1ZK20VI6TY"[tiab] OR "76-25-5"[tiab] OR "124-94-7"[tiab]

**#8. ICS/LABA/LAMA (Mixed drugs)**

"Trimbow"[tiab]

**#9. RCT**

(((((((groups[tiab]) OR (trial[tiab])) OR (randomly[tiab])) OR (drug therapy[sh])) OR (placebo[tiab])) OR (randomized[tiab])) OR (controlled clinical trial[pt])) OR (randomized controlled trial[pt])

**#1 AND (((#2 OR #3) AND (#4 OR #5) AND (#6 OR #7)) OR #8) AND #9**

**Searched references: 835 (June 30, 2022)**

**EMBASE search strategy**

**#1. COPD patients**

'Chronic obstructive lung disease'/exp OR 'Chronic bronchitis'/exp OR 'Lung emphysema'/exp OR 'Chronic obstructive pulmonary disease':ab,ti OR 'Emphysema':ab,ti OR 'Chronic bronchitis':ab,ti OR 'Chronic obstructive lung disease':ab,ti OR 'Obstructive lung disease':ab,ti OR 'Obstructive pulmonary disease':ab,ti OR 'Obstructive lung diseases':ab,ti OR 'Obstructive pulmonary diseases':ab,ti OR 'COPD':ab,ti

**#2. LABA (General)**

'beta 2 adrenergic receptor stimulating agent'/exp OR (('long-acting':ab,ti OR 'long acting':ab,ti OR 'ultra-long acting':ab,ti OR 'ultra-long-acting':ab,ti) **AND** ('β agonist*':ab,ti OR 'β-agonist*':ab,ti OR 'β2 agonist*':ab,ti OR 'β2-agonist*':ab,ti OR 'β-2 agonist*':ab,ti OR 'β-2-agonist*':ab,ti OR 'B2 agonist*':ab,ti OR 'B2-agonist*':ab,ti OR 'B-2 agonist*':ab,ti OR 'B-2-agonist*':ab,ti OR 'β(2) agonist*':ab,ti OR 'β(2)-agonist*':ab,ti OR 'β adrenergic agonist*':ab,ti OR 'β-adrenergic agonist*':ab,ti OR 'β2 adrenergic agonist*':ab,ti OR 'β2-adrenergic agonist*':ab,ti OR 'β-2 adrenergic agonist*':ab,ti OR 'β-2-adrenergic agonist*':ab,ti OR 'B2 adrenergic agonist*':ab,ti OR 'B2-adrenergic agonist*':ab,ti OR 'B-2 adrenergic agonist*':ab,ti OR 'B-2-adrenergic agonist*':ab,ti OR 'β(2) adrenergic agonist*':ab,ti OR 'β(2)-adrenergic agonist*':ab,ti OR 'β adrenoceptor agonist*':ab,ti OR 'β-adrenoceptor agonist*':ab,ti OR 'β2 adrenoceptor agonist*':ab,ti OR 'β2-adrenoceptor agonist*':ab,ti OR 'β-2 adrenoceptor agonist*':ab,ti OR 'β-2-adrenoceptor agonist*':ab,ti OR 'B2 adrenoceptor agonist*':ab,ti OR 'B2-adrenoceptor agonist*':ab,ti OR 'B-2 adrenoceptor agonist*':ab,ti OR 'B-2-adrenoceptor agonist*':ab,ti OR 'β(2) adrenoceptor agonist*':ab,ti OR 'β(2)-adrenoceptor agonist*':ab,ti OR 'beta agonist*':ab,ti OR 'beta-agonist*':ab,ti OR 'beta2 agonist*':ab,ti OR 'beta2-agonist*':ab,ti OR 'beta-2 agonist*':ab,ti OR 'beta-2-agonist*':ab,ti OR 'beta(2) agonist*':ab,ti OR 'beta(2)-agonist*':ab,ti OR 'beta adrenergic agonist*':ab,ti OR 'beta-adrenergic agonist*':ab,ti OR 'beta2 adrenergic agonist*':ab,ti OR 'beta2-adrenergic agonist*':ab,ti OR 'beta-2 adrenergic agonist*':ab,ti OR 'beta-2-adrenergic agonist*':ab,ti OR 'beta(2) adrenergic agonist*':ab,ti OR 'beta(2)-adrenergic agonist*':ab,ti OR 'beta adrenoceptor agonist*':ab,ti OR 'beta-adrenoceptor agonist*':ab,ti OR 'beta2 adrenoceptor agonist*':ab,ti OR 'beta2-adrenoceptor agonist*':ab,ti OR 'beta-2 adrenoceptor agonist*':ab,ti OR 'beta-2-adrenoceptor agonist*':ab,ti OR 'beta(2) adrenoceptor agonist*':ab,ti OR 'beta(2)-adrenoceptor agonist*':ab,ti)) OR 'LABA':ab,ti OR 'LABAs':ab,ti OR 'ultra-LABA':ab,ti OR 'ultra-LABAs':ab,ti

**#3. LABA (Individual drugs)**

'formoterol fumarate'/exp OR 'formoterol':ab,ti OR 'formoterol-fumarate':ab,ti OR 'eformoterol':ab,ti OR 'Atock':ab,ti OR 'Atimos':ab,ti OR 'Foradil':ab,ti OR 'Foradile':ab,ti OR 'Oxeze':ab,ti OR 'Oxis':ab,ti OR 'Perforomist':ab,ti OR 'BD 40A':ab,ti OR 'HSDB 7287':ab,ti OR '5ZZ84GCW8B':ab,ti OR '73573-87-2':ab,ti OR '183814-30-4':ab,ti OR 'indacaterol'/exp OR 'Indacaterol':ab,ti OR 'Indacaterol-maleate':ab,ti OR 'Onbrez':ab,ti OR 'Arcapta':ab,ti OR 'QAB 149':ab,ti OR 'QAB-149':ab,ti OR 'QAB149':ab,ti OR '8OR09251MQ':ab,ti OR '312753-06-3':ab,ti OR 'olodaterol'/exp OR 'Olodaterol':ab,ti OR 'Striverdi':ab,ti OR 'BI 1744 CL':ab,ti OR 'BI 1744':ab,ti OR 'BI-1744':ab,ti OR 'BI-1744-CL':ab,ti OR 'BI1744':ab,ti OR 'BI1744CL':ab,ti OR 'VD2YSN1AFD':ab,ti OR '868049-49-4':ab,ti OR 'salmeterol xinafoate'/exp OR 'Salmeterol':ab,ti OR 'Salmeterol-xinafoate':ab,ti OR 'Aeromax':ab,ti OR 'Arial':ab,ti OR 'Asmerole':ab,ti OR 'Astmerole':ab,ti OR 'Beglan':ab,ti OR 'Betamican':ab,ti OR 'Dilamax':ab,ti OR 'Inaspir':ab,ti OR 'Salmetedur':ab,ti OR 'Serevent':ab,ti OR 'Ultrabeta':ab,ti OR 'GR 33343 X':ab,ti OR 'GR 33343-X':ab,ti OR 'GR 33343X':ab,ti OR 'HSDB 7315':ab,ti OR 'SN408D':ab,ti OR '6EW8Q962A5':ab,ti OR '2I4BC502BT':ab,ti OR '89365-50-4':ab,ti OR '94749-08-3':ab,ti OR 'vilanterol'/exp OR 'vilanterol trifenatate'/exp OR 'Vilanterol':ab,ti OR 'Vilanterol-trifenatate':ab,ti OR 'Ellipta':ab,ti OR 'GW 642444M':ab,ti OR 'GW 642444x':ab,ti OR 'GW-642444M':ab,ti OR 'GW-642444x':ab,ti OR 'GW642444M':ab,ti OR 'GW642444x':ab,ti OR '028LZY775B':ab,ti OR '503068-34-6':ab,ti

**#4. LAMA (General)**

'cholinergic receptor blocking agent'/exp OR 'muscarinic receptor blocking agent'/exp OR (('long-acting':ab,ti or 'long acting':ab,ti or 'ultra-long acting':ab,ti or 'ultra-long-acting':ab,ti) **AND** ('muscarinic antagonist*':ab,ti OR 'muscarinic receptor antagonist*':ab,ti OR 'antimuscarinic agent*':ab,ti OR 'anti-muscarinic agent*':ab,ti OR 'muscarinic blocker*':ab,ti OR 'muscarinic receptor blocker*':ab,ti OR 'Cholinergic antagonist*':ab,ti OR 'Cholinergic receptor antagonist*':ab,ti OR 'anticholinergic agent*':ab,ti OR 'anti-cholinergic agent*':ab,ti OR 'cholinergic blocker*':ab,ti OR 'cholinergic receptor blocker*':ab,ti OR 'cholinolytic agent*':ab,ti)) OR 'LAMA':ab,ti OR 'LAMAs':ab,ti OR 'ultra-LAMA':ab,ti OR 'ultra-LAMAs':ab,ti

**#5. LAMA (Individual drugs)**

'aclidinium bromide'/exp OR 'aclidinium':ab,ti OR 'aclidinium-bromide':ab,ti OR 'Tudorza':ab,ti OR 'Eklira':ab,ti OR 'Bretaris':ab,ti OR 'LAS 34273':ab,ti OR 'LAS W-330':ab,ti OR 'UQW7UF9N91':ab,ti OR '320345-99-1':ab,ti OR 'glycopyrronium'/exp OR 'Glycopyrronium':ab,ti OR 'Glycopyrronium-bromide':ab,ti OR 'Glycopyrrolate':ab,ti OR 'Erythro-glycopyrronium':ab,ti OR 'NVA237':ab,ti OR 'Seebri':ab,ti OR 'Enurev':ab,ti OR 'Tovanor':ab,ti OR 'DRM-04':ab,ti OR 'DRM04':ab,ti OR '9SFK0PX55W':ab,ti OR 'V92SO9WP2I':ab,ti OR '51186-83-5':ab,ti OR 'tiotropium bromide'/exp OR 'Tiotropium':ab,ti OR 'Tiotropium-bromide':ab,ti OR 'Tiotropium-bromide-monohydrate':ab,ti OR 'Spriva':ab,ti OR 'BA 679 BR':ab,ti OR 'BA-679 BR':ab,ti OR 'BA 679BR':ab,ti OR '0EB439235F':ab,ti OR 'XX112XZP0J':ab,ti OR 'L64SXO195N':ab,ti OR '186691-13-4':ab,ti OR '136310-93-5':ab,ti OR '411207-31-3':ab,ti OR 'umeclidinium'/exp OR 'Umeclidinium-bromide':ab,ti OR 'Incruse':ab,ti OR 'Incruse Ellipta':ab,ti OR 'GSK573719':ab,ti OR 'GSK-573719':ab,ti OR 'GSK573719A':ab,ti OR '7AN603V4JV':ab,ti OR '869113-09-7':ab,ti

**#6. ICS (General)**

(('inhal*':ab,ti) **AND** ('glucocorticoid'/exp OR 'corticosteroid':ab,ti OR 'cortico-steroid':ab,ti OR 'glucocorticoid*':ab,ti OR 'steroid*':ab,ti)) OR 'ICS':ab,ti

**#7. ICS (Individual drugs)**

'beclometasone'/exp OR 'beclometasone dipropionate'/exp OR 'Beclomethasone':ab,ti OR 'Beclometasone':ab,ti OR 'Beclomethasone-17-monopropionate':ab,ti OR 'Beclometasone-17-monopropionate':ab,ti OR 'Beclomethasone-dipropionate':ab,ti OR 'Beclometasone-dipropionate':ab,ti OR 'Beclomethasone-dipropionate-monohydrate':ab,ti OR 'Beclometasone-dipropionate-monohydrate':ab,ti OR 'Beclodisk':ab,ti OR 'Becloforte':ab,ti OR 'Beconase':ab,ti OR 'Beclovent':ab,ti OR 'Qvar':ab,ti OR 'Vancenase':ab,ti OR 'Vanceril':ab,ti OR '4H7L9AI22I':ab,ti OR '5B307S63B2':ab,ti OR '77011-63-3':ab,ti OR '5534-09-8':ab,ti OR 'budesonide'/exp OR 'Budesonide':ab,ti OR 'Pulmicort':ab,ti OR 'Rhinocort':ab,ti OR 'Noex':ab,ti OR 'Q3OKS62Q6X':ab,ti OR '51333-22-3':ab,ti OR 'fluticasone'/exp OR 'fluticasone furoate'/exp OR 'fluticasone propionate'/exp OR 'Fluticason*':ab,ti OR 'Fluticasone-propionate':ab,ti OR 'Fluticasone-furoate':ab,ti OR 'CCI 18781':ab,ti OR 'GW 685698X':ab,ti OR 'GSK 685698':ab,ti OR 'Flovent':ab,ti OR 'Arnuity':ab,ti OR 'O2GMZ0LF5W':ab,ti OR 'JS86977WNV':ab,ti OR '80474-14-2':ab,ti OR '397864-44-7':ab,ti OR 'mometasone furoate'/exp OR 'Mometason*':ab,ti OR 'Mometasone-furoate':ab,ti OR 'Mometasone-furoate-monohydrate':ab,ti OR 'Sch 32088':ab,ti OR 'Asmanex':ab,ti OR 'Ecural':ab,ti OR 'Elocom':ab,ti OR 'Elomet':ab,ti OR 'Eziwin':ab,ti OR 'MometAid':ab,ti OR 'Novasone':ab,ti OR '8HR4QJ6DW8':ab,ti OR '105102-22-5':ab,ti OR 'Triamcinolone'/exp OR 'Triamcinolone acetonide'/exp OR 'Triamcinolone':ab,ti OR 'Azmacort':ab,ti OR 'F446C597KA':ab,ti OR '1ZK20VI6TY':ab,ti OR '76-25-5':ab,ti OR '124-94-7':ab,ti

**#8. ICS/LABA/LAMA (Mixed drugs)**

'Trimbow':ab,ti

**#9. RCT**

'crossover procedure'/exp OR 'crossover procedure':ab,ti OR 'double blind procedure'/exp OR 'double blind procedure':ab,ti OR 'randomized controlled trial'/exp OR 'randomized controlled trial':ab,ti OR 'single blind procedure'/exp OR 'single blind procedure':ab,ti OR 'random*':ab,ti OR 'factorial*':ab,ti OR 'crossover*':ab,ti OR 'cross over':ab,ti OR 'cross-over':ab,ti OR 'placebo*':ab,ti OR ('doubl*':ab,ti AND 'blind*':ab,ti) OR ('singl*':ab,ti AND 'blind*':ab,ti) OR 'assign*':ab,ti OR 'allocat*':ab,ti OR 'volunteer*':ab,ti

**#1 AND (((#2 OR #3) AND (#4 OR #5) AND (#6 OR #7)) OR #8) AND #9**

**Searched references: 1,326 (June 30, 2022)**

**Cochrane Library search strategy**

**#1. COPD patients**

MeSH descriptor: [Pulmonary Disease, Chronic Obstructive] explode all trees

OR

Chronic obstructive pulmonary disease:ti,ab,kw OR Emphysema:ti,ab,kw OR Chronic bronchitis:ti,ab,kw OR Chronic obstructive lung disease:ti,ab,kw OR Obstructive lung disease:ti,ab,kw OR Obstructive pulmonary disease:ti,ab,kw OR Obstructive lung diseases:ti,ab,kw OR Obstructive pulmonary diseases:ti,ab,kw OR COPD:ti,ab,kw

**#2. LABA (General)**

MeSH descriptor: [Adrenergic beta-2 Receptor Agonists] explode all trees

OR

((long-acting:ti,ab,kw OR long acting:ti,ab,kw OR ultra-long acting:ti,ab,kw OR ultra-long-acting:ti,ab,kw) **AND** (β agonist*:ti,ab,kw OR β-agonist*:ti,ab,kw OR β2 agonist*:ti,ab,kw OR β-2 agonist*:ti,ab,kw OR B2 agonist*:ti,ab,kw OR B-2 agonist*:ti,ab,kw OR β(2) agonist*:ti,ab,kw OR β adrenergic agonist*:ti,ab,kw OR β-adrenergic agonist*:ti,ab,kw OR β2 adrenergic agonist*:ti,ab,kw OR β-2 adrenergic agonist*:ti,ab,kw OR B2 adrenergic agonist*:ti,ab,kw OR B-2 adrenergic agonist*:ti,ab,kw OR β(2) adrenergic agonist*:ti,ab,kw OR β adrenoceptor agonist*:ti,ab,kw OR β-adrenoceptor agonist*:ti,ab,kw OR β2 adrenoceptor agonist*:ti,ab,kw OR β-2 adrenoceptor agonist*:ti,ab,kw OR B2 adrenoceptor agonist*:ti,ab,kw OR B-2 adrenoceptor agonist*:ti,ab,kw OR β(2) adrenoceptor agonist*:ti,ab,kw OR beta agonist*:ti,ab,kw OR beta-agonist*:ti,ab,kw OR beta2 agonist*:ti,ab,kw OR beta-2 agonist*:ti,ab,kw OR beta(2) agonist*:ti,ab,kw OR beta adrenergic agonist*:ti,ab,kw OR beta-adrenergic agonist*:ti,ab,kw OR beta2 adrenergic agonist*:ti,ab,kw OR beta-2 adrenergic agonist*:ti,ab,kw OR beta(2) adrenergic agonist*:ti,ab,kw OR beta adrenoceptor agonist*:ti,ab,kw OR beta-adrenoceptor agonist*:ti,ab,kw OR beta2 adrenoceptor agonist*:ti,ab,kw OR beta-2 adrenoceptor agonist*:ti,ab,kw OR beta(2) adrenoceptor agonist*:ti,ab,kw)) OR LABA:ti,ab,kw OR LABAs:ti,ab,kw OR ultra-LABA:ti,ab,kw OR ultra-LABAs:ti,ab,kw

**#3. LABA (Individual drugs)**

MeSH descriptor: [Formoterol Fumarate] explode all trees OR MeSH descriptor: [Salmeterol Xinafoate] explode all trees

OR

formoterol:ti,ab,kw OR formoterol-fumarate:ti,ab,kw OR eformoterol:ti,ab,kw OR Atock:ti,ab,kw OR Atimos:ti,ab,kw OR Foradil:ti,ab,kw OR Foradile:ti,ab,kw OR Oxeze:ti,ab,kw OR Oxis:ti,ab,kw OR Perforomist:ti,ab,kw OR BD 40A:ti,ab,kw OR HSDB 7287:ti,ab,kw OR 5ZZ84GCW8B:ti,ab,kw OR Indacaterol:ti,ab,kw OR Indacaterol-maleate:ti,ab,kw OR Onbrez:ti,ab,kw OR Arcapta:ti,ab,kw OR QAB 149:ti,ab,kw OR QAB149:ti,ab,kw OR 8OR09251MQ:ti,ab,kw OR Olodaterol:ti,ab,kw OR Striverdi:ti,ab,kw OR BI 1744 CL:ti,ab,kw OR BI 1744:ti,ab,kw OR BI-1744:ti,ab,kw OR BI1744:ti,ab,kw OR BI1744CL:ti,ab,kw OR VD2YSN1AFD:ti,ab,kw OR Salmeterol:ti,ab,kw OR Salmeterol-xinafoate:ti,ab,kw OR Aeromax:ti,ab,kw OR Arial:ti,ab,kw OR Asmerole:ti,ab,kw OR Astmerole:ti,ab,kw OR Beglan:ti,ab,kw OR Betamican:ti,ab,kw OR Dilamax:ti,ab,kw OR Inaspir:ti,ab,kw OR Salmetedur:ti,ab,kw OR Serevent:ti,ab,kw OR Ultrabeta:ti,ab,kw OR GR 33343 X:ti,ab,kw OR GR 33343X:ti,ab,kw OR HSDB 7315:ti,ab,kw OR SN408D:ti,ab,kw OR 6EW8Q962A5:ti,ab,kw OR 2I4BC502BT:ti,ab,kw OR Vilanterol:ti,ab,kw OR Vilanterol-trifenatate:ti,ab,kw OR Ellipta:ti,ab,kw OR GW 642444M:ti,ab,kw OR GW 642444x:ti,ab,kw OR GW-642444M:ti,ab,kw OR GW-642444x:ti,ab,kw OR GW642444M:ti,ab,kw OR GW642444x:ti,ab,kw OR 028LZY775B:ti,ab,kw

**#4. LAMA (General)**

MeSH descriptor: [Muscarinic Antagonists] explode all trees OR MeSH descriptor: [Cholinergic Antagonists] explode all trees

OR

((long-acting:ti,ab,kw or long acting:ti,ab,kw or ultra-long acting:ti,ab,kw or ultra-long-acting:ti,ab,kw) **AND** (muscarinic antagonist*:ti,ab,kw OR muscarinic receptor antagonist*:ti,ab,kw OR antimuscarinic agent*:ti,ab,kw OR anti-muscarinic agent*:ti,ab,kw OR muscarinic blocker*:ti,ab,kw OR muscarinic receptor blocker*:ti,ab,kw OR Cholinergic antagonist*:ti,ab,kw OR Cholinergic receptor antagonist*:ti,ab,kw OR anticholinergic agent*:ti,ab,kw OR anti-cholinergic agent*:ti,ab,kw OR cholinergic blocker*:ti,ab,kw OR cholinergic receptor blocker*:ti,ab,kw OR cholinolytic agent*:ti,ab,kw)) OR LAMA:ti,ab,kw OR LAMAs:ti,ab,kw OR ultra-LAMA:ti,ab,kw OR ultra-LAMAs:ti,ab,kw

**#5. LAMA (Individual drugs)**

MeSH descriptor: [Glycopyrrolate] explode all trees OR MeSH descriptor: [Tiotropium Bromide] explode all trees

OR

aclidinium:ti,ab,kw OR aclidinium-bromide:ti,ab,kw OR Tudorza:ti,ab,kw OR Eklira:ti,ab,kw OR Bretaris:ti,ab,kw OR LAS 34273:ti,ab,kw OR LAS W-330:ti,ab,kw OR UQW7UF9N91:ti,ab,kw OR Glycopyrronium:ti,ab,kw OR Glycopyrronium-bromide:ti,ab,kw OR Glycopyrrolate:ti,ab,kw OR Erythro-glycopyrronium:ti,ab,kw OR NVA237:ti,ab,kw OR Seebri:ti,ab,kw OR Enurev:ti,ab,kw OR Tovanor:ti,ab,kw OR DRM-04:ti,ab,kw OR DRM04:ti,ab,kw OR 9SFK0PX55W:ti,ab,kw OR V92SO9WP2I:ti,ab,kw OR Tiotropium:ti,ab,kw OR Tiotropium-bromide:ti,ab,kw OR Tiotropium-bromide-monohydrate:ti,ab,kw OR Spriva:ti,ab,kw OR BA 679 BR:ti,ab,kw OR BA-679 BR:ti,ab,kw OR BA 679BR:ti,ab,kw OR 0EB439235F:ti,ab,kw OR XX112XZP0J:ti,ab,kw OR L64SXO195N:ti,ab,kw OR Umeclidinium-bromide:ti,ab,kw OR Incruse:ti,ab,kw OR Incruse Ellipta:ti,ab,kw OR GSK573719:ti,ab,kw OR GSK-573719:ti,ab,kw OR GSK573719A:ti,ab,kw OR 7AN603V4JV:ti,ab,kw

**#6. ICS (General)**

((inhal*:ti,ab,kw) **AND** (((MeSH descriptor: [Steroids] explode all trees OR MeSH descriptor: [Adrenal Cortex Hormones] explode all trees) **AND** (MeSH descriptor: [Bronchodilator Agents] explode all trees OR MeSH descriptor: [Anti-Asthmatic Agents] explode all trees)) OR corticosteroid:ti,ab,kw OR cortico-steroid:ti,ab,kw OR glucocorticoid*:ti,ab,kw OR steroid*:ti,ab,kw)) OR ICS:ti,ab,kw

**#7. ICS (Individual drugs)**

MeSH descriptor: [Beclomethasone] explode all trees OR MeSH descriptor: [Budesonide] explode all trees OR MeSH descriptor: [Fluticasone] explode all trees OR MeSH descriptor: [Mometasone Furoate] explode all trees OR MeSH descriptor: [Triamcinolone] explode all trees OR MeSH descriptor: [Triamcinolone acetonide] explode all trees

OR

Beclomethasone:ti,ab,kw OR Beclometasone:ti,ab,kw Beclomethasone-dipropionate:ti,ab,kw OR Beclometasone-dipropionate:ti,ab,kw OR Beclomethasone-dipropionate-monohydrate:ti,ab,kw OR Beclometasone-dipropionate-monohydrate:ti,ab,kw OR Beclodisk:ti,ab,kw OR Becloforte:ti,ab,kw OR Beconase:ti,ab,kw OR Beclovent:ti,ab,kw OR Qvar:ti,ab,kw OR Vancenase:ti,ab,kw OR Vanceril:ti,ab,kw OR 4H7L9AI22I:ti,ab,kw OR 5B307S63B2:ti,ab,kw OR Budesonide:ti,ab,kw OR Pulmicort:ti,ab,kw OR Rhinocort:ti,ab,kw OR Noex:ti,ab,kw OR Q3OKS62Q6X:ti,ab,kw OR Fluticason*:ti,ab,kw OR Fluticasone-propionate:ti,ab,kw OR Fluticasone-furoate:ti,ab,kw OR CCI 18781:ti,ab,kw OR GW 685698X:ti,ab,kw OR GSK 685698:ti,ab,kw OR Flovent:ti,ab,kw OR Arnuity:ti,ab,kw OR O2GMZ0LF5W:ti,ab,kw OR JS86977WNV:ti,ab,kw OR Mometason*:ti,ab,kw OR Mometasone-furoate:ti,ab,kw OR Mometasone-furoate-monohydrate:ti,ab,kw OR Sch 32088:ti,ab,kw OR Asmanex:ti,ab,kw OR Ecural:ti,ab,kw OR Elocom:ti,ab,kw OR Elomet:ti,ab,kw OR Eziwin:ti,ab,kw OR MometAid:ti,ab,kw OR Novasone:ti,ab,kw OR 8HR4QJ6DW8:ti,ab,kw OR Triamcinolone:ti,ab,kw OR Azmacort:ti,ab,kw OR F446C597KA:ti,ab,kw OR 1ZK20VI6TY:ti,ab,kw

**#8. ICS/LABA/LAMA (Mixed drugs)**

Trimbow:ti,ab,kw

**#9. RCT**

Select "Trials"

**#1 AND (((#2 OR #3) AND (#4 OR #5) AND (#6 OR #7)) OR #8) AND #9**

**Searched references: 917 (June 30, 2022)**
